# Leaving the tropics: The successful colonization of cold temperate regions by *Dolicheremaeus dorni* (Acari, Oribatida)

**DOI:** 10.1111/jzs.12222

**Published:** 2018-03-23

**Authors:** Sylvia Schäffer, Edith Stabentheiner, Satoshi Shimano, Tobias Pfingstl

**Affiliations:** ^1^ Institute of Biology University of Graz Graz Austria; ^2^ Institute of Plant Sciences University of Graz Graz Austria; ^3^ Science Research Center Hosei University Chiyoda‐ku Tokyo Japan

**Keywords:** Carabodoidea, *cytochrome oxidase I*, first Austrian record, haplotype network, tree‐living

## Abstract

Species diversity is generally higher in the tropics compared to the temperate zones. The phenomenon that one species of an almost exclusively tropical living genus was able to adapt successfully to the cold northern regions is rather rare. However, the oribatid mite *Dolicheremaeus dorni* represents such a species and is in the focus of this study. While 180 *Dolicheremaeus* species are confined to the tropics and subtropics, only five species are known to occur in temperate climates and *D. dorni* represents the only species with a wider distribution in this climatic region. This species is distributed in Central and Southern Europe and was now recorded for the first time in Austria. A morphological and molecular genetic investigation of specimens from Austria, Poland and Croatia confirmed this distribution pattern and revealed specific geographic clades and haplotypes for each population and hence indicate low gene flow between populations. A further molecular genetic analysis of the *18S rRNA* gene sequence of *D. dorni* confirmed its phylogenetic position within Carabodoidea. Based on record information, this species is associated with trees or tree habitats and seems to be rather a generalist than a specialist for a specific substrate (e.g., tree species) or food source.

## 1 INTRODUCTION

Species diversity is not homogeneous across the Earth. There are regions, such as the tropics or macrohabitats such as coral reefs, which particularly favor the life of organisms leading to high biological diversity (e.g., Brown, 2014), whereas other areas with rather harsh environmental conditions doubtlessly limit the existence of species (e.g., arctic regions or deserts); however, most others fall somewhere in between (Gaston, 2000). Despite long‐standing studies, causes and/or factors for this increase in species diversity occurring from the poles to the tropics, also known as “latitudinal diversity gradient,” are still unresolved and a universally accepted explanation seems to be a challenging task for the future (Brown, 2014; Condamine, Sperling, Wahlberg, Rasplus, & Kergoat, 2012; Mittelbach et al., 2007; Rohde, 1992). So far, higher species richness in tropical regions could be detected in several groups, for example, in mammals (Rolland, Condamine, Jiguet, & Morlon, 2014), in birds (Ricklefs, 2006), in amphibians (Pyron & Wiens, 2013), in insects (Novotny et al., 2006), and, as shown recently, in oribatid mites too (Pachl et al., 2017). However, in a former study, Maraun, Schatz, and Scheu (2007) demonstrated a non‐linear latitude‐diversity pattern of oribatid mite species diversity as species richness increased from high latitudes to the warm temperate regions but not further to the tropics. In oribatid mites, a good example for high tropic species diversity can be found in the superfamily Carabodoidea which includes five families: the speciose Carabodidae and Otocepheidae, the smaller Nippobodidae and Dampfiellidae (each with two genera), and the monogeneric Tokunocepheidae—a classification scheme based on Norton and Behan‐Pelletier (2009). Most genera have an exclusive tropical distribution excepting a few, as for example, the carabodid *Austrocarabodes, Carabodes* and *Odontocepheus*, or the dampfiellid *Dampfiella* which all are distributed across several climate zones (Norton & Behan‐Pelletier, 2009).

With more than 180 species, *Dolicheremaeus* is the most diverse genus of the family Otocepheidae, which includes 39 described genera (Norton & Behan‐Pelletier, 2009). The preferred habitats of these taxa are wet decaying, spongy woods in tropical regions. As a possible evolutionary adaptation to the high moisture and heavy rainfall present in the tropics, adults of this genus have a special feature allowing to breathe under flooded conditions, namely respiratory taenidia of a type more commonly found in (semi‐)aquatic oribatid mites (Norton & Behan‐Pelletier, 2009; Travé, 1986). Given the large variety of *Dolicheremaeus* species and their more or less exclusive occurrence in tropic areas of the world, cold temperate European biota seem to be unfavorable for these organisms. However, there is one species, namely *Dolicheremaeus dorni* (Balogh, 1937), which has been found sporadically in some, mainly more southern, European countries, for example, Greece, Montenegro, Southern Romania, and Southern France (Balogh, 1937; Mahunka, 1982; Tarman, 1977; Travé, 1986). Additionally, Bulanova‐Zachvatkina (1967) described a species, *Dolicheremaeus georgii,* from the Trans‐Carpathians which is morphologically clearly distinct from *D. dorni*, but since its original description, there were no more findings of this species. Accordingly, *D. dorni* represents the only species of this genus showing a wider distribution in European regions.

Despite there is a huge number of otocepheid species described (more than 400, following the classification of Norton & Behan‐Pelletier, 2009), no barcoding sequences of the mitochondrial *cytochrome c oxidase subunit I* gene (*COI*) are available. However, there are seven sequences of different nuclear markers representing four species of Otocepheidae recorded in GenBank.

In this study, we investigate the geographic distribution and the population structure of *D*. *dorni* and discuss the unusual occurrence of this species in non‐tropical areas. As there is a limit of genetic data in the Otocepheidae, we additionally provide the first *COI* sequences of *D. dorni* and a second otocepheid species *Spinotocepheus* sp., and present their phylogenetic placement within the Oribatida by the use of the standard nuclear *small subunit rRNA* (*18S rRNA*) gene. Furthermore, we integrate a short redescription including leg drawings to the manuscript.

## 2 MATERIAL AND METHODS

### 2.1 Sampling

In total, 49 individuals of *D. dorni* were assayed in this study, whereof all were firstly used for a (at least rough) morphological investigation including body size measurements. Afterward, 14 individuals were used for genetic analyses. All of them were analyzed for a fragment of the *COI* gene for intraspecific studies. Furthermore, one single individual of an undetermined *Spinotocepheus* species was analyzed with the same methods as mentioned before for *D. dorni* individuals. To study the phylogenetic placement, we sequenced part of the *18S rRNA* gene (18S) of one *D. dorni* and the *Spinotocepheus* individual too. These were then aligned together with 54 oribatid mite *18S* sequences from GenBank, including all available sequences of possible sister taxa/groups according to the classification scheme of Norton and Behan‐Pelletier (2009). Species of the supercohort Palaeosomatides are generally considered as the most primitive Oribatida group; therefore, we decided to use them as outgroup taxa. Detailed information on herein investigated individuals is shown in Table 1. Individual data obtained from GenBank are given in the Table A1.

**Table 1:** Sampling locality, coordinates, sample (=voucher) ID, and sequence GenBank accession numbers for all *Dolicheremaeus dorni* (Dd) and *Spinotocepheus sp*. (Spin_sp) specimens analyzed in this study

Sampling locality	Coordinates (North/East)	Sample ID = Voucher ID	GenBank Acc. No.	
			***COI***	***18S rRNA***
Mantscha1 Styria/Austria	47.031403	DdR2_1	MG719354	
	15.366568	DdR2_2	MG719355	
		DdR2_3	MG719356	MG719344
Mantscha2 Styria/Austria	47.025242	DdR14_1	MG719349	
	15.365272			
Mantscha3 Styria/Austria	47.025240	DdR15_1	MG719350	
	15.365269			
Lavamünd Carinthia/Austria	46.614942	DdR53_1	MG719348	
	14.986607	DdR53_2	MG719347	
Litorić Croatia	45.412936	DdR55_1	MG719351	
	15.077517	DdR55_3	MG719352	
		DdR55_4	MG719353	
		DdR55_6	MG719357	
Białowieża Poland	52.739825	DdR88_1	MG719358	
	23.774201	DdR88_2	MG719359	
		DdR88_3	MG719360	
Trang Thailand	7.460046	Spin_sp	MG719346	MG719345
	99.612081			

**Figure 1:**
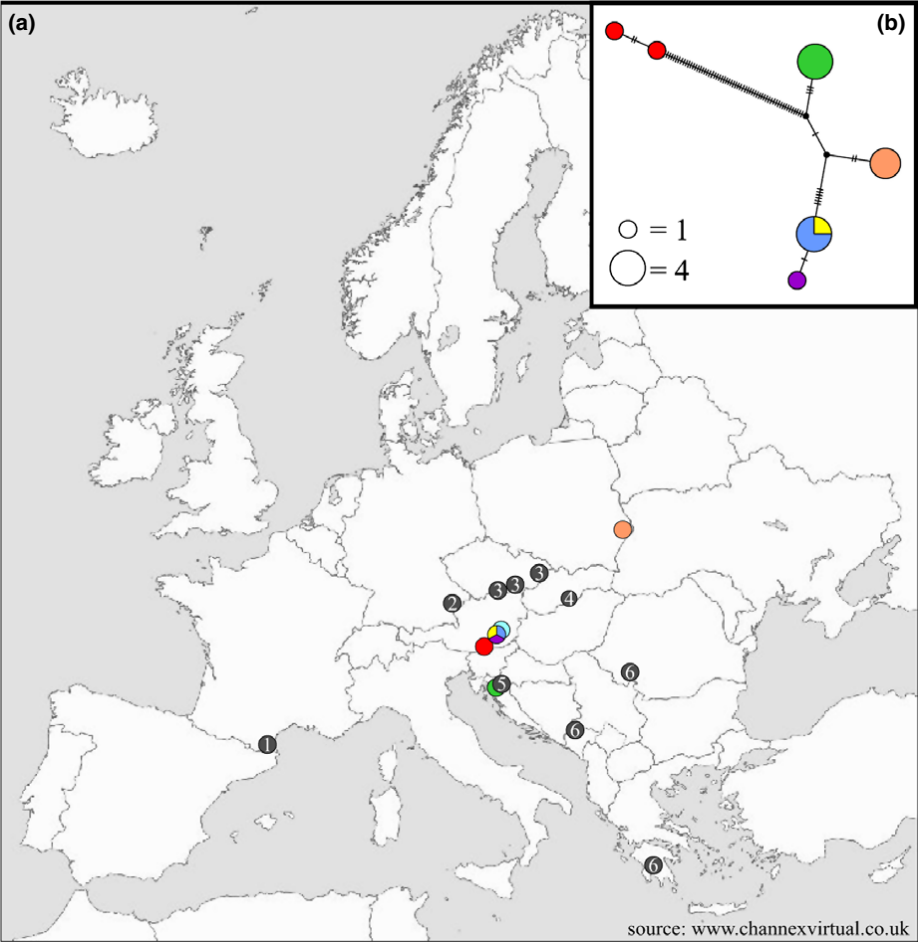
(a) Distribution of *D. dorni* in Europe. Sampling localities of this study are marked by different colors: blue = Mantscha1; green = Litorić; ice blue = Peggau; orange = Białowieża; red = Lavamünd; violet = Mantscha3; and yellow = Mantscha2. Black circles represent data obtained from literature: 1. Travé, 1978; 2. Weigmann, 2014; 3. Miko, 2016; 4. Starý, 1993; 5. Pernek et al., 2012 and 6. Mahunka, Horváth, & Kontschán, 2013. (b) Population structure of 14 studied *D. dorni* specimens using TCS network in PopART. Each circle represents one haplotype. The size of the circle is proportional to the number of individuals belonging to that haplotype. Colors of populations refer to the sampling localities in Figure 1a

Specimens were found in five Norway spruce (*Picea abies* (L.) Karst.) bark samples, damaged by bark beetles, collected from Mantscha (Styria) and Lavamünd (Carinthia) in Austria, from Białowieża in Poland (leg. Dr. Nuria Selva), and from Litorić in Croatia (leg. Dr. Milan Pernek) (see also Figure 1a). *Spinotocepheus* sp. was extracted from leaf litter collected in Trang (Southern Thailand).

For morphological comparisons, we used the specimens described by Weigmann (2014), some specimens collected in a former study from Peggau (preserved in 70% ethanol) and further two individuals deposited in the Senckenberg Museum für Naturkunde Görlitz [collection numbers: 01/42007 Bílé Karpaty Mts., Sidónia Nature Reserve (CZ) and 07/44662 Cerová vrchovina Mts., Hajnáčka (SK)].

Mites were extracted from bark samples using Berlese–Tullgren funnels and then preserved in 100% ethanol for molecular genetic analyses.

### 2.2 Genetic analysis

#### 2.2.1 DNA extraction, amplification, and sequencing

Total genomic DNA of single individuals was extracted by means of the rapid Chelex 100 resin protocol described in Richlen and Barber (2005). Body remnants (cuticle structures) of all investigated specimens were kept and frozen for a later preparation of permanent slides serving as vouchers. All voucher specimens are deposited in the mite collection at the Institute of Zoology, University of Graz (voucher IDs are same as sample IDs; see Table 1).

A 1258‐bp segment of the *COI* gene (including the barcoding region) was amplified in two overlapping fragments using our newly designed primer pairs MiteCOI_fwd1 (5′‐GNTCAACNAATCATWAAGATATTGG‐3′) and MiteCOI_rev2 (5′‐CNTCNGGNTGNCCAAAAAATC‐3′) for the barcoding region (amplicon length 704 bp) and the previously published primers Mite COI‐2F and Mite COI‐2R (Otto & Wilson, 2001) for the subsequent second *COI* region (amplicon length 674 bp). PCR conditions were same as in Schäffer, Krisper, Pfingstl, and Sturmbauer (2008).

Also, PCR amplification of the *18S* sequences was performed in two overlapping fragments of approximately 950 and 1500 bp length each, using the same protocol and primer pairs (18Sfwd/rev960 and fw1230/rev18S) as described in Dabert, Witalinski, Kazmierski, Olszanowski, and Dabert (2010).

Purification of all PCR products and DNA sequencing followed standard protocols as described in Schäffer et al. (2008) using same primers as for PCR amplification. In case of *18S* sequences, two additional internal sequencing primers were used (fw390 and fw770; Dabert et al., 2010). DNA fragments were purified with Sephadex™ G‐50 (Amersham Biosciences) following the manufacturer's instruction and visualized on an ABI PRISM 3130xl automated sequencer (Applied Biosystems). All sequences are available from GenBank with the accession numbers MG719346 to MG719360 for *COI* and MG719344 and MG719345 for *18S* (see also Table 1 & Table A1).

#### 2.2.2 Data analysis

All *COI* sequences were verified by comparisons with known oribatid mite sequences from GenBank and aligned by eye in MEGA version 6 (Tamura, Stecher, Peterson, Filipski, & Kumar, 2013). To infer and visualize the genealogical relationships among the *D. dorni* individuals, the *COI* data were used for a TCS network reconstruction (Clement, Snell, Walker, Posada, & Crandall, 2002) using the program PopART (http://popart.otago.ac.nz).

For *18S* sequence alignment, the R‐Coffee web server (Moretti, Wilm, Higgins, Xenarios, & Notredame, 2008; available at http://www.tcoffee.org) which takes into account the predicted secondary structures, was used. To eliminate poorly aligned positions/regions of the resulted RNA alignment, the program Gblocks v0.91b (Castresana, 2000) was applied under default parameters, except “Minimum Length of A Block” was set to a smaller value (5 instead of 10) as recommended by the authors for rDNA‐like alignments. The final *18S* alignment had a total length of 1375 bp. All alignments are provided as Supporting Information.

Phylogenetic inference was based on maximum likelihood (ML) and Bayesian inference (BI) for the *18S* set, conducted in RAxML v8.2.4 (Stamatakis 2014) and MrBayes 3.1.2 (Ronquist & Huelsenbeck, 2003). For ML, the best‐fit substitution model selected by the corrected Akaike information criterion (AICc) implemented in MEGA was GTR+G+I. Nodes were supported by bootstrapping (1000 replicates). For BI inference, number of substitution types was set to six (GTR model) for each data partition and among‐site rate variation was drawn from a gamma distribution. Posterior probabilities were obtained from a Metropolis‐coupled Markov chain Monte Carlo simulation (two independent runs, eight chains with 15 million generations each, chain temperature 0.2, and trees sampled every 1000 generations). After checking parameter values of the sampled chains in Tracer v1.6 (Rambaut & Drummond, 2007; available at http://tree.bio.ed.ac.uk/software/tracer/), the first 10% of the sampled trees were excluded as burn‐in. From the remaining trees, a majority rule consensus tree was calculated.

### 2.3 Morphological analysis

In general, mite specimens were mounted in Berlese medium (a mixture of arabic gum, aqua dest., glucose, chloral hydrate, and glacial ethanoic acid) as permanent slides.

For scanning electron microscopy, the specimens were dehydrated in ascending ethanol concentrations, air‐dried, mounted on aluminum stubs with double‐sided adhesive tape, and coated with gold. Scanning electron microscopy (SEM) micrographs were taken at the Institute of Plant Sciences with a Philips XL30 ESEM.

## 3 RESULTS

### 3.1 Genetic analyses

#### 3.1.1 COI sequences

In total, six haplotypes were identified in the 14 studied *D. dorni* individuals. Pairwise sequence divergence (uncorrected p‐distance) within the species ranged from 0.2% to 5.8%. According to the TCS network, there was no haplotype sharing between the populations with the exception of individuals from two different trees in the region of Mantscha, which had the same haplotype (yellow and blue colored circle in Figure 1b). Furthermore, the analysis revealed a clear signal for a subdivision of the studied populations into four geographically distinct clades (Figure 1b): one included individuals from Styria/Mantscha (yellow, blue, and violet colored), one from Poland (orange colored), one from Carinthia/Lavamünd (red colored), and one from Croatia (green colored). As the uncorrected pairwise differences between the two studied othocepheid species ranged from 22.3% to 23.2%, we avoided it to include the *Spinotocepheus sp*. haplotype in the network reconstruction.

#### 3.1.2 18S sequences

**Figure 2:**
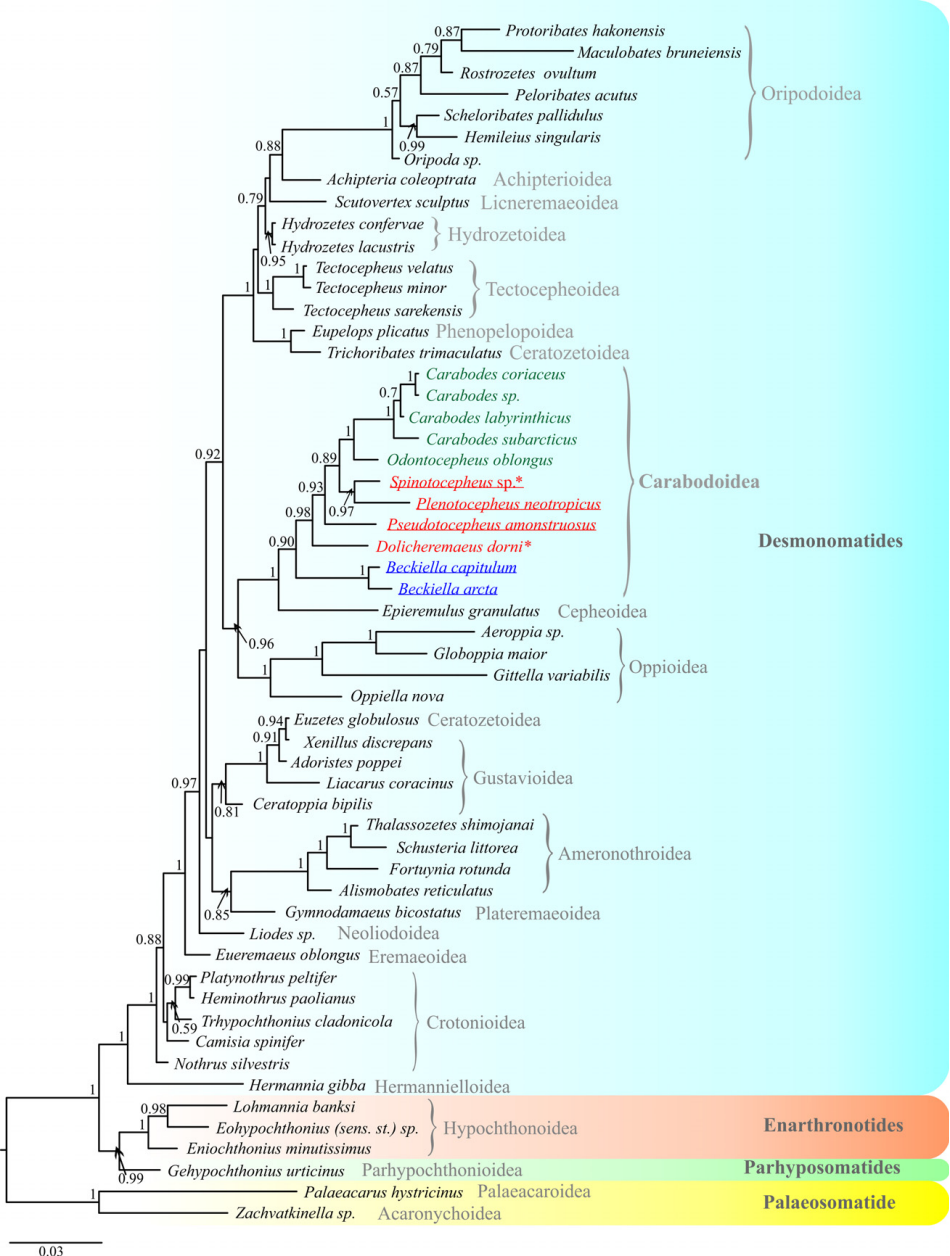
Bayesian inference tree based on the *18S rRNA* gene of oribatid mites. Numbers at nodes represent Bayesian posterior probability values. Only support >0.5 is shown. The families of Carabodoidea are written in different colors: Carabodidae in green, Dampfiellidae in blue, and Otocepheidae in red. Tropic taxa of Carabodoidea are underlined; all others have a temperate distribution. *=sequences are generated in this study

The results of both methods, BI and ML, yielded highly similar topologies (Figure 2 & Figure S1). Differences were either due to unresolved parts in the ML tree compared to the BI analysis (there, however, nodes were poorly supported) or in lower node supports of some taxa. In general, Parhyposomatides and Enarthronotides formed one clade at the basis of the phylogeny with Desmonomatides as sister group which is congruent with previously published data (Dabert et al., 2010; Pachl et al., 2012, 2017). Also within Desmonomatides, the topology went quite along with the morphology‐based system after Norton and Behan‐Pelletier (2009). Nearly all included superfamilies were resolved as monophyletic entities excepting Ceratozetoidea and Crotonioidea—in latter case, however, only weakly supported by both analyses (Figure 2 & Figure S1). Hermannielloidea and Crotoniodea were at the basis of the Desmonomatides, while Licneremaioidea, Achipterioidea, and Oripodoidea were inferred as the most derived ones. Furthermore, BI analysis revealed, with good to high statistical support, monophyly of all included species and/or genera of the Carabodoidea, but paraphyly of one of the three studied families, namely of the Otocepheidae. Moreover, data showed that Cepheoidea [represented by *Epieremulus granulatus* (Balogh & Mahunka, 1979)] were the sister group of Carabodoidea and both together the sister clade of Oppioidea.

### 3.2 Morphology of Austrian *Dolicheremaeus dorni* specimens

Adult. Body length 406‐672 μm (mean 558 μm), width 179–312 μm (mean 250 μm). Specimens investigated: females (*n* = 12), length: 488–672 μm (mean 580 μm), width: 209–312 μm (mean 257 μm); males (*n* = 6), length: 469–594 μm (mean 512 μm), width: 209–269 μm (mean 236 μm).

**Figure 3:**
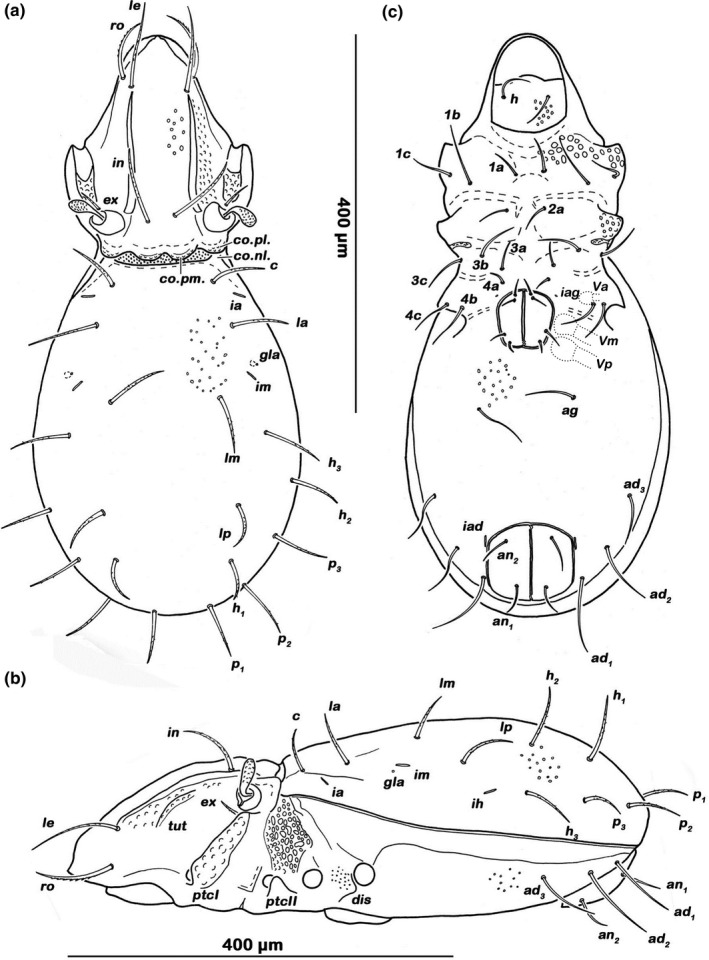
*Dolicheremaeus dorni* adult. (a)—dorsal view; (b)—lateral view, legs as well as epimeral and genital setae omitted; (c)—ventral view, legs omitted

**Figure 4:**
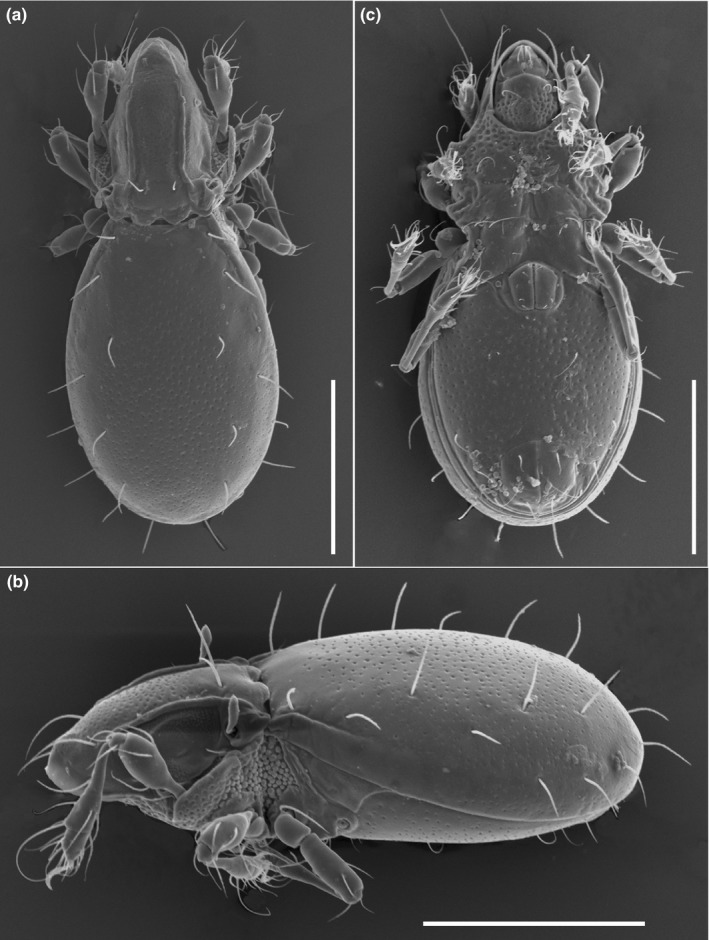
SEM photographs of adult *D. dorni*. Scale bars 200 μm. (a)—dorsal view; (b)—lateral view; (c)—ventral view

Prodorsum (Figures 3a and 4a). Cerotegument finely granular, except for area between costulae showing large granules. All prodorsal setae robust and slightly barbed; rostral setae (*ro*) long (approx. 60 μm), curved inwards, lamellar setae (*le*) same length, interlamellar setae (*in*) slightly shorter (approx. 55 μm), and exobothridial setae (*ex*) the shortest (approx. 20 μm). Posterior edge of prodorsum with two rounded median (*co.pm*.) and two rounded lateral condyles (*co.pl*.) opposing lateral condyles of notogaster (*co.nl*.). Respiratory taenidia present, typical for the genus (see Travé, 1986 p. 88; Figure 1).

Gastronotic region (Figures 3a and 4a). Cerotegument granular, granules loosely distributed. Lateral condyles of notogaster (*co.nl*.) triangular in shape and tips slightly covered by prodorsal lateral condyles (*co.pl*.) in dorsal view. Ten pairs of robust, long (length 55–75 μm), and slightly barbed notogastral setae, *c*,* la*,* lm*,* lp*,* h*_*1‐3*_, *p*_*1‐3*_. Lateral aspect (Figures 3b and 4b). Cerotegument granular, large granules on pedotectum I and II and in lateral sejugal furrow. Pedotectum I (*ptcI*) well developed, reaching lateral edge of bothridium, pedotectum II (*ptcII*) also well developed, triangular in lateral and ventral view. Discidium (*dis*) triangular.

Ventral region of idiosoma (Figures 3c and 4c). Epimeral setation 3‐1‐3‐3, all setae thin and of medium length (approx. 30 μm), except for longer seta *1b* (approx. 40 μm). Four pairs of genital setae, one pair of longer aggenital setae. Median (*Vm*) and posterior genital papillae (*Vp*) normally shaped, whereas anterior papilla (*Va*) smaller and laterally displaced and hence difficult to observe. Posterior median borders of anal valves with interlocking tooth‐like cuticular projection. Two pairs of long anal setae *an*_*1‐2*_ (approx. 20 μm). Anterior and posterior median borders of anal valves with interlocking tooth‐like cuticular projections. Three pairs of long adanal setae *ad*_*1‐3*_ (approx. 55 μm). Seta *ad*_*3*_ slightly anterior of anterior border of anal opening, *ad*_*2*_ and *ad*_*1*_ laterad of anal valves. Lyrifissure *iad* orientated longitudinally, flanking anterior part of anal orifice.

**Figure 5:**
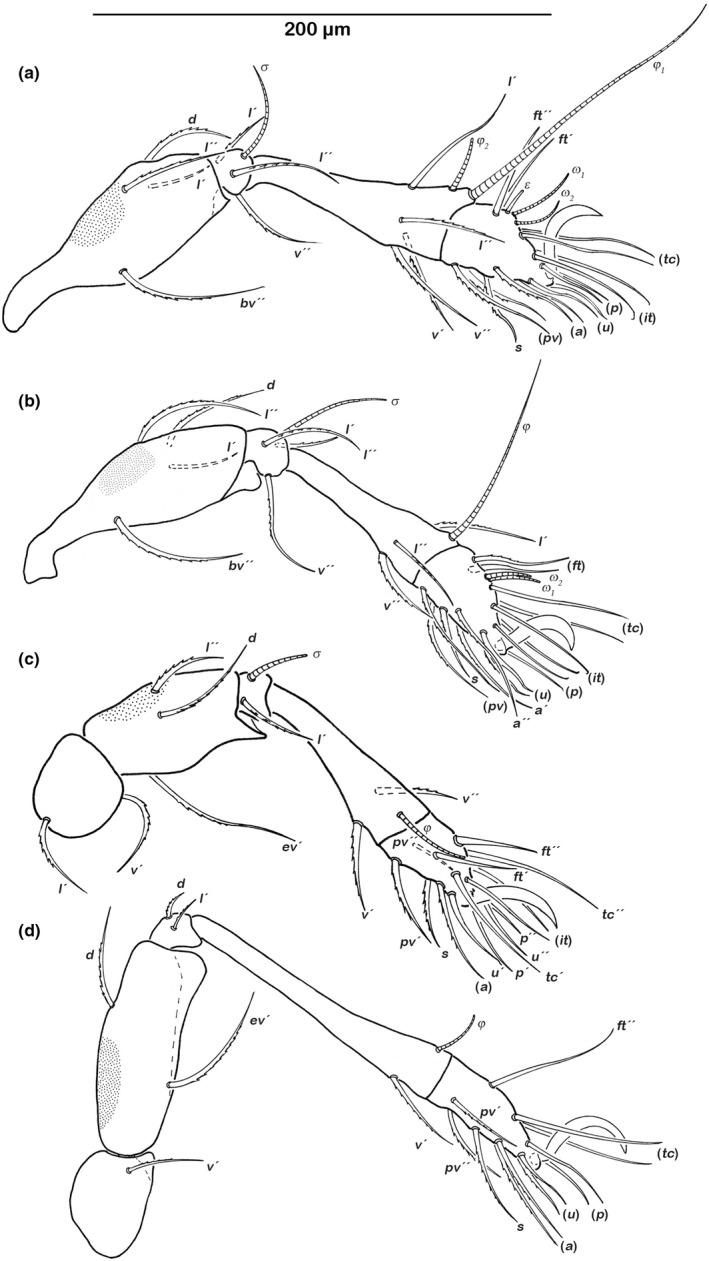
*D. dorni* adult legs antiaxial view. (a)—right leg I; (b)—right leg II; (c)—left leg III; (d)—left leg IV

Legs (Figure 5). Monodactylous. Broad smooth claws. Cerotegument finely granular. Femora with long but slender ventral carinae. Large porose areas on dorsal face of all femora. All setae barbed except for dorsal tarsal setae. Solenidion φ_*1*_ inserted on small apophysis. Tibia of leg IV very slender and nearly a third longer than other tibiae. Chaetome and solenidia see Table S1.

## 4 DISCUSSION

### 4.1 Genetics

All herein investigated *D. dorni* specimens, originating from six European countries, represent one and the same species. This is in contrast to other studies on mites, insects, or other invertebrates, which have shown that presumed widespread taxa often represent complexes of cryptic species (Cicconardi, Fanciulli, & Emerson, 2013; Navia et al., 2013; Pérez‐Portela, Arranz, Rius, & Turon, 2013; Schäffer et al., 2010). However, a clear geographic pattern can be seen in the haplotypes, which means that populations from different geographic locations do not show extensive gene flow between each other and dispersal may be limited. Accordingly, geographic distance is the main isolating factor shaping the population structure of European *D. dorni* populations.

Furthermore, our results revealed similar topologies as already published phylogenies (e.g., Iseki & Karasawa, 2014; Pachl et al., 2012, 2017), aside from the different taxa and taxonomic classification used. According to the system provided by Norton and Behan‐Pelletier (2009), Oppiodea and Gustavioidea might be closely related to the Carabodoidea, which in fact is supported by our phylogenetic data. However, the resulted sister grouping of Carabodoidea and Cepheoidea is questionable, with the reason that the accommodation of the family Anderemaeidae, represented by *E. granulatus* in this study, to the Cepheoidea is still under discussion and therefore might be wrong (Norton & Behan‐Pelletier, 2009; Woas, 2002). Moreover, we call the result of paraphyly of Ceratozetoidea, which is based on the clustering of the ceratozetoid *Euzetes globulus* (Nicolet, 1855) together with species of Gustavioidea, into question as there is no plausible explanation supporting such a grouping. It is more likely that this specimen was originally erroneously identified. Unfortunately, there was no individual of *E. globulus* available for this study, to confirm our suspicion but of course, it has to be checked in the future.

### 4.2 Ecology

Basically all tropical *Dolicheremaeus* species can be found in soil and litter of wet decaying, spongy wood (Norton & Behan‐Pelletier, 2009; Aoki, 1965, 1967; etc.). The temperate *D. dorni* was originally described from decaying leaves in the area of Baile Herculane (Meridional Carpathians) (Balogh, 1937) and Mahunka (1982) examined individuals from soil samples under *Abies cephalonica* Loud. on Mountain Panachaikon (Peloponnese, Greece). However, there are other records documenting individuals of this species collected from bark samples of a beech grove in Massane (Travé, 1978) or from *Fomitopsis pinicola* (Fr.) Karst., a mushroom which colonizes all kind of trees beginning from living to dying or dead ones, in Germany (Maraun, Müller, Bässler, & Scheu, 2014; Weigmann, 2014). Moreover, Murvanidze, Mumladze, Arabuli, Barjadze, and Salakaia (2016) obtained their specimens from dead wood in Georgia and all but one population of the present study were found in bark samples of *P. abies* trees, which were infested by different bark beetle species. In this context, another record of this species seems to be quite interesting, namely those from a study of Pernek, Wirth, Blomquist, Avtzis, and Moser (2012) who detected specimens in samples of the fir bark beetle *Pityokteines curvidens* Germ. caught in pheromone traps in Croatia. However, as this is the only known case of such an association, it would be highly speculative to suggest phoretic behavior for *D. dorni*. Pernek et al. (2012) also stated that the finding of this taxon is more likely the result of accidental dispersal than an active phoretic behavior of the mite (see also Norton, 1980). Moreover, phoresy increases the dispersal ability of individuals leading to positive effects on population demography, evolution, and community success of a species (Clobert, Danchin, Dhondt, & Nichols, 2001). Given the rare and accidental records in Europe (Figure 1a) but also the clear signal of four geographically distinct clades in our network reconstruction (Figure 1b), phoresy seems not to be a common phenomenon in the studied species.

However, as *D. dorni* was found in litter, on bark, and tree‐associated mushrooms, this species clearly seems to be associated with tree habitats but at the same time seems to be a generalist within these habitats. Maybe this generalistic nature is one of the reasons why *D. dorni* could colonize a larger area within cold temperate regions.

### 4.3 Diversity and distributions

Presently, there are 185 species and nine subspecies of the genus *Dolicheremaeus* known worldwide (Subías, 2004). Five species occur in temperate climates, *Dolicheremaeus absolon* (Balogh & Csiszár, 1963), *D. dorni*,* Dolicheremaeus georgii (*Bulanova‐Zachvatkina, 1967), *Dolicheremaeus longipilus* (Higgins & Woolley, 1963), and *Dolicheremaeus montanus* (Krivolutsky, 1971), and eight species in subtropical areas and 178 dwell in the tropics. Four species are distributed across subtropical and tropical climate zones [*Dolicheremaeus distinctus* (Aoki, 1982), *Dolicheremaeus elongatus (*Aoki, 1967), *Dolicheremaeus infrequens* (Aoki, 1967), and *Dolicheremaeus orientalis* (Aoki, 1955)]. Accordingly, there is a clear latitudinal gradient with the lowest species number in temperate regions and the highest number in tropical areas which may be explained by the tropical conservatism hypothesis (Wiens & Donoghue, 2004). This general concept suggests that (i) species richness is higher in tropical biomes because most taxa originated in the tropics, (ii) tropic taxa had more time and area available for speciation, and (iii) species are specialized for tropical climates and only few were able to disperse out of the tropics and adapt to the cold (often freezing) temperatures of middle‐ and high‐latitude regions. These three points are also met by the hypothesis of Pachl et al. (2017) stating that the desmonomatan radiation started on the super continent Pangaea where mites were mainly exposed to tropical climatic conditions.

**Figure 6:**
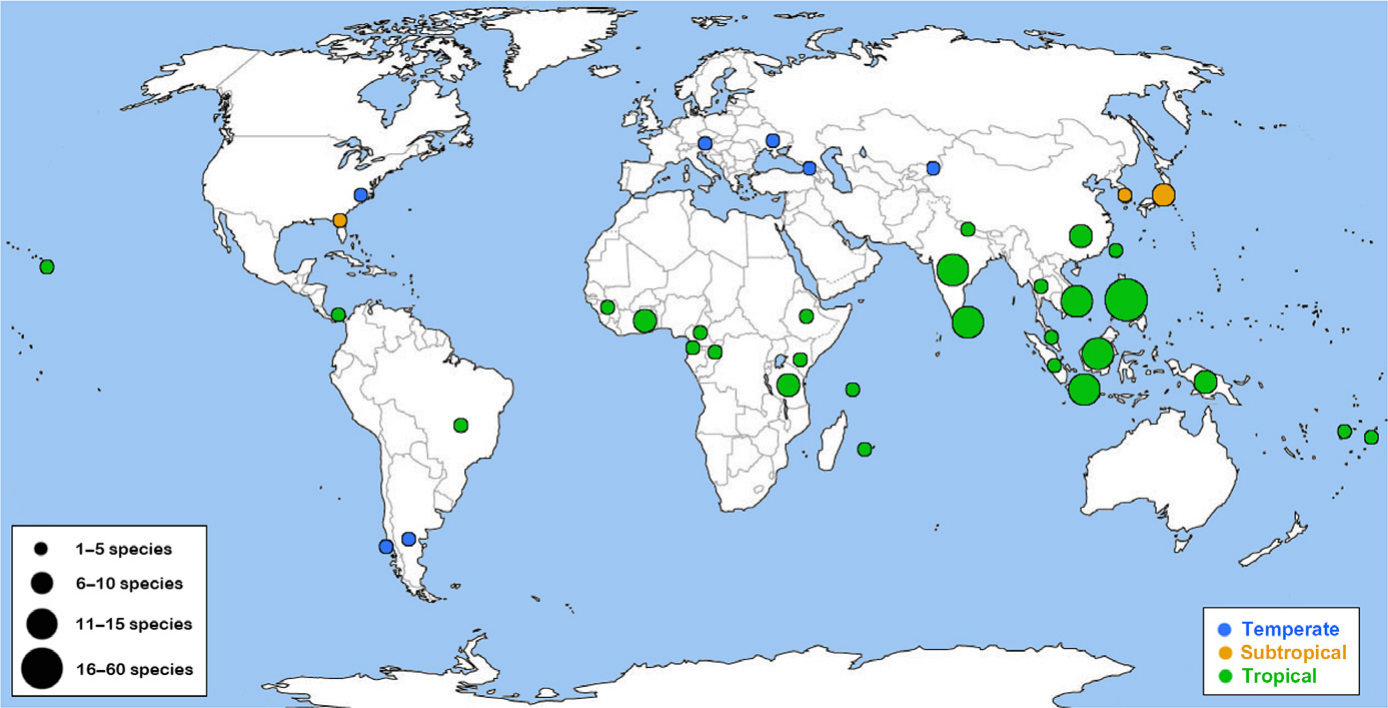
Map showing the worldwide distribution of the genus *Dolicheremaeus*. Colors refer to climate zones; size of circle symbol is relative to species numbers in the respective area

From a geographical point of view, South‐East Asia is the species‐richest area of the world, more than two‐thirds of all *Dolicheremaeus* species are occurring within this region (Figure 6). The reason for this higher number of species is unclear, but this area contains thousands of islands showing a tropical or subtropical climate and hence the high speciation rates may be due to the tremendous amount of ecological niches present within this region (e.g., Hammer & Wallwork, 1979). However, sampling activity has been quite unbalanced across the tropics and large regions of South America and Africa remain uncharted in terms of mite occurrence, and therefore, presently known distribution patterns may not reflect the real distribution of *Dolicheremaeus* in the tropics.

Actually, *D. dorni* has a relatively wide distribution range known within Europe, with Greece as the southernmost point and Poland as the northernmost. Based on the numerous records located in the south of Europe (Figure 1), it may be assumed that *D. dorni* originally dispersed out of the warmer tropical regions into the cold temperate, more northern European areas. However, to infer this colonization pattern and to support the tropical conservatism hypothesis, it is necessary to perform detailed phylogenetic studies of the genus *Dolicheremaeus*, necessarily with various tropical species.

### 4.4 Morphology

The present specimens are well in accordance with the original description of *D. dorni* given by Balogh (1937), but the information Balogh provided lacked important details. Therefore, Weigmann (2014) redescribed this species more extensively based on specimens collected in South‐East Germany. The presently investigated specimens also conform to the latter description except for one morphological feature, namely the size of the epimeral setae. Weigmann stated that the epimeral setae of his individuals were short to moderately long ranging in size from 6 to 25 μm, but the same setae of all presently investigated European populations range from 30 to even 40 μm. Especially, the setae on epimeron I and II are conspicuously shorter in the depicted South‐East German individuals. However, we were able to investigate one of the latter specimens, kindly provided to us by Weigmann, and could not find any conspicuous difference in the length of epimeral setae. The different indication of size given by Weigmann (2014) hence may have been caused by a smaller inclination angle of the setae in his preparation which may result in a shorter appearance. The investigated *D. dorni* specimens clearly possess the same taenidia as shown in the tropical *Dolichermaeus africanus* (Wallwork, 1962) (Travé, 1986) which is an indication that they may also be able to withstand longer periods of inundation. This ability may facilitate the colonization of rain‐soaked dead wood and other similar wet microhabitats.

A comparison of overall body sizes (Table S2) of different European *D. dorni* individuals revealed no obvious deviations, whereas large‐ and small‐sized animals of each population even show the same haplotype. Interestingly, Weigmann (2014) already demonstrated large intraspecific size differences in the German individuals (more than 100 μm between smallest and largest, equaling a fifth of overall body size), and this unusual variation is also present in Austrian and Croatian populations (Table S2). Oribatid mites are known to show a size‐dependent sexual dimorphism with females being basically larger than males (e.g., Behan‐Pelletier, 2015) and the same kind of dimorphism can be found in *D. dorni*. However, males are only by trend smaller and body sizes of both sexes do largely overlap so that the found large variation cannot be explained by such a sexual dimorphism. Jacot described *Dolicheremaeus rubripedes* (Jacot, 1938) and stated “size quite variable” (Jacot, 1938; p.51), which indicates that large intraspecific variation can also be found in other *Dolicheremaeus* species and hence variable body size may simply be a generic trait.

Other possible generic traits which have been neglected so far by most authors may be the presence of porose areas on the legs and the reduced anterior genital papilla. The existence of porose areas on the legs of *D. dorni* is shown here for the first time, but most of the descriptions of *Dolicheremaeus* species are lacking detailed information about the legs and their features and hence an appropriate assessment in terms of distribution and systematic relevance of these structures is not feasible. The same applies to the modified anterior genital papillae which are also mentioned here for the first time in *Dolicheremaeus*. The type of reduction in the anterior genital papillae is similar to that shown in Oppiidae (Behan‐Pelletier, 1991), whereas in the latter, these structures are completely reduced. The similarity in this trait may reflect the close relationship of Carabodoidea and Oppioidea shown in the phylogenetic tree (Figure 2).

However, a comparison with the other two non‐tropical *Dolicheremaeus* species, namely *D. georgii* from Trans‐Carpathians and *D. montanus* from Eastern‐Kirghizia (Ghilarov, 1975), shows that they are quite similar in terms of morphology, and they mainly differ in the shape of notogastral setae and the length of prodorsal lamellae from each other.

## 5 CONCLUSIONS

Morphological and molecular genetic analyses clearly demonstrate that *D. dorni* shows a wider distribution in Central Europe. Nearly all investigated populations show specific haplotypes indicating that there is actually no or low gene flow between the populations. Based on all the records of the temperate *D. dorni*, we suggest that this species is basically associated with tree habitats, whereas preferences for specific tree species or specific microhabitats on the trees could not be detected.

Presently, *D. dorni* represents the only species of this genus that was able to colonize a wider region within the cold temperate climate zone.

## APPENDIX 1

### 1.1

**Table A1:** Species list of included *18S rRNA* sequences obtained from GenBank

Species	Superfamily	Family	GenBank Acc.no.	References
*Achipteria coleoptrata* (Linnaeus, 1758)	Achipterioidea	Achipteriidae	EF091418	Domes, Norton, Maraun, and Scheu (2007)
*Adoristes poppei* (Oudemans, 1906)	Gustavioidea	Liacaridae	EU432202	Maraun et al. (2009)
*Aeroppia sp*.	Oppioidea	Oppiidae	HM070344	Pepato, da Rocha, and Dunlop (2010)
*Alismobates reticulatus* Luxton, 1992	Ameronothroidea	Fortuyniidae	AB818526	Iseki and Karasawa (2014)
*Beckiella arcta* Pérez‐Íñigo & Baggio, 1986	Carabodoidea	Dampfiellidae	KX397628	Krause et al. (2016)
*Beckiella capitulum* Balogh & Mahunka, 1978	Carabodoidea	Dampfiellidae	KR081602	Pachl et al. (2017)
*Camisia spinifer* (Koch, 1836)	Crotonioidea	Camisiidae	EF091420	Domes et al. (2007)
*Carabodes coriaceus* Koch, 1835	Carabodoidea	Carabodidae	EF093787	Laumann et al. (2007)
*Carabodes labyrinthicus* (Michael, 1879)	Carabodoidea	Carabodidae	KX397629	Krause et al. (2016)
*Carabodes sp*.	Carabodoidea	Carabodidae	GQ864283	Dabert et al. (2010)
*Carabodes subarcticus* Trägårdh, 1902	Carabodoidea	Carabodidae	EF091429	Domes et al. (2007)
*Ceratoppia bipilis* (Hermann, 1804)	Gustavioidea	Ceratoppiidae	EU432204	Maraun et al. (2009)
*Eniochthonius minutissimus* (Berlese, 1903)	Hypochthonoidea	Eniochthoniidae	EF091428	Domes et al. (2007)
*Eohypochthonius_sp*	Hypochthonoidea	Hypochthoniidae	JQ000037	Klimov and OConnor (2013)
*Epieremulus granulatus* (Balogh & Mahunka, 1979)	Oppioidea^a^/Cepheoidea	Caleremaeidae^a^/Anderemaeidae	KR081610	Pachl et al. (2017)
*Eueremaeus oblongus* (Koch, 1835)	Eremaeoidea	Eremaeidae	GQ864287	Dabert et al. (2010)
*Eupelops plicatus* (Koch, 1835)	Phenopelopoidea	Phenopelopidae	EF091419	Domes et al. (2007)
*Euzetes globulus* (Nicolet, 1855)	Ceratozetoidea	Euzetidae	AF022030	Thomas (unpublished)
*Fortuynia rotunda* Marshall & Pugh, 2002	Ameronothroidea	Fortuyniidae	AB818525	Iseki and Karasawa (2014)
*Gehypochthonius urticinus* (Berlese, 1910)	Parhypochthonioidea	Gehypochthoniidae	AF022031	Thomas (unpublished)
*Gittella variabilis* Ermilov, Sandmann, Marian & Maraun, 2013	Oppioidea	Oppiidae	KR081612	Pachl et al. (2017)
*Globoppia maior* Hammer, 1962	Oppioidea	Oppiidae	KR081613	Pachl et al. (2017)
*Gymnodamaeus bicostatus* (Koch, 1835)	Gymnodamaeoidea^a^/Plateremaeoidea	Gymnodamaeidae	GQ864285	Dabert et al. (2010)
*Hemileius singularis* (Sellnick, 1930)	Oripodoidea	Hemileiidae	AB818531	Iseki and Karasawa (2014)
*Heminothrus paolianus* (Berlese, 1913)	Crotonioidea	Camisiidae	EF091423	Domes et al. (2007)
*Hermannia gibba* (Koch, 1839)	Hermannioidea^a^/Hermannielloidea	Hermanniidae	EF091426	Domes et al. (2007)
*Hydrozetes confervae* (Schrank, 1781)	Hydrozetoidea	Hydrozetidae	AB818523	Iseki and Karasawa (2014)
*Hydrozetes lacustris* (Michael, 1882)	Hydrozetoidea	Hydrozetidae	EU433987	Schaefer, Norton, Scheu, and Maraun (2010)
*Liacarus coracinus* (Koch, 1841)	Gustavioidea	Liacaridae	KR081619	Pachl et al. (2017)
*Liodes sp*.	Liodoidea^a^/Neoliodoidea	Liodidae	AF022035	Thomas (unpublished)
*Lohmannia banksi* Norton, Metz & Sharma, 1978	Lohmannioidea^a^/Hypochthonoidea	Lohmanniidae	AF022036	Thomas (unpublished)
*Maculobates bruneiensis* Ermilov, Chatterjee & Marshall, 2013	Oripodoidea	Liebstadiidae	AB818522	Iseki and Karasawa (2014)
*Nothrus silvestris* Nicolet, 1855	Crotonioidea	Nothridae	EF091425	Domes et al. (2007)
*Odontocepheus oblongus* (Banks, 1895)	Carabodoidea	Carabodidae	KP325065	Pepato & Klimov (2015)
*Oppiella nova* (Oudemans, 1902)	Oppioidea	Oppiidae	KR081626	Pachl et al. (2017)
*Oripoda sp*.	Oripodoidea	Oripodidae	AB818532	Iseki and Karasawa (2014)
*Palaeacarus hystricinus* Trägårdh, 1932	Palaeacaroidea	Palaeacaridae	EF204472	Schaefer et al. (unpublished)
*Peloribates acutus* Aoki, 1961	Oripodoidea	Haplozetidae	AB818529	Iseki and Karasawa (2014)
*Platynothrus peltifer* (Koch, 1839)	Crotonioidea	Camisiidae	EF091422	Domes et al. (2007)
*Plenotocepheus neotropicus* Ermilov, Sandmann, Marian & Maraun, 2013	Carabodoidea	Tetracondylidae^a^/Otocepheidae	KR081631	Pachl et al. (2017)
*Protoribates hakonensis* Aoki, 1994	Oripodoidea	Protoribatidae	AB818528	Iseki and Karasawa (2014)
*Pseudotocepheus amonstruosus* Mahunka, 1973	Carabodoidea	Otocepheidae	HM070341	Pepato et al. (2010)
*Rostrozetes ovulum* Berlese, 1908	Oripodoidea	Haplozetidae	HM070342	Pepato et al. (2010)
*Scheloribates pallidulus* (Koch, 1841)	Oripodoidea	Scheloribatidae	AB818527	Iseki and Karasawa (2014)
*Schusteria littorea* Grandjean, 1968	Ameronothroidea	Selenoribatidae	HM070345	Pepato et al. (2010)
*Scutovertex sculptus* Michael, 1879	Licneremaeoidea	Scutoverticidae	GQ864305	Dabert et al. (2010)
*Tectocepheus minor* Berlese, 1903	Tectocepheoidea	Tectocepheidae	EF093776	Laumann et al. (2007)
*Tectocepheus sarekensis* Trägårdh, 1910	Tectocepheoidea	Tectocepheidae	EF093781	Laumann et al. (2007)
*Tectocepheus velatus* (Michael, 1880)	Tectocepheoidea	Tectocepheidae	EF093778	Laumann et al. (2007)
*Thalassozetes shimojanai* (Karasawa & Aoki, 2005)	Ameronothroidea	Selenoribatidae	AB818524	Iseki and Karasawa (2014)
*Trhypochthonius cladonicola* (Willmann, 1919)	Trhypochthonioidea^a^/Crotonioidea	Trhypochthoniidae	JQ000047	Klimov and OConnor (2013)
*Trichoribates trimaculatus* (Koch, 1835)	Ceratozetoidea	Ceratozetidae	EU432195	Maraun et al. (2009)
*Xenillus discrepans* Grandjean, 1936	Gustavioidea	Xenillidae	EU432203	Maraun et al. (2009)
*Zachvatkinella_sp*	Acaronychoidea	Archeonothridae	EF203776	Domes, Althammer, Norton, Scheu, and Maraun (2007)

a Classification according to GenBank; classification used in the present study following Norton &Behan‐Pelletier (2009).

## References

[jzs12222-bib-0001] Aoki, J. (1965). A preliminary revision of the family Otocepheidae (Acari, Cryptostigmata) I. Subfamily Otocepheinae. Bulletin of the National Museum of Natural Science, 8, 259–341.

[jzs12222-bib-0002] Aoki, J. (1967). A preliminary revision of the family Otocepheidae (Acari, Cryptostigmata) II. Subfamily Tetracondylinae. Bulletin of the National Museum of Natural Science, 10, 297–359.

[jzs12222-bib-0003] Balogh, J. (1937). *Oppia dorni* spec. nov., eine neue Moosmilben‐Art aus den Südkarpaten. Zoologischer Anzeiger, 119, 221–223.

[jzs12222-bib-0004] Behan‐Pelletier, V. M. (1991). Observations on genital papillae of pycnonotic Brachypylina (Acari: Oribatida). Acarologia, 32, 71–78.

[jzs12222-bib-0005] Behan‐Pelletier, V. M. (2015). Review of sexual dimorphism in brachypyline oribatid mites. Acarologia, 55, 127–146. 10.1051/acarologia/20152163

[jzs12222-bib-0006] Brown, J. H. (2014). Why are there so many species in the tropics? Journal of Biogeography, 41, 8–22. 10.1111/jbi.12228 25684838 PMC4320694

[jzs12222-bib-0007] Bulanova‐Zachvatkina, E. M. (1967) Pancirnyje kleschtschi – Oribatidi. Higher School, Moscow pp 1‐254 [in Russian].

[jzs12222-bib-0008] Castresana, J. (2000). Selection of conserved blocks from multiple alignments for their use in phylogenetic analysis. Molecular Biology and Evolution, 17, 540–552. 10.1093/oxfordjournals.molbev.a026334 10742046

[jzs12222-bib-0009] Cicconardi, F. , Fanciulli, P. P. , & Emerson, B. C. (2013). Collembola, the biological species concept and the underestimation of global species richness. Molecular Ecology, 22, 5382–5396. 10.1111/mec.12472 24112308

[jzs12222-bib-0010] Clement, M. , Snell, Q. , Walker, P. , Posada, D. , & Crandall, K. (2002) TCS: Estimating gene genealogies. Proceedings of the 16th International Parallel and Distributed Processing Symposium (IPDPS ‘02). Washington, DC, USA: IEEE Computer Society. pp 184.

[jzs12222-bib-0011] Clobert, J. , Danchin, E. , Dhondt, A. A. , & Nichols, J. D. (2001). Dispersal (p. 452). New York: Oxford University Press.

[jzs12222-bib-0012] Condamine, F. L. , Sperling, F. A. H. , Wahlberg, N. , Rasplus, J. Y. , & Kergoat, G. J. (2012). What causes latitudinal gradients in species diversity? Evolutionary processes and ecological constraints on swallowtail biodiversity. Ecology Letters, 15, 267–277. 10.1111/j.1461-0248.2011.01737.x 22251895

[jzs12222-bib-0013] Dabert, M. , Witalinski, W. , Kazmierski, A. , Olszanowski, Z. , & Dabert, J. (2010). Molecular phylogeny of acariform mites (Acari, Arachnida): Strong conflict between phylogenetic signal and long‐branch attraction artifacts. Molecular Phylogenetics and Evolution, 56, 222–241. 10.1016/j.ympev.2009.12.020 20060051

[jzs12222-bib-0014] Domes, K. , Althammer, M. , Norton, R. A. , Scheu, S. , & Maraun, M. (2007). The phylogenetic relationship between Astigmata and Oribatida (Acari) as indicated by molecular markers. Experimental and Applied Acarology, 42, 159–171. 10.1007/s10493-007-9088-8 17611803

[jzs12222-bib-0015] Domes, K. , Norton, R. A. , Maraun, M. , & Scheu, S. (2007). Reevolution of sexuality breaks Dollo's law. Proceedings of the National Academy of Sciences of the United States of America, 104, 7139–7144. 10.1073/pnas.0700034104 17438282 PMC1855408

[jzs12222-bib-0016] Gaston, K. J. (2000). Global patterns in biodiversity. Nature, 40, 220–227. 10.1038/35012228 10821282

[jzs12222-bib-0017] Ghilarov, M. S. (1975) A key to the soil‐inhabiting mites. Sarcoptifomes Part I. Moscow, USSR: Nauka. [in Russian] pp 1‐364.

[jzs12222-bib-0018] Hammer, M. , & Wallwork, J. A. (1979). A review of the world distribution of Oribatid mites (Acari: Cryptostigmata) in relation to continental drift. Biologiske Skrifter Dan Vid Selsk, 22, 3–27.

[jzs12222-bib-0019] Iseki, A. , & Karasawa, S. (2014). First record of *Maculobates* (Acari: Oribatida: Liebstadiidae) from Japan, with a Redescription Based on Specimens from the Ryukyu Archipelago. Species Diversity, 19, 59–69. https://doi.org/10.12782/sd.19.1.059

[jzs12222-bib-0020] Jacot, A. P. (1938). The Geenton mites of Florida. Florida Entomologist, 21, 49–57. 10.2307/3492681

[jzs12222-bib-0021] Klimov, P. B. , & OConnor, B. (2013). Is permanent parasitism reversible?—Critical evidence from early evolution of house dust mites. Systematic Biology, 62, 411–423. 10.1093/sysbio/syt008 23417682

[jzs12222-bib-0022] Krause, A. , Pachl, P. , Schulz, G. , Lehmitz, R. , Seniczak, A. , Schaefer, I. , … Maraun, M. (2016). Convergent evolution of aquatic life by sexual and parthenogenetic oribatid mites. Experimental and Applied Acarology, 70, 439–453. 10.1007/s10493-016-0089-3 27785647

[jzs12222-bib-0023] Laumann, M. , Norton, R. A. , Weigmann, G. , Scheu, S. , Maraun, M. , & Heethoff, M. (2007). Speciation in the parthenogenetic oribatid mite genus *Tectocepheus* (Acari, Oribatida) as indicated by molecular phylogeny. Pedobiologia, 51, 111–122.

[jzs12222-bib-0024] Mahunka, S. (1982). Neue und interessante Milben aus der Genfer Museum XXXIX. Fifth Contribution to the Oribatid Fauna of Greece (Acari: Oribatida). Revue Suisse de Zoologie, 89, 497–515. 10.5962/bhl.part.82456

[jzs12222-bib-0025] Mahunka, S. , Horváth, E. , & Kontschán, J. (2013). Oribatid mites of the Balkan Peninsula (Acari: Oribatida). Opuscula Zoologica (Budapest), 44, 11–96.

[jzs12222-bib-0026] Maraun, M. , Erdmann, G. , Schulz, G. , Norton, R. A. , Scheu, S. , & Domes, K. (2009). Multiple convergent evolution of arboreal life in oribatid mites indicates the primacy of ecology. Proceedings. Biological Sciences, 276, 3219–3227. 10.1098/rspb.2009.0425 19535377 PMC2817162

[jzs12222-bib-0027] Maraun, M. , Müller, J. , Bässler, C. , & Scheu, S. (2014). Changes in the community composition and trophic structure of microarthropods in sporocarps of the wood decaying fungus *Fomitopsis pinicola* along an altitudinal gradient. Applied Soil Ecology, 84, 16–23. 10.1016/j.apsoil.2014.06.004

[jzs12222-bib-0028] Maraun, M. , Schatz, H. , & Scheu, S. (2007). Awesome or ordinary? Global diversity patterns of oribatid mites. Ecography, 30, 209–216. 10.1111/j.0906-7590.2007.04994.x

[jzs12222-bib-0029] Miko, L. (2016). Oribatid mites (Acarina: Oribatida) of the Czech Republic. Revised check–list with a proposal for Czech oribatid nomenclature. Klapalekiana, 52, 1–302.

[jzs12222-bib-0030] Mittelbach, G. G. , Schemske, D. W. , Cornell, H. V. , Allen, A. P. , Brown, J. M. , Bush, M. B. , … Turelli, M. (2007). Evolution and the latitudinal diversity gradient: Speciation, extinction and biogeography. Ecology Letters, 10, 315–331. 10.1111/j.1461-0248.2007.01020.x 17355570

[jzs12222-bib-0031] Moretti, S. , Wilm, A. , Higgins, D. G. , Xenarios, I. , & Notredame, C. (2008) R‐Coffee: A web server for accurately aligning noncoding RNA sequences. Nucleic Acids Research, 36 (Web‐Server‐Issue), W10–W13. 10.1093/nar/gkn278 18483080 PMC2447777

[jzs12222-bib-0033] Murvanidze, M. , Mumladze, L. , Arabuli, T. , Barjadze, S. , & Salakaia, M. (2016). Oribatida diversity in different microhabitats of Mtirala National Park. Journal of the Acarological Society of Japan, 25, 35–49. 10.2300/acari.25.Suppl_35

[jzs12222-bib-0034] Navia, D. , Mendonça, R. S. , Ferragut, F. , Miranda, L. C. , Trincado, R. C. , Michaux, J. , & Navajas, M. (2013). Cryptic diversity in *Brevipalpus* mites (Tenuipalpidae). Zoologica. Scripta, 42, 406–426. 10.1111/zsc.12013

[jzs12222-bib-0035] Norton, R. A. (1980). Observations on phoresy by oribatid mites (Acari: Oribatei). International Journal of Acarology, 6, 121–130. 10.1080/01647958008683206

[jzs12222-bib-0036] Norton, R. A. , & Behan‐Pelletier, V. M. (2009). Suborder Oribatida In Krantz G. W., & Walter D. E. (Eds.), A manual of Acarology, 3rd edn. (pp. 430–564). Texas: Texas Tech University Press.

[jzs12222-bib-0037] Novotny, V. , Drozd, P. , Miller, S. E. , Kulfan, M. , Janda, M. , Basset, Y. , & Weiblen, G. D. (2006). Why are there so many species of herbivorous insects in tropical rainforests? Science, 313, 1115–1118. 10.1126/science.1129237 16840659

[jzs12222-bib-0038] Otto, J. C. , & Wilson, K. J. (2001) Assessment of the usefulness of ribosomal 18S and mitochondrial COI sequences in Prostigmata phylogeny In Halliday R. B., Walter D. E., Proctor H. C., Norton R. A. & Colloff J. (Eds.), Acarology (pp 100–109). Proceedings of the 10th International Congress. Melbourne: CSIRO Publishing.

[jzs12222-bib-0039] Pachl, P. , Domes, K. , Schulz, G. , Norton, R. A. , Scheu, S. , Schaefer, I. , & Maraun, M. (2012). Convergent evolution of defense mechanisms in oribatid mites (Acari, Oribatida) shows no “ghosts of predation past”. Molecular Phylogenetics and Evolution, 65, 412–420. 10.1016/j.ympev.2012.06.030 22796481

[jzs12222-bib-0040] Pachl, P. , Lindl, A. C. , Krause, A. , Scheu, S. , Schaefer, I. , & Maraun, M. (2017). The tropics as an ancient cradle of oribatid mite diversity. Acarologia, 57, 309–322. 10.1051/acarologia/20164148

[jzs12222-bib-0041] Pepato, A. R. , da Rocha, C. E. , & Dunlop, J. A. (2010). Phylogenetic position of the acariform mites: Sensitivity to homology assessment under total evidence. BMC Evolutionary Biology, 10, 235 10.1186/1471-2148-10-235 20678229 PMC2933639

[jzs12222-bib-0042] Pérez‐Portela, R. , Arranz, V. , Rius, M. , & Turon, X. (2013). Cryptic speciation or global spread? The case of a cosmopolitan marine invertebrate with limited dispersal capabilities. Scientific Reports, 3, 3197 10.1038/srep03197 24217373 PMC3824166

[jzs12222-bib-0043] Pernek, M. , Wirth, S. , Blomquist, S. R. , Avtzis, D. N. , & Moser, J. C. (2012). New associations of phoretic mites on *Pityokteines curvidens* (Coleoptera, Curculionidae, Scolytinae). Central European Journal of Biology, 7, 63–68.

[jzs12222-bib-0044] Pyron, R. A. , & Wiens, J. J. (2013). Large‐scale phylogenetic analyses reveal the causes of high tropical amphibian diversity. Proceedings. Biological Sciensces, 280, 20131622 10.1098/rspb.2013.1622 PMC3779328 24026818

[jzs12222-bib-0045] Rambaut, A. , & Drummond, A. J. (2007) Tracer—MCMC trace analysis tool. Ver.1.5. Retrieved from http://tree.bio.ed.ac.uk/software/tracer

[jzs12222-bib-0046] Richlen, M. L. , & Barber, P. H. (2005). A technique for the rapid extraction of microalgal DNA from single live and preserved cells. Molecular Ecology Notes, 5, 688–691. 10.1111/j.1471-8286.2005.01032.x

[jzs12222-bib-0047] Ricklefs, R. E. (2006). Global variation in the diversification rate of passerine birds. Ecology, 87, 2468–2478. 10.1890/0012-9658(2006)87[2468:GVITDR]2.0.CO;2 17089656

[jzs12222-bib-0048] Rohde, K. (1992). Latitudinal gradients in species diversity: The search for the primary cause. Oikos, 65, 514–527. 10.2307/3545569

[jzs12222-bib-0049] Rolland, J. , Condamine, F. L. , Jiguet, F. , & Morlon, H. (2014). Faster speciation and reduced extinction in the tropics contribute to the mammalian latitudinal diversity gradient. PLoS Biology, 12, e1001775 10.1371/journal.pbio.1001775 24492316 PMC3904837

[jzs12222-bib-0050] Ronquist, F. , & Huelsenbeck, J. P. (2003). MrBayes 3: Bayesian phylogenetic inference under mixed models. Bioinformatics, 19, 1572–1574. 10.1093/bioinformatics/btg180 12912839

[jzs12222-bib-0051] Schaefer, I. , Norton, R. A. , Scheu, S. , & Maraun, M. (2010). Arthropod colonization of land–Linking molecules and fossils in oribatid mites (Acari, Oribatida). Molecular Phylogenetics and Evolution, 57, 113–121. 10.1016/j.ympev.2010.04.015 20420932

[jzs12222-bib-0052] Schäffer, S. , Krisper, G. , Pfingstl, T. , & Sturmbauer, C. (2008). Description of *Scutovertex pileatus* sp. nov. (Acari, Oribatida, Scutoverticidae) and molecular phylogenetic investigation of congeneric species in Austria. Zoologischer Anzeiger, 247(249–258), 52.

[jzs12222-bib-0053] Schäffer, S. , Pfingstl, T. , Koblmüller, S. , Winkler, K. A. , Sturmbauer, C. , & Krisper, G. (2010). Phylogenetic analysis of European *Scutovertex* mites (Acari, Oribatida, Scutoverticidae) reveals paraphyly and cryptic diversity – a molecular genetic and morphological approach. Mol Phylogenet Evol 55:677‐688. Stamatakis A (2014) RAxML Version 8: A tool for Phylogenetic Analysis and Post‐Analysis of Large Phylogenies. Bioinformatics, 30, 1312–1313. 10.1016/j.ympev.2009.11.025 PMC3935463 20006724

[jzs12222-bib-0054] Starý, J. . (1993) Pancířníci (Acari: Oribatida) Moravskoslezských Beskyd, Česká republika. [Oribatid mites (Acari: Oribatida) from Moravskoslezské Beskydy Mountains, Czech Republic]. Casopis Slezského Zemského Muzea (A). 42:259–266.

[jzs12222-bib-0055] Subías, L. S. (2004) Listado sistemático, sinonímico y biogeográphico de los ácaros oribátidos (Acariformes: Oribatida) del mundo. Graellsia 60:3–305. Update [Internet] 2016 (http://www.ucm.es//info/zoo/Artropodos/Catalogo.pdf)

[jzs12222-bib-0056] Tamura, K. , Stecher, G. , Peterson, D. , Filipski, A. , & Kumar, S. (2013). MEGA6: Molecular Evolutionary Genetics Analysis version 6.0. Molecular Biology and Evolution, 30, 2725–2729. 10.1093/molbev/mst197 24132122 PMC3840312

[jzs12222-bib-0057] Tarman, K. (1977) Južne vrste v oribatidni favni Jugoslavije. (The southern species of the Oribatid fauna in Yugoslavia.) Bioloski vestnik (Ljubljana), 25:63–73.

[jzs12222-bib-0058] Travé, J. (1978). Les stases immatures de *Dolicheremaeus dorni* (Balogh) (Oribate). Acarologia, 20, 294–303.

[jzs12222-bib-0059] Travé, J. (1986). Les taenidies respiratoires des Oribates. Acarologia, 27, 85–94.

[jzs12222-bib-0060] Weigmann, G. (2014). New species of oribatid mites from Southern Germany. Spixiana, 37, 81–88.

[jzs12222-bib-0061] Wiens, J. J. , & Donoghue, M. J. (2004). Historical biogeography, ecology and species richness. Trends in Ecology & Evolution, 19, 639–644. 10.1016/j.tree.2004.09.011 16701326

[jzs12222-bib-0062] Woas, S. (2002). Acari: Oribatida In Adis J. (Ed.), Amazonian arachnida and myriapoda (pp. 21–291). Sofia, Moscow: Pensoft.

