# Supplementary material for: Leaving the tropics: The successful colonization of cold temperate regions by Dolicheremaeus dorni (Acari, Oribatida)
Source: J Zool Syst Evol Res. 2018 Mar 23;56(4):505–18. doi: 10.1111/jzs.12222 (PMC6049615; doi:10.1111/jzs.12222)
Supplement: Supplementary file 1 [file JZS-56-505-s001.pdf]

# Leaving the tropics: the successful colonization of cold temperate regions by *Dolicheremaeus dorni* (Acari, Oribatida)

Sylvia Schäffer*, Edith Stabentheiner, Satoshi Shimano, Tobias Pfingstl

*corresponding author

**Fig. S1**: Maximum likelihood tree based on the *18S rRNA* gene of oribatid mites. Numbers at nodes represent bootstrap values. Only support > 50 is shown. The families of Carabodoidea are written in different colors: Carabodidae in green, Dampfiellidae in blue and Otocepheidae in red. Tropic taxa of Carabodoidea are underlined; all others have a temperate distribution. * = sequences are generated in the present study.

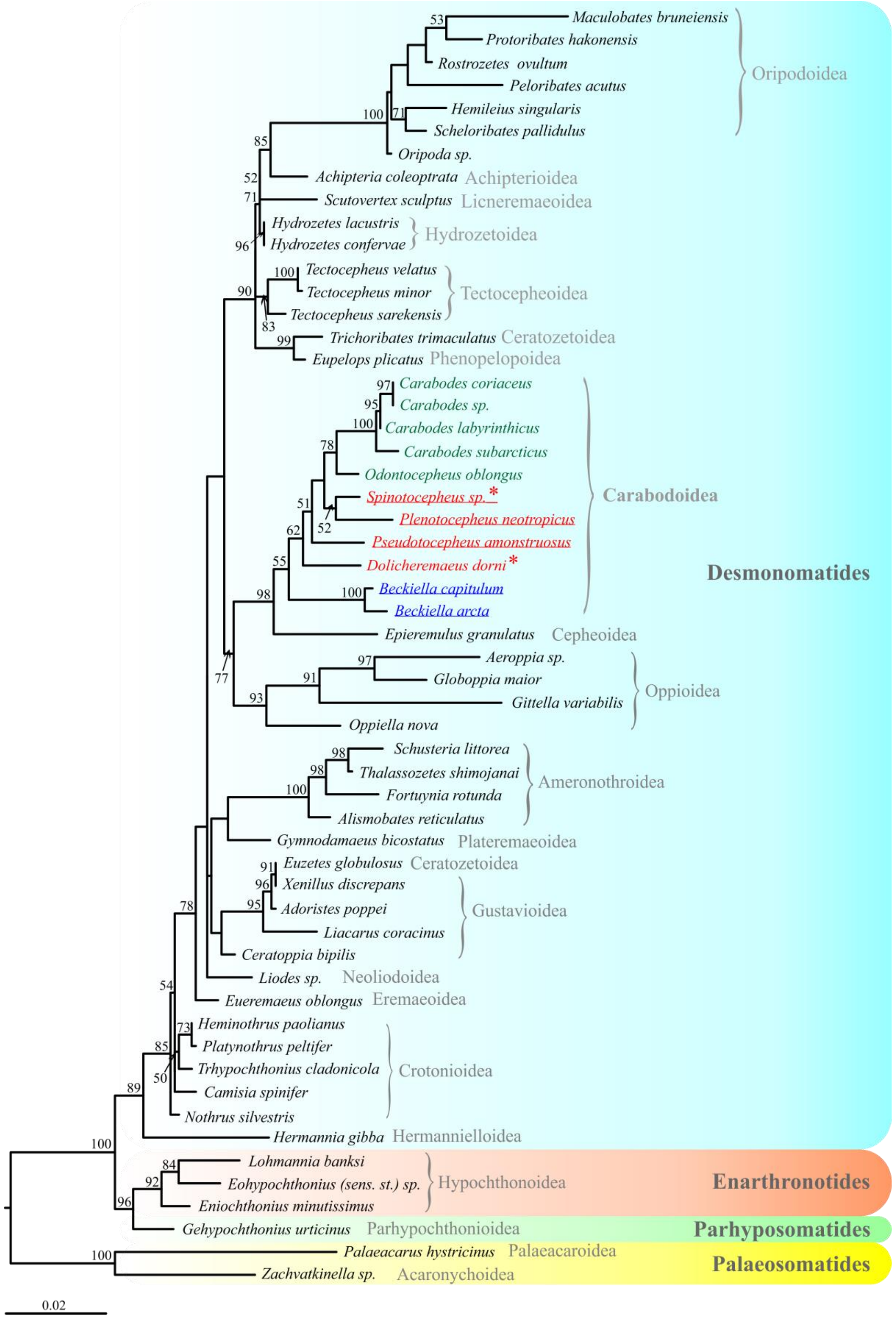

**Table S1:** *Dolicheremaeus dorni* adult, chaetome and solenidia.

| Leg | Trochanter | Femur | Genu | Tibia | Tarsus | Chaetome | Solenidia |
|---|---|---|---|---|---|---|---|
| I | *v´* | *d*, *bv´´*, (*l*) | *v´´*, (*l*), $\sigma$ | (*v*), (*l*), $\varphi_1$, $\varphi_2$ | (*ft*), (*tc*), (*it*), (*p*), (*u*), (*a*), *s*, (*pv*), $\varepsilon$, $\omega_1$, $\omega_2$ | 1-4-3-4-16 | 1-2-2 |
| II | *v´* | *d*, *bv´´*, (*l*) | *v´´*, (*l*), $\sigma$ | *v´´*, (*l*), $\varphi$ | (*ft*), (*tc*), (*it*), (*p*), (*u*), (*a*), *s*, (*pv*), $\omega_1$, $\omega_2$ | 1-4-3-3-15 | 1-1-2 |
| III | *l´*, *v´* | *d*, *ev´* | *l´*, $\sigma$ | (*v*), $\varphi$ | (*ft*), (*tc*), (*it*), (*p*), (*u*), (*a*), *s*, (*pv*) | 2-3-1-2-15 | 1-1-0 |
| IV | *v´* | *d*, *ev´* | *d*, *l´* | *v´*, $\varphi$ | *ft´´*, (*tc*), (*p*), (*u*), (*a*), *s*, (*pv*) | 1-2-2-1-12 | 0-1-0 |

**Table S2**: Comparison of overall body sizes of different European *D. dorni* populations; minimum-maximum (mean ± standard deviation); *information according to literature: Balogh (1937) and Weigmann (2014).

| Origin | n | length (µm) | width (µm) |
|---|---|---|---|
| Mantscha (A) | 16 | 488-625 (572 ± 42.85) | 235-280 (259 ± 14.27) |
| Peggau (A) | 2 | 563-672 (618 ± 77.07) | 250-312 (281 ± 43.84) |
| Lavamünd (A) | 2 | 581-630 (606 ± 34.65) | 250-294 (272 ± 31.11) |
| Litorić (HR) | 24 | 406-660 (537 ± 65.34) | 179-308 (238 ± 30.48) |
| Białowieża (PL) | 3 | 569-625 (604 ± 30.75) | 252-300 (280 ± 24.83) |
| Hajňáčka (SK) | 1 | 575 | 263 |
| Sidónie (CZ) | 1 | 594 | 269 |
| *Băile Herculane (RO) | 1 | 550 | 280 |
| *Grafenau (GER) | 5 | 480-630 | - |

**Alignment of all 15 *COI* sequences generated and analyzed in the present study.** The final alignment had a total of length 1,258 bp.

```
>Spin_sp
AacCaTATAcTtAATTTTAGGATGCTGAGCAgGACTAATAGGATCATCCATAAGAATCTTAATCcGAATAG
AACTTAGACAACCAGGAACACTTCTAGGAAACGATCAAATCTACAACACAATCGTTACAGCTCATGCATTT
ATCATAATTTTCTTCATAGTTATACCCACGATAATTGGAGGAATAGGAAACTGAATGATCCCCCTAATATT
AGGAATTCAAGATATAGCATTCCCTCGAATAAACAATATAAGATTTTGATTACTCCCTCCCTCCTTATTCC
TTCTATGTAATTCTTCACTAGCAAGAGGAGGAGTAGGAACAGGATGAACCGTATACCCTCCACTTTCAAAC
TCCATCTTTCACCATGGAATTTCCGTAGATTTAGCAATCTTCAGGTTGCATATCGCGGGAGTTTCTTCAAT
CCTTGGATCAATCAACTTTATCGCAACAATTATAAATATACGAAGAAAATGCTTAGCACTAGAAACAATTC
CGCTATTCGTATGATCTGTCTTGATTACCACAATCCTTCTATTGCTATCCCTCCCAGTACTCGCAGGGGGG
ATTACCATACTCCTAACAGACCGAAACTTCAACACATCAtTCTTTGATCCATCAgGAgGCGGGGATCCaAT
CTTATACCAACAccTATTTTGATTTTTTGGACatCctGAAGTATACATCcTAATCCTTCCAGGATTTGGGA
TAATTTCACATGTAATCAGATTcTATTCTGGAAAAAAGGAACCATTTGGCAGAATGGGAATAATCTACGCA
ATAGTATCCATTGGATTTCTAGGATTTATTGTATGAGCACACCACATATTCACAGTAGGAATAGATGTAGA
CACACGAGCATATTTTACCGCAGCAACAATAATCATTGCAATCCCCACGGGAGTAAAAGTATTCAGATGAT
TAGCAACCATAAACAACTCCACAATTATAATAGAAACCCCTATGCTATGAGCCATAGGATTCATTATTCTA
TTCACAATTGGAGGATTGACAGGAATTATCTTATCAAACTCATCAATTGATATTAGACTTCATGATACCTA
TTACGTAGTAGGTCACTTTCATTATGTATTATCAATAGGAGCAGTATTTGCGATAATAGCAGGATTAACCC
ACTGGATGCCACTAATTATAAACCTAAACTTCAACCCAAAAATAATAAAATCTCAATTCTACTCCATATTC
ATCGGAGTaAACATAACATTcTTTCCccACCAcTTTCTAgGAcTCAaTgga
>DspR53_2
AACCATGTATTTGGTTTTTGGgggGgTgagcTgGGCTTTTTGGAGGGTCTCTAAGAGTTATTATTCGTCTTG
AATTAGGACAGCCAGGCAGATTGATAGGAAATGACCAGATTTACAATACAATCGTTACAGCACATGCATTC
GTTATAATTTTTTTTATAGTAATACCAATGATAATTGGGGGATTTGGGAATTGATTAGTTCCTTTATTTTT
AGGAAGACAAGACATGGCCTTCCCACGAATAAATAACATAAGATTTTGACTGCTTCCCCCATCTCTAATTC
TACTCCTATTATCTTCAATTTCAGGAAGGGGTGTCGGGACTGGGTGAACTGTGTACCCCCCCTCTATCAGAC
AGAATGTTTCATTACGGAAGATCCGTAGATTTGGCAATCTTTAGGTTACACATTGCAGGAATTTCATCAAT
TTTAGGATCAATTAATTTCATCGCTACCGTAATTAATATACGAATGCCATCTCTATCTATAGAAAACATTC
CTTTATTTGTATGATCTGTTGTAATTACAACAGTGCTTCTACTTCTGTCTCTTCCTGTACTGGCAGGAGGA
ATTACCATATTATTAACAGACCGTAACTTTAATACAACCTTCTTTGATCCTTCAGGAGGGGGGGATCCAAT
CTTATATCAACACCTATTTTGATTTTTTGGACACCCTGAAGTATATATTTTAATTCTACCAGGTTTTGGAG
TAATTTCACATGTAATTAGATTTTATTCAAACAAAAAAGAACCATTTGGAAGCATAGGAATAATCTACGCT
ATGCTATCAATTGGATTTTTAGGTTTTATTGTATGAGCCCATCACATATTTACTGTAGGAATAGATGTAGA
CACACGAGCTTACTTTACAGCAGCTACTATAATTATTGCGATCCCGACAGGAGTAAAGGTATTCAGCTGAT
TAGCAACAATAAACGGATCTTACTTAATAATAGAAGCTCCAATAATATGAGCTTTAGGCTTTATTGTTCTT
TTTACCTTAGGAGGTTTAACAGGGATCGTTTTATCAAATTCTTCAATCGATATCACCCTTCATGATACTTA
TTACGTAGTAGCTCATTTCCATTATGTCCTTTCTATAGGAGCAGTCTTTGCATTAATGGCAGGGTTGACTC
ATTGAATGCCAATAATCATAAATATTCAATTAAACCCTAAAATAGCGAAAGCTCAGTTTTTTTCTATATTT
ATCGGAGTAAACCTAACATTTTTCCCTCAGCACTTTTTAGGATTGAATGGA
>DspR53_1
AACCATGTATTTGGTTTTTGGgGGgTGAGCTGGGCTTTTTGGAGGGTCTCTAAGAGTTATTATTCGTCTTG
AATTAGGACAGCCAGGCAGATTGATAGGAAATGACCAGATTTACAATACAATCGTTACAGCACATGCATTC
GTTATAATTTTTTTTATAGTAATACCAATGATAATTGGGGGATTTGGGAATTGATTGGTTCCTTTATTTTT
AGGAAGACAAGACATGGCCTTCCCACGAATAAATAACATAAGATTTTGACTGCTTCCCCCATCTCTAATTC
TACTCCTATTATCTTCAATTTCAGGAAGGGGTGTCGGGACTGGGTGAACTGTGTACCCCCCCTCTATCAGAC
AGAATGTTTCATTACGGAAGATCCGTAGATTTGGCAATCTTTAGGTTACACATTGCAGGAATTTCATCAAT
TTTAGGATCAATTAATTTCATCGCTACCGTAATTAATATACGAATGCCATCTCTATCTATAGAAAACATTC
CTTTATTTGTATGATCTGTTGTAATTACAACAGTGCTTCTACTTCTGTCTCTTCCTGTACTGGCAGGAGGA
ATTACCATATTATTAACAGACCGTAACTTTAATACAACCTTCTTTGATCCTTCAGGAGGGGGGGATCCAAT
CTTATATCAACACCTATTTTGATTTTTTGGACACCCTGAAGTATATATTTTAATTCTACCAGGTTTTGGAG
TAATTTCACATGTAATTAGATTTTATTCAAACAAAAAAGAACCATTTGGAAGCATAGGAATAATCTACGCT
ATGCTATCAATTGGATTTTTAGGTTTTATTGTATGAGCCCATCACATATTTACTGTAGGAATAGATGTAGA
CACACGAGCTTACTTTACAGCAGCTACTATAATTATTGCGATCCCGACAGGAGTAAAGGTATTCAGCTGAT
TAGCAACAATAAACGGATCTTACTTAATAATAGAAGCTCCAATAATATGAGCTTTAGGCTTTATTGTTCTT
TTTACCTTAGGAGGTTTAACAGGGATCGTTTTATCAAATTCTTCAATCGATATCACCCTTCATGATACTTA
TTACGTAGTAGCTCATTTCCATTATGTCCTTTCTATAGGAGCAGTCTTTGCATTAATGGCAGGGTTGACTC
```

ATTGAATGCCAATAATCATAAATATTCAATTAAACCCTAAAATAGCGAAAGCTCAGTTTTTTTCTATATTT
ATCGGAGTAAACATAACATTTTTCCCTCAGCACTTTTTAGGATTGAATGGA
>DspR14_1
AACCATGTATTTGGTTTTTGGAGGATGAGCCGGGATTTTTGGAGGGTCTCTAAGAGTTATTATTCGTCTTG
AATTAGGACAGCCAGGTAGATTAATAGGGAATGACCAGATCTATAATACAATCGTTACAGCACACGCATTT
GTTATGATTTTTTTTATAGTAATACCAATAATAATTGGGGGATTTGGAAATTGATTGGTTCCTTTATTTTT
AGGAAGACAAGACATAGCATTTCCACGAATAAATAATATAAGATTTTGACTGCTTCCCCCATCTCTAATTT
TACTTTTATTATCTTCAATTTCAGGAAGGGGTGTTGGGACTGGGTGAACTGTGTACCCCCCTCTATCAGAT
AGAATGTTTCACTACGGAAGATCCGTAGATTTGGCAATCTTTAGGTTACACATTGCAGGAATTTCATCAAT
TTTAGGATCAATTAATTTCATCGCTACCGTAATTAATATACGAATACCATCTTTATCTATAGAAAACATTC
CTTTATTTGTATGATCTGTTGTAATTACAACAGTGCTTTTACTTCTATCTCTCCCTGTACTGGCAGGAGGA
ATTACCATATTACTAACAGACCGTAACTTTAATACAACCTTCTTTGATCCTTCGGGAGGAGGGGATCCAAT
CCTATACCAACATTTATTTTGATTTTTTGGACACCCTGAAGTATATATTCTAATTCTACCGGGTTTTGGAG
TAATTTCACATGTAATTAGATTCTATTCAAACAAAAAAGAACCATTTGGAAGTATAGGAATAATCTACGCT
ATACTATCAATTGGATTTTTAGGTTTTATTGTATGAGCCCACCATATATTTACTGTAGGAATAGATGTAGA
CACACGAGCTTACTTTACAGCAGCTACTATAATTATTGCTATTCCGACAGGAGTAAAGGTATTCAGCTGAC
TGGCAACAATAAACAGATCTTACCTAATAATAGAAGCTCCAATAATATGAGCCTTAGGTTTCATTGTTCTT
TTTACCTTAGGAGGTTTAACAGGAATTGTCTTATCAAATTCTTCAATTGATATCACTCTTCATGATACTTA
CTACGTAGTAGCTCATTTTCATTATGTCCTTTCTATAGGAGCAGTTTTTGCATTAATAGCAGGGTTGACTC
ACTGAATGCCAATAATCATAAATATTCAACTAAACCCTAAAATAGCGAAAGCTCAATTTGTTTCTATATTT
ATCGGAGTAAACATGACATTTTTCCCCCAACACTTTTTAGGATTAAATGGA
>DspR15_1
AACCATGTATTTGGTTTTTGGAGGATGAGCCGGGATTTTTGGAGGGTCTCTAAGAGTTATTATTCGTCTTG
AATTAGGACAGCCAGGTAGATTAATAGGGAATGACCAGATCTATAATACAATCGTTACAGCACACGCATTT
GTTATGATTTTTTTTATAGTAATACCAATAATAATTGGGGGATTTGGAAATTGATTGGTTCCTTTATTTTT
AGGAAGACAAGACATAGCATTTCCACGAATAAATAATATAAGATTTTGACTGCTTCCCCCATCTCTAATTT
TACTTTTATTATCTTCAATTTCAGGAAGGGGTGTTGGGACTGGGTGAACTGTGTACCCCCCTCTATCAGAT
AGAATGTTTCACTACGGAAGATCCGTAGATTTGGCAATCTTTAGATTACACATTGCAGGAATTTCATCAAT
TTTAGGATCAATTAATTTCATCGCTACCGTAATTAATATACGAATACCATCTTTATCTATAGAAAACATTC
CTTTATTTGTATGATCTGTTGTAATTACAACAGTGCTTTTACTTCTATCTCTCCCTGTACTGGCAGGAGGA
ATTACCATATTACTAACAGACCGTAACTTTAATACAACCTTCTTTGATCCTTCGGGAGGAGGGGATCCAAT
CCTATACCAACATTTATTTTGATTTTTTGGACACCCTGAAGTATATATTCTAATTCTACCGGGTTTTGGAG
TAATTTCACATGTAATTAGATTCTATTCAAACAAAAAAGAACCATTTGGAAGTATAGGAATAATCTACGCT
ATACTATCAATTGGATTTTTAGGTTTTATTGTATGAGCCCACCATATATTTACTGTAGGAATAGATGTAGA
CACACGAGCTTACTTTACAGCAGCTACTATAATTATTGCTATTCCGACAGGAGTAAAGGTATTCAGCTGAC
TGGCAACAATAAACAGATCTTACCTAATAATAGAAGCTCCAATAATATGAGCCTTAGGTTTCATTGTTCTT
TTTACCTTAGGAGGTTTAACAGGAATTGTCTTATCAAATTCTTCAATTGATATCACTCTTCATGATACTTA
CTACGTAGTAGCTCATTTTCATTATGTCCTTTCTATAGGAGCAGTTTTTGCATTAATAGCAGGGTTGACTC
ACTGAATGCCAATAATCATAAATATTCAACTAAACCCTAAAATAGCGAAAGCTCAATTTGTTTCTATATTT
ATCGGAGTAAACATGACATTTTTCCCCCAACACTTTTTAGGATTAAATGGA
>DspR55_1
AACCATGTATTTGGTTTTTGGAGGATGAGCTGGAATTTTTGGAGGGTCTCTAAGAGTTATTATTCGTCTTG
AATTAGGACAGCCAGGTAGATTAATAGGGAATGACCAGATCTATAATACAATCGTTACAGCACACGCATTT
GTTATAATTTTTTTTATAGTAATACCAATAATAATTGGGGGATTTGGAAATTGATTGGTTCCTTTATTTTT
AGGAAGACAAGACATAGCATTTCCACGAATAAATAATATAAGATTTTGACTACTTCCCCCATCTCTAATTT
TACTTTTATTATCTTCAATTTCAGGAAGGGGTGTTGGGACTGGGTGAACTGTGTACCCCCCTCTATCAGAT
AGAATATTTCACTACGGAAGATCCGTAGATTTGGCAATCTTTAGGTTACACATTGCAGGAATTTCATCAAT
TTTAGGATCAATTAATTTCATCGCTACCGTAATTAATATACGAATACCATCTTTATCTATAGAAAACATCC
CTTTATTTGTATGATCTGTTGTAATTACAACAGTACTTTTACTTCTATCTCTCCCTGTACTGGCAGGAGGA
ATTACCATATTACTAACAGACCGTAACTTTAATACAACCTTCTTTGATCCTTCGGGAGGAGGGGATCCAAT
CCTATACCAACATTTATTTTGATTTTTTGGACACCCTGAAGTATATATTCTAATTCTACCAGGTTTTGGAG
TAATTTCACATGTAATTAGATTCTATTCAAACAAAAAAGAACCATTTGGAAGTATAGGAATAATCTACGCT
ATACTATCAATTGGATTTTTAGGTTTTATTGTATGAGCCCACCATATATTTACTGTAGGAATAGATGTAGA
CACACGAGCTTACTTTACAGCAGCTACTATAATTATTGCTATTCCGACAGGAGTAAAAGTATTCAGCTGAC
TGGCAACAATAAACAGATCTTACCTAATAATAGAAGCTCCAATAATATGAGCCTTAGGTTTCATTGTTCTT
TTTACCTTAGGAGGTTTAACAGGGATTGTCTTATCAAATTCTTCAATTGATATCACTCTTCATGATACTTA
CTACGTAGTAGCTCATTTTCATTATGTCCTTTCTATAGGAGCAGTTTTTGCATTAATAGCAGGGTTGACTC
ACTGAATGCCAATAATCATAAATATTCAACTAAACCCTAAAATAGCGAAAGCTCAATTTGTTTCTATATTT
ATCGGAGTAAACATGACATTTTTCCCCCAACACTTTTTAGGATTAAATGGA
>DspR55_3

AACCATGTATTTGGTTTTTGGAGGATGAGCTGGAATTTTTGGAGGGTCTCTAAGAGTTATTATTCGTCTTG
AATTAGGACAGCCAGGTAGATTAATAGGGAATGACCAGATCTATAATACAATCGTTACAGCACACGCATTT
GTTATAATTTTTTTTATAGTAATACCAATAATAATTGGGGGATTTGGAAATTGATTGGTTCCTTTATTTTT
AGGAAGACAAGACATAGCATTTCCACGAATAAATAATATAAGATTTTGACTACTTCCCCCATCTCTAATTT
TACTTTTATTATCTTCAATTTCAGGAAGGGGTGTTGGGACTGGGTGAACTGTGTACCCCCCTCTATCAGAT
AGAATATTTCACTACGGAAGATCCGTAGATTTGGCAATCTTTAGGTTACACATTGCAGGAATTTCATCAAT
TTTAGGATCAATTAATTTCATCGCTACCGTAATTAATATACGAATACCATCTTTATCTATAGAAAACATCC
CTTTATTTGTATGATCTGTTGTAATTACAACAGTACTTTTACTTCTATCTCTCCCTGTACTGGCAGGAGGA
ATTACCATATTACTAACAGACCGTAACTTTAATACAACCTTCTTTGATCCTTCGGGAGGAGGGGATCCAAT
CCTATACCAACATTTATTTTGATTTTTTGGACACCCTGAAGTATATATTCTAATTCTACCAGGTTTTGGAG
TAATTTCACATGTAATTAGATTCTATTCAAACAAAAAAGAACCATTTGGAAGTATAGGAATAATCTACGCT
ATACTATCAATTGGATTTTTAGGTTTTATTGTATGAGCCCACCATATATTTACTGTAGGAATAGATGTAGA
CACACGAGCTTACTTTACAGCAGCTACTATAATTATTGCTATTCCGACAGGAGTAAAAGTATTCAGCTGAC
TGGCAACAATAAACAGATCTTACCTAATAATAGAAGCTCCAATAATATGAGCCTTAGGTTTCATTGTTCTT
TTTACCTTAGGAGGTTTAACAGGGATTGTCTTATCAAATTCTTCAATTGATATCACTCTTCATGATACTTA
CTACGTAGTAGCTCATTTTCATTATGTCCTTTCTATAGGAGCAGTTTTTGCATTAATAGCAGGGTTGACTC
ACTGAATGCCAATAATCATAAATATTCAACTAAACCCTAAAATAGCGAAAGCTCAATTTGTTTCTATATTT
ATCGGAGTAAACATGACATTTTTCCCCCAACACTTTTTAGGATTAAATGGA
>DspR55_4
AACCATGTATTTGGTTTTTGGAGGATGAGCTGGAATTTTTGGAGGGTCTCTAAGAGTTATTATTCGTCTTG
AATTAGGACAGCCAGGTAGATTAATAGGGAATGACCAGATCTATAATACAATCGTTACAGCACACGCATTT
GTTATAATTTTTTTTATAGTAATACCAATAATAATTGGGGGATTTGGAAATTGATTGGTTCCTTTATTTTT
AGGAAGACAAGACATAGCATTTCCACGAATAAATAATATAAGATTTTGACTACTTCCCCCATCTCTAATTT
TACTTTTATTATCTTCAATTTCAGGAAGGGGTGTTGGGACTGGGTGAACTGTGTACCCCCCTCTATCAGAT
AGAATATTTCACTACGGAAGATCCGTAGATTTGGCAATCTTTAGGTTACACATTGCAGGAATTTCATCAAT
TTTAGGATCAATTAATTTCATCGCTACCGTAATTAATATACGAATACCATCTTTATCTATAGAAAACATCC
CTTTATTTGTATGATCTGTTGTAATTACAACAGTACTTTTACTTCTATCTCTCCCTGTACTGGCAGGAGGA
ATTACCATATTACTAACAGACCGTAACTTTAATACAACCTTCTTTGATCCTTCGGGAGGAGGGGATCCAAT
CCTATACCAACATTTATTTTGATTTTTTGGACACCCTGAAGTATATATTCTAATTCTACCAGGTTTTGGAG
TAATTTCACATGTAATTAGATTCTATTCAAACAAAAAAGAACCATTTGGAAGTATAGGAATAATCTACGCT
ATACTATCAATTGGATTTTTAGGTTTTATTGTATGAGCCCACCATATATTTACTGTAGGAATAGATGTAGA
CACACGAGCTTACTTTACAGCAGCTACTATAATTATTGCTATTCCGACAGGAGTAAAAGTATTCAGCTGAC
TGGCAACAATAAACAGATCTTACCTAATAATAGAAGCTCCAATAATATGAGCCTTAGGTTTCATTGTTCTT
TTTACCTTAGGAGGTTTAACAGGGATTGTCTTATCAAATTCTTCAATTGATATCACTCTTCATGATACTTA
CTACGTAGTAGCTCATTTTCATTATGTCCTTTCTATAGGAGCAGTTTTTGCATTAATAGCAGGGTTGACTC
ACTGAATGCCAATAATCATAAATATTCAACTAAACCCTAAAATAGCGAAAGCTCAATTTGTTTCTATATTT
ATCGGAGTAAACATGACATTTTTCCCCCAACACTTTTTAGGATTAAATGGA
>DspR2_1
AACCATGTATTTGGTTTTTGGAGGATGAGCCGGGATTTTTGGAGGGTCTCTAAGAGTTATTATTCGTCTTG
AATTAGGACAGCCAGGTAGATTAATAGGGAATGACCAGATCTATAATACAATCGTTACAGCACACGCATTT
GTTATGATTTTTTTTATAGTAATACCAATAATAATTGGGGGATTTGGAAATTGATTGGTTCCTTTATTTTT
AGGAAGACAAGACATAGCATTTCCACGAATAAATAATATAAGATTTTGACTGCTTCCCCCATCTCTAATTT
TACTTTTATTATCTTCAATTTCAGGAAGGGGTGTTGGGACTGGGTGAACTGTGTACCCCCCTCTATCAGAT
AGAATGTTTCACTACGGAAGATCCGTAGATTTGGCAATCTTTAGGTTACACATTGCAGGAATTTCATCAAT
TTTAGGATCAATTAATTTCATCGCTACCGTAATTAATATACGAATACCATCTTTATCTATAGAAAACATTC
CTTTATTTGTATGATCTGTTGTAATTACAACAGTGCTTTTACTTCTATCTCTCCCTGTACTGGCAGGAGGA
ATTACCATATTACTAACAGACCGTAACTTTAATACAACCTTCTTTGATCCTTCGGGAGGAGGGGATCCAAT
CCTATACCAACATTTATTTTGATTTTTTGGACACCCTGAAGTATATATTCTAATTCTACCGGGTTTTGGAG
TAATTTCACATGTAATTAGATTCTATTCAAACAAAAAAGAACCATTTGGAAGTATAGGAATAATCTACGCT
ATACTATCAATTGGATTTTTAGGTTTTATTGTATGAGCCCACCATATATTTACTGTAGGAATAGATGTAGA
CACACGAGCTTACTTTACAGCAGCTACTATAATTATTGCTATTCCGACAGGAGTAAAGGTATTCAGCTGAC
TGGCAACAATAAACAGATCTTACCTAATAATAGAAGCTCCAATAATATGAGCCTTAGGTTTCATTGTTCTT
TTTACCTTAGGAGGTTTAACAGGAATTGTCTTATCAAATTCTTCAATTGATATCACTCTTCATGATACTTA
CTACGTAGTAGCTCATTTTCATTATGTCCTTTCTATAGGAGCAGTTTTTGCATTAATAGCAGGGTTGACTC
ACTGAATGCCAATAATCATAAATATTCAACTAAACCCTAAAATAGCGAAAGCTCAATTTGTTTCTATATTT
ATCGGAGTAAACATGACATTTTTCCCCCAACACTTTTTAGGATTAAATGGA
>DspR2_2
AACCATGTATTTGGTTTTTGGAGGATGAGCCGGGATTTTTGGAGGGTCTCTAAGAGTTATTATTCGTCTTG
AATTAGGACAGCCAGGTAGATTAATAGGGAATGACCAGATCTATAATACAATCGTTACAGCACACGCATTT
GTTATGATTTTTTTTATAGTAATACCAATAATAATTGGGGGATTTGGAAATTGATTGGTTCCTTTATTTTT

AGGAAGACAAGACATAGCATTTCCACGAATAAATAATATAAGATTTTGACTGCTTCCCCCATCTCTAATTT
TACTTTTATTATCTTCAATTTCAGGAAGGGGTGTTGGGACTGGGTGAACTGTGTACCCCCCTCTATCAGAT
AGAATGTTTCACTACGGAAGATCCGTAGATTTGGCAATCTTTAGGTTACACATTGCAGGAATTTCATCAAT
TTTAGGATCAATTAATTTCATCGCTACCGTAATTAATATACGAATACCATCTTTATCTATAGAAAACATTC
CTTTATTTGTATGATCTGTTGTAATTACAACAGTGCTTTTACTTCTATCTCTCCCTGTACTGGCAGGAGGA
ATTACCATATTACTAACAGACCGTAACTTTAATACAACCTTCTTTGATCCTTCGGGAGGAGGGGATCCAAT
CCTATACCAACATTTATTTTGATTTTTTGGACACCCTGAAGTATATATTCTAATTCTACCGGGTTTTGGAG
TAATTTCACATGTAATTAGATTCTATTCAAACAAAAAAGAACCATTTGGAAGTATAGGAATAATCTACGCT
ATACTATCAATTGGATTTTTAGGTTTTATTGTATGAGCCCACCATATATTTACTGTAGGAATAGATGTAGA
CACACGAGCTTACTTTACAGCAGCTACTATAATTATTGCTATTCCGACAGGAGTAAAGGTATTCAGCTGAC
TGGCAACAATAAACAGATCTTACCTAATAATAGAAGCTCCAATAATATGAGCCTTAGGTTTCATTGTTCTT
TTTACCTTAGGAGGTTTAACAGGAATTGTCTTATCAAATTCTTCAATTGATATCACTCTTCATGATACTTA
CTACGTAGTAGCTCATTTTCATTATGTCCTTTCTATAGGAGCAGTTTTTGCATTAATAGCAGGGTTGACTC
ACTGAATGCCAATAATCATAAATATTCAACTAAACCCTAAAATAGCGAAAGCTCAATTTGTTTCTATATTT
ATCGGAGTAAACATGACATTTTTCCCCCAACACTTTTTAGGATTAAATGGA
>DspR2_3
AACCATGTATTTGGTTTTTGGAGGATGAGCCGGGATTTTTGGAGGGTCTCTAAGAGTTATTATTCGTCTTG
AATTAGGACAGCCAGGTAGATTAATAGGGAATGACCAGATCTATAATACAATCGTTACAGCACACGCATTT
GTTATGATTTTTTTTATAGTAATACCAATAATAATTGGGGGATTTGGAAATTGATTGGTTCCTTTATTTTT
AGGAAGACAAGACATAGCATTTCCACGAATAAATAATATAAGATTTTGACTGCTTCCCCCATCTCTAATTT
TACTTTTATTATCTTCAATTTCAGGAAGGGGTGTTGGGACTGGGTGAACTGTGTACCCCCCTCTATCAGAT
AGAATGTTTCACTACGGAAGATCCGTAGATTTGGCAATCTTTAGGTTACACATTGCAGGAATTTCATCAAT
TTTAGGATCAATTAATTTCATCGCTACCGTAATTAATATACGAATACCATCTTTATCTATAGAAAACATTC
CTTTATTTGTATGATCTGTTGTAATTACAACAGTGCTTTTACTTCTATCTCTCCCTGTACTGGCAGGAGGA
ATTACCATATTACTAACAGACCGTAACTTTAATACAACCTTCTTTGATCCTTCGGGAGGAGGGGATCCAAT
CCTATACCAACATTTATTTTGATTTTTTGGACACCCTGAAGTATATATTCTAATTCTACCGGGTTTTGGAG
TAATTTCACATGTAATTAGATTCTATTCAAACAAAAAAGAACCATTTGGAAGTATAGGAATAATCTACGCT
ATACTATCAATTGGATTTTTAGGTTTTATTGTATGAGCCCACCATATATTTACTGTAGGAATAGATGTAGA
CACACGAGCTTACTTTACAGCAGCTACTATAATTATTGCTATTCCGACAGGAGTAAAGGTATTCAGCTGAC
TGGCAACAATAAACAGATCTTACCTAATAATAGAAGCTCCAATAATATGAGCCTTAGGTTTCATTGTTCTT
TTTACCTTAGGAGGTTTAACAGGAATTGTCTTATCAAATTCTTCAATTGATATCACTCTTCATGATACTTA
CTACGTAGTAGCTCATTTTCATTATGTCCTTTCTATAGGAGCAGTTTTTGCATTAATAGCAGGGTTGACTC
ACTGAATGCCAATAATCATAAATATTCAACTAAACCCTAAAATAGCGAAAGCTCAATTTGTTTCTATATTT
ATCGGAGTAAACATGACATTTTTCCCCCAACACTTTTTAGGATTAAATGGA
>DspR55_6
AACCATGTATTTGGTTTTTGGAGGATGAGCTGGAATTTTTGGAGGGTCTCTAAGAGTTATTATTCGTCTTG
AATTAGGACAGCCAGGTAGATTAATAGGGAATGACCAGATCTATAATACAATCGTTACAGCACACGCATTT
GTTATAATTTTTTTTATAGTAATACCAATAATAATTGGGGGATTTGGAAATTGATTGGTTCCTTTATTTTT
AGGAAGACAAGACATAGCATTTCCACGAATAAATAATATAAGATTTTGACTACTTCCCCCATCTCTAATTT
TACTTTTATTATCTTCAATTTCAGGAAGGGGTGTTGGGACTGGGTGAACTGTGTACCCCCCTCTATCAGAT
AGAATATTTCACTACGGAAGATCCGTAGATTTGGCAATCTTTAGGTTACACATTGCAGGAATTTCATCAAT
TTTAGGATCAATTAATTTCATCGCTACCGTAATTAATATACGAATACCATCTTTATCTATAGAAAACATCC
CTTTATTTGTATGATCTGTTGTAATTACAACAGTACTTTTACTTCTATCTCTCCCTGTACTGGCAGGAGGA
ATTACCATATTACTAACAGACCGTAACTTTAATACAACCTTCTTTGATCCTTCGGGAGGAGGGGATCCAAT
CCTATACCAACATTTATTTTGATTTTTTGGACACCCTGAAGTATATATTCTAATTCTACCAGGTTTTGGAG
TAATTTCACATGTAATTAGATTCTATTCAAACAAAAAAGAACCATTTGGAAGTATAGGAATAATCTACGCT
ATACTATCAATTGGATTTTTAGGTTTTATTGTATGAGCCCACCATATATTTACTGTAGGAATAGATGTAGA
CACACGAGCTTACTTTACAGCAGCTACTATAATTATTGCTATTCCGACAGGAGTAAAAGTATTCAGCTGAC
TGGCAACAATAAACAGATCTTACCTAATAATAGAAGCTCCAATAATATGAGCCTTAGGTTTCATTGTTCTT
TTTACCTTAGGAGGTTTAACAGGGATTGTCTTATCAAATTCTTCAATTGATATCACTCTTCATGATACTTA
CTACGTAGTAGCTCATTTTCATTATGTCCTTTCTATAGGAGCAGTTTTTGCATTAATAGCAGGGTTGACTC
ACTGAATGCCAATAATCATAAATATTCAACTAAACCCTAAAATAGCGAAAGCTCAATTTGTTTCTATATTT
ATCGGAGTAAACATGACATTTTTCCCCCAACACTTTTTAGGATTAAATGGA
>DspR88_1
AACCATGTATTTGGTTTtTGGAGGATGAGCTgGGATTTTTGGAGGGTCTCTAAGAGTTATTATTcGTCTTG
AATTAGGACAGCCAGGTAGATTAATAGGGAATGACCAGATcTATAATACAATCGTTACAGCACACGCATTT
GTTATAATTTTTTTTATAGTAATACCAATAATAATTGGGGGATTTGGAAATTGATTGGTTCCTTTATTTTT
AGGAAGACAAGACATAGCATTTCCACGAATAAATAACATAAGATTTTGACTGCTTCCCCCATCTCTAATTT
TACTTTTATTATCTTCAATTTCAGGAAGGGGTGTTGGGACTGGGTGAACTGTGTACCCCCCTCTATCAGAT
AGAATGTTTCATTACGGAAGATCCGTAGATTTGGCAATCTTTAGGTTACACATTGCAGGAATTTCATCAAT

TTTAGGATCAATTAATTTCATCGCTACCGTAATTAATATACGAATACCATCTTTATCTATAGAAAACATTC
CTTTATTTGTATGATCTGTTGTAATTACAACAGTACTTTTACTTCTATCTCTCCCTGTACTGGCAGGAGGA
ATTACCATATTACTAACAGACCGTAACTTTAATACAACCTTCTTTGATCCTTCGGGAGGAGGGGAtCCAAT
CCTATACCAACATTTATtttGaTTTTttGGaCACCCTGAaGTATATATTCTAATTCTACCAGGTTTTGGAG
TAATTTCACATGTAATTAGATTCTATTCAAACAAAAAAGAACCATTTGGAAGTATAGGAATAATCTACGCT
ATACTATCAATTGGATTTTTAGGTTTTATTGTATGAGCCCACCATATATTTACTGTAGGAATAGATGTAGA
CACACGAGCTTACTTTACaGCAGCTACTATAATTATTGCTATTCCGACAGGAGTAAAAGTATTCAGCTGAC
TGGCAACAATAAACAGATCTTACCTAATAATAGAAGCTCCAATAATATGAGCCTTAGGTTTCATTGTTCTT
TTTACCTTAGGAGGTTTAACAGGGATTGTCTTATCAAATTCTTCAATTGATATCACTCTTCATGATACTTA
CTACGTAGTAGCTCATTTTCATTATGTCCTTTCTATAGGAGCAGTTTTTGCATTAATAGCAGGGTTGACTC
ACTGAATGCCAATAATCATAAATATTCAACTAAACCCTAAAATAGCGAAAGCTCAATTTGTTTCTATATTT
ATCGGAGTAAACATGACATTTTTCCCCCaacaCTTTTTAGGATTAAATGGA
>DspR88_2
AACCATGTATTTgGTTtTTGGagGATGAGCTGGGAtTTTtGGAGGGTCTCTAAGAGTTATTaTTCGTCTTG
AATTAGGACAGCCAGGTAGATTAATAGGGAATGACCAGATCTATAATACAATCGTTACAGCACACGCATTT
GTTATAATTTTTTTTATAGTAATACCaATAATAATTGGGGGATTTGGAAATTGATTGGTTCCTTTATTTTT
AGGAAGACAAGACATAGCATTTCCACGAATAAATAACATAAGATTTTGACTGCTTCCCCCATCTCTAATTT
TACTTTTATTATCTTCAATTTCAGGAAGGGGTGTTGGGACTGGGTGAACTGTGTACCCCCCTCTATCAGAT
AGAATGTTTCATTACGGAAGATCCGTAGATTTGGCAATCTTTAGGTTACACATTGCAGGAATTTCATCAAT
TTTAGGATCAATTAATTTCATCGCTACCGTAATTAATATACGAATACCATCTTTATCTATAGAAAACATTC
CTTTATTTGTATGATCTGTTGTAATTACAACAGTACTTTTACTTCTATCTCTCCCTGTACTGGCAGGAGGA
ATTACCATATTACTAACAGACCGTAACTTTAATACaACcTTCTTTGATCCTTCGGGAGGAGGGGATCCAAT
CCTATACCAAcATTTaTtTtgattTTTTGGaCACCCTGAAGTATATATTCTAATTCTACCAGGTTTTGGAG
TAATTTCACATGTAATTAGATTCTATTCAAACAAAAAAGAACCATTTGGAAGTATAGGAATAATCTACGCT
ATACTATCAATTGGATTTTTAGGTTTTATTGTATGAGCCCACCATATATTTACTGTAGGAATAGATGTAGA
CACACGAGCTTACTTTACAGCAGCTACTATAATTATTGCTATTCCGACAGGAGTAAAAGTATTCAGCTGAC
TGGCAACAATAAACAGATCTTACCTAATAATAGAAGCTCCAATAATATGAGCCTTAGGTTTCATTGTTCTT
TTTACCTTAGGAGGTTTAACAGGGATTGTCTTATCAAATTCTTCAATTGATATCACTCTTCATGATACTTA
CTACGTAGTAGCTCATTTTCATTATGTCCTTTCTATAGGAGCAGTTTTTGCATTAATAGCAGGGTTGACTC
ACTGAATGCCAATAATCATAAATATTCAACTAAACCCTAAAATAGCGAAAGCTCAATTTGTTTCTATATTT
ATCGGAGTAAACATGACATTTTTCCCCCAACACtttttaggAttAAAtGGA
>DspR88_3
AACCATGTaTttGGTTTTTgGAgGATGAGctGGGATTTTTGGAgGGTcTcTAAGAGTTATTATTCGTCTTG
AATTAGGACAGCCAGGTAGATTAATAGGGAATGACCAGATCTATAATACAATCGTTACAGCACACGCATTT
GTTATAATTTTTTTTATAGTAATACCAATAATAATTGGGGGATTTGGAAATTGATTGGTTCCTTTATTTTT
AGGAAGACAAGACATAGCATTTCCACGAATAAATAACATAAGATTTTGACTGCTTCCCCCATCTCTAATTT
TACTTTTATTATCTTCAATTTCAGGAAGGGGTGTTGGGACTGGGTGAACTGTGTACCCCCCTCTATCAGAT
AGAATGTTTCATTACGGAAGATCCGTAGATTTGGCAATCTTTAGGTTACACATTGCAGGAATTTCATCAAT
TTTAGGATCAATTAATTTCATCGCTACCGTAATTAATATACGAATACCATCTTTATCTATAGAAAACATTC
CTTTATTTGTATGATCTGTTGTAATTACAACAGTACTTTTACTTCTATCTCTCCCTGTACTGGCAGGAGGA
ATTACCATATTACTAACAGACCGTAACTTTAATACaacCTTCTTTGATCCTTCGGGAGGAGGGGATCCAAT
CCTATACCAACATTTAttttgATTTTTTGGACaCCCtGAAGTATATATTcTAATtcTACCaGGtTTTGGAG
TAATTTCACAtGTAATTAGATTcTATTCAAACAAAAAAGAACCATTTGGAAGTATAGGAATAATCTACGCT
ATACTATCAATTGGATTTTTAGGTTTTATTGTATGAGCCCACCATATATTTACTGTAGGAATAGATGTAGA
CACACGAGCTTACTTTACAGCAGCTACTATAATTATTGCTATTCCGACAGGAGTAAAAGTATTCAGCTGAC
TGGCAACAATAAACAGATCTTACCTAATAATAGAAGCTCCAATAATATGAGCCTTAGGTTTCATTGTTCTT
TTTACCTTAGGAGGTTTAACAGGGATTGTCTTATCAAATTCTTCAATTGATATCACTCTTCATGATACTTA
CTACGTAGTAGCTCATTTTCATTATGTCCTTTCTATAGGAGCAGTTTTTGCATTAATAGCAGGGTTGACTC
ACTGAATGCCAATAATCATAAATAttCAACTAAACCCTAAAATAGCGAAAGCTCAATTTGTTTCTATATTT
ATCGGAGTAAACATGACATTTTTCCCCCAACACtttttaggAttAAAtGGA

***18S rRNA* alignment of 56 oribatid mite species used for phylogenetic analyses in the present study.** Of the 56 sequences, 54 were obtained from GenBank (see Appendix Table) and two (DdR2_3 and Spin_sp) generated in this work (see also Table 1 in the paper). Sequences were aligned using the R-Coffee web server and the program Gblocks v0.91b was applied to eliminate poorly aligned positions/regions. The final *18S* alignment had a total length of 1,375 bp.

```
>Aeroppia_sp
ATCAGTTACG GTTAGATGTT GACGTTTACA TGGATAACTG TGGTAATTCT AGAGCTAATA
CATGCCGAAA AACTTCAACC GGGGAGAAGT GCATTTATTA GAACAAGACC AATGGGGGTG
GTGACTCTGG ATAACTGCTA ATCGTATGGC CGTGCCGGCG ATGAATTCAA GTGTCTGCCT
TATCAACTGT CGATGGTAGG TTATGCGCCT ACCATGGTTG TAACGGGTAA CGGGGAATCA
TTCGATTCCA GCCTGAGAAA CTAGCACATC CAAGGAAGGC AGCAGGCGTG CAAATTACCC
AAGGTAGTGA CGAAAAATAA CAATACGAGA CTCTTATGAG GCCTCGTAAT TGGAATGAGA
ACAATCTAAA AATTTAACGA GGATCCATTG GAGGGCAAGT CTGGTGCCAG CAGCCGCGGT
AATCCAGCTC CAATAGCGTA TATTAAAGTT GTTGTGGTTA AAAAGCTCGT AGTTGGATCT
CAGTTCTAGT CATCGGTCCA CTTGCCAGTG GTTACTGTTT TGAACATTAC CGCCTATGGT
GCTCTTCACC GAGTGTCATA GGCGATCGGT ACGTTTACTT TGAAAAAATT AGAGTGCTCA
AAGCAGGCGC CCGAATAATG TTGCATGGAA TAATAGAATA GGACCTCGGT TCTATTTTGT
TGGTCTTCGG GAACGAGGTA ATGATCATAG GGACAGACGG GGCTATTCGT ATTGCGGAGC
TAGAGGTGAA ATTCTTGGAC CTTCGCAAGA CGAACTAAAG CGAAAGCAGC AAGAATGTTT
TCATTAATCA AGAACGAAAG TTAGAGGTTC GAAGGCGATC AGATACCGCC CTAGTTCTAA
CCATAAACGA TGCCGACCAG TAATTCGCCT GAGTTCAAAT GACTCGGGGC TTCCGGGAAA
CCAAAGTTCG GTTCCAGGGG AAGTATGGTT GCAAAGCTGA AACTTAAAGA AATTGACGGA
AGGGCACCAC CAGGAGTGGA GCCTGCGGCT TAATTTGACT CAACACGGGA AAACTCACCC
GGCCCGGACA CTGGAAGGAT TGACAGATTG AGAGCTCTTT CTTGATTCAG TGGGTGGTGG
TGCATGGCCG TTCTTAGTTG GTGGAGTGAT TTGTCTGGTT AATTCCGATA ACGAACGAGA
CTCTAGCCTA CTAACTAACC TCGTCTTCTT AGAGGGACAG GCGGTGTTTC AACCGCACGA
AACAGAGCAA TAACAGGTCT GTGATGCCCT TAGATGTCCG GGGCCGCACG CGCGCTACAC
TGAAGTGATC AGCGTGCATC CTACTCTGTC AAGAGTGGGG AACCGATTGA ACCTTCGTGA
TTGGGATATT GTAATTATTC TCCTTGAACG AGGAATTCCC AGTAATCGCG AGTCA
>Adoristes_poppei
ATCAGTTACG GTTAGATGTT GACATCTACA TGGATAACTG TGGTAATTCT AGAGCTAATA
CATGCACAAA AGCTTCGACC TGGAAGAAGC GCATTTATTA GACCAAGACC AATGGGGGTG
GTGACTCTGT ATAACTGCTA ATCGCATGGC CGTGCCGGCG ATGAATTCAA GTGTCTGCCT
TATCAACTGT CGATGGTAGG CTATGCGCCT ACCATGGTTG TAACGGGTAA CGGGGAATCA
TTCGATTCCA GCCTGAGAAA CTACCACATC CAAGGAAGGC AGCAGGCACG CAAATTACCC
AAGGTAGTGA CGAAAAATAA CAATACGGGA CTCTTATGAG GCCCCGTAAT TGGAATGAGA
ACAATCTAAA TCCTTAACGA GGATCTATTG GAGGGCAAGT CTGGTGCCAG CAGCCGCGGT
AATCCAGCTC CAATAGCGTA TATTAAAGTT GTTGTGGTTA AAAAGCTCGT AGTTGGATCT
CAGTTCGAGT CGGTGGTCCA CTTGCCAGTG GTTACTACTT TGAACATTAT CGCCTATGGT
GCTCTTCACC GAGTGTCATA GGCGATCGAT ACGTTTACTT TGAAAAAATT AGAGTGCTCA
AAGCAGGCGC CCGAATAATG TTGCATGGAA TAATGGAATA GGACCTCGGT TCTATTTTGT
TGGTCTTCGG AACTGAGGTA ATGATTAGAG GGACAGACGG GGGCATTCGT ATTGCGGCGC
TAGAGGTGAA ATTCTTGGAC CGTCGCAAGA CGAACTAAAG CGAAAGCACC AAGAATGTTT
TCATTAATCA AGAACGAAAG TTAGAGGTTC GAAGGCGATC AGATACCGCC CTAGTTCTAA
CCATAAACGA TGCCGACCAG TAATTTAGCT GAGTTCAAAT GACTCGCGAC TTCCGGGAAA
CCAAAGTTCG GTTCCAGGGG AAGTATGGTT GCAAAGCTGA AACTTAAAGA AATTGACGGA
AGGGCACCAC CAGGAGTGGA GCCTGCGGCT TAATTTGACT CAACACGGGA AAACTCACCC
GGCCCGGACA CTGGAAGGAT TGACAGATTG AGAGCTCTTT CTTGATTCAG TGGGTGGTGG
TGCATGGCCG TTCTTAGTTG GTGGAGTGAT TTGTCTGGTT AATTCCGATA ACGAACGAGA
CTCTAGCCTA CTAAATAACG CTAACTTCTT AGAGGGACAG GCGGTGATTC AACCGCACGA
AACAGAGCAA TAACAGGTCT GTGATGCCCT TAGATGTCCG GGGCCGCACG CGCGCTACAC
TGAAGTGATC AGCGTGCAGC CTACTCTGTC AAGAGTGGGA AACCCAATGA ACCTTCGTGA
TTGGGATATT GTAATTATTC CCCTTGAACG AGGAATTCCC AGTAAGCGCG AGTCA
>Palaeacarus_hystricinus
ATCAGTCCTG AGTTGAATTT GAAAGTTACA TGGATAACTG TGGTAATTCT AGAGCTAATA
CATGCACAAA TGCTTCGACC CGGAAGAAGC GCGTTTATTA GACCAAGACC AATGGAGGTG
GTGACTCTGG ATAACTGCAG ATCGCATGGC CGTGCCGGCG ACAAATTCAC GGGTCTGCCT
```

TATCAACTGT CGATGGTAGG TTATATGCCT ACCATGGTTG TAACGGGTAA CGGGGAATCA
TTCGATTCCA GCCTGAGAAA CTACCACATC CAAGGAAGGC AGCAGGCACG CAAATTACCC
AAGGTAGTGA CGAAAAATAA CAATACAGCA CTCTAATGAG GCGCTGTAAT TGGAATGAGA
ACAATTTAAA TCCTTAACGA GGATCCATTG GAGGGCAAGT CTGGTGCCAG CAGCCGCGGT
AATCCAGCTC CAATAGCGTA TATTAAAGTT GTTGCGGTTA AAAAGCTCGT AGTTGGATCT
CAGTTCGTGC CGGCGGTCCA CTTGCCAGTG GCTACTTCCA CGAACATTAC CGCTCATGGT
GCCCTTAACC GAGTGTCATG AGCGATCGGT ACGTTTACTT TGAAAAAATT AGAGTGCTTA
AAGCAGGCGC CCGAATAATG TTGCATGGAA TAATGGAATA TGTAGGTAAC TCTGTTCTAT
TGGTCTTCGG AGTTTCCTAA ATGGTTACAG GGACAGACGG GGGCGTTCGT ATTGCGGCGC
TAGAGGTGAA ATTCTTGGAC CGTCGCAAGA CGAACTAAAG CGATAGCACC AAGAATGTTT
TCATTAATCA AGAACGAAAG TTAGAGGTTC GAAGGCGATC AGATACCGCC CTAGTTCTAA
CCATAAACGA TGCCAACCAG CGATTCGCCT GAGTTCAAAT GACTCGCAGC TTCCGTGAAA
ACAAAGTTCG GTTCCGGGGG AAGTATGGTT GCAAAGCTGA AACTTAAAGG AATTGACGGA
GAGGCACCAC CAGGAGTGGA GCCTGCGGCT TAATTTGACT CAACACGGGG AAACTCACCC
GGCCCGGACA CTGAAAGGAT TGACAGATTG AGAGCTCTTT CTTGATTCAG TGGGTGGTGG
TGCATGGCCG TTCTTAGTTG GTGGAGCGAT TTGTCTGGTT AATTCCGATA ACGAACGAGA
CTCTAGCCTA TTAAATAACG CCGACTTCTT AGAGGGACAG GCGGCGATTC AGCCGCACGA
AACAGAGCAA TAACAGGTCT GTGATGCCCT TAGATGTCCG GGGCCGCACG CGCGCTACAC
TGAAGTGATC AGCGTGCCTC CTGCTCTGTC AAGAGTGGGT AACCCAATGA ACCTTCGTGA
TTGGGATATT GTAATTATTC TCCTTGAACG AGGAATTCCC AGTAAGCGCG AGTCA
>Hermannia_gibba
ATCAGTTACG GTTAGATGTT GACAACTACA TGGATAACTG TGGTAATTCT AGAGCTAATA
CATGCACAAA AGCTTCGACC TGGAAGAAGC GCATTTATTA GACCAAGACC AATGGGGGTG
GTGACTCTGG ATAACTGCTA ATCGTATGGC CGTGCCGACG ATGAATTCAA GTGTCTGACT
TATCAACTGT CGATGGTAGG CTATGCGCCT ACCATGGTTG TAACGGGTAA CGGGGAATCA
TTCGATTCCA GCCTGAGAAA CTACCACATC CAAGGAAGGC AGCAGGCACG CAAATTACCC
AAGGTAGTGA CGAAAAATAA CAATACGGGA CTCTTATGAG GCCCCGTAAT TGGAATGAGA
ACAATCTAAA TCCTTAACGA GGATCTATTG GAGGGCAAGT CTGGTGCCAG CAGCCGCGGT
AATCCAGCTC CAATAGCGTA TATTAAAGTT GTTGTGGTTA AAAAGCTCGT ACTTGGATCT
CAGTTCAAGT TGACGGTCCA CTTGCCAGTG GCTACTGTCT TGAACATTAC CGCTTATGGT
GCTCTTCACC GAGTGTCATA AGCGATCGGT ACGTTTACTT TGAAAAAATT AGAGTGCTCA
AAGCAGGCGC CCGAATAATG TTGCATGGAA TAATGGAATA GGACCTCGGT TCTATTTTGT
TGGTCTTCGG AACTGAGGTA ATGATTAGAG GGACAGACGG GGGCATTCGT ATTGCGGCGC
TAGAGGTGAA ATTCTTGGAC CGTCGCAAGA CGAACTAAAG CGAAAGCACC AAGAATGTTT
TCATTAATCA AGAACGAAAG TTAGAGGTTC GAAGGCGATC AGATACCGCC CTAGTTCTAA
CCATAAACGA TGCCAACCAG CAATCCGTCT GAGTTCAAAT GACTCGCGGC TTCCGGGAAA
CCAAAGTTCG GTTCCAGGGG AAGTATGGTT GCAAAGCTGA AACTTAAAGG AATTGACGGA
AGGGCACCAC CAGGAGTGGA GCCTGCGGCT TAATTTGACT CAACACGGGA AAACTCACCC
GGCCCGGACA CTGGAAGGAT TGACAGATTG AGAGCTCTTT CTTGATTCAG TGGGTGGTGG
TGCATGGCCG TTCTTAGTTG GTGGAGCGAT TTGTCTGGTT AATTCCGATA ACGAACGAGA
CTCTAGCCTA CTAAATAACT TATACTTCTT AGAGGGACAG GCGGCGATTC AGCCGCACGA
AACAGAGCAA TAACAGGTCT GTGATGCCCT TAGATGTCCG GGGCCGCACG CGCGCTACAC
TGAAGTGATC AGCGTGCTTC CTGCTCAGAC ATGAGTGGGA AACCCAATGA ACCTTCGTGA
TAGGGACTTT GTAATTATTC TCCACGAACG AGGAATTCCC AGTAAGCGCG AGTCA
>Nothrus_silvestris
ATCAGTTACG GTTAGATGTT GACATCTACA TGGATAACTG TGGTAATTCT AGAGCTAATA
CATGCACAAA AGCTTCGACC TGGAAGAAGC GCATTTATTA GACCAAGACC AATGGGGGTG
GTGACTCTGG ATAACTGCTA ATCGCATGGC CGTGCCGGCG ATGAATTCAA GTGTCTGCCT
TATCAACTGT CGATGGTAGG TTATGCGCCT ACCATGGTTG TAACGGGTAA CGGGGAATCA
TTCGATTCCA GCCTGAGAAA CTACCACATC CAAGGAAGGC AGCAGGCACG CAAATTACCC
AAGGTAGTGA CGAAAAATAA CAATACGGGA CTCTTATGAG GCCCCGTAAT TGGAATGAGA
ACAATCTAAA TCCTTAACGA GGATCTATTG GAGGGCAAGT CTGGTGCCAG CAGCCGCGGT
AATCCAGCTC CAATAGCGTA TATTAAAGTT GTTGTGGTTA AAAAGCTCGT AGTTGGATCT
CAGTTCGAGT CGGCGGTCCA CTTGCCAGTG GTTACTGCTT TGAACATTAT CGCCTATAGT
GCTCTTCACC GAGTGTTATA GGCGATCGGT ACGTTTACTT TGAAAAAATT AGAGTGCTCA
AAGCAGGCGC CCGAATAATG TTGCATGGAA TAATGGAATA GGACCTCGGT TCTATTTTGT
TGGTCTTCGG AACTGAGGTA ATGATTAGAG GGACAGACGG GGGCATTCGT ATTGCGGCGC
TAGAGGTGAA ATTCTTGGAC CGTCGCAAGA CGAACTAAAG CGAAAGCACC AAGAATGTTT
TCATTAATCA AGAACGAAAG TTAGAGGTTC GAAGGCGATC AGATACCGCC CTAGTTCTAA
CCATAAACGA TGCCAACCAG TAATCCGTCT GAGTTCAAAT GACTCGCGAC TTCCGGGAAA

CCAAAGTTCG GTTCCAGGGG AAGTATGGTT GCAAAGCTGA AACTTAAAGG AATTGACGGA
AGGGCACCAC CAGGAGTGGA GCCTGCGGCT TAATTTGACT CAACACGGGA AAACTCACCC
GGCCCGGACA CTGGAAGGAT TGACAGATTG AGAGCTCTTT CTTGATTCAG TGGGTGGTGG
TGCATGGCCG TTCTTAGTTG GTGGAGCGAT TTGTCTGGTT AATTCCGATA ACGAACGAGA
CTCTAGCCTA CTAAATAACG CCGACTTCTT AGAGGGACAG GCGGCGATTC AGCCGCACGA
AACAGAGCAA TAACAGGTCT GTGATGCCCT TAGATGTCCG GGGCCGCACG CGCGCTACAC
TGAAGTGATC AGCGTGCAGC CTACTCTGCC AAGAGTGGGA AACCCAATGA ACCTTCGTGA
TTGGGATATT GTAATTATTC CCCTTGAACG AGGAATTCCC AGTAAGCGCG AGTCA
>Eniochthonius_minutissimus
ATCAGTTACG GTTAGATGTT GACGTCTACA TGGATAACTG TGGTAATTCT AGAGCTAATA
CATGCACAAA AGCTTCGACC TGGAAGAAGC GCATTTATTA GACCAAGACC AATGGGGGTG
ATGAATCTGA ATAACTGCTA ATCGCATGGC CGCGCCGGCG ATGAATTCAA GTGTCTGCCT
TATCAACTGT CGATGGTAGG TTATGCGCCT ACCATGGTTG TAACGGGTAA CGGGGAATCA
TTCGATTCCA GCCTGAGAAA CTACCACATC CAAGGAAGGC AGCAGGCACG CAAATTACCC
ACGGTAGTGA CGAAAAATAA CAATACGGGA CTCTAATGAG GCCCCGTAAT TGGAATGAGA
ACAATCTAAA TCCTTAACGA GGATCTATTG GAGGGCAAGT CTGGTGCCAG CAGCCGCGGT
AATCCAGCTC CAATAGCGTA TATTAAAGTT GTTGCGGTTA AAAAGCTCGT AGTTGGATCT
CAGTTCGCGT CGGCGGTCCA CTTGCCAGTG GTTACTGCCT TGAACATTAC CGCCCATGGT
GCTCTTCGCC GAGTGTCATG GGCGATCGGT ACGTTTACTT TGAAAAAATT AGAGTGCTCA
AAGCAGGCGC CCGAATAATG TTGCATGGAA TAATGGAATA GGACCTCGGT TCTATTTTGT
TGGTCTTCGG AACTGAGGTA ATGATTAGAG GGACAGACGG GGGCATTCGT ATTGCGGCGC
TAGAGGTGAA ATTCTTGGAC CGTCGCAAGA CGAACTAAAG CGAAAGCACC AAGAATGTTT
TCATTAATCA AGAACGAAAG TTAGAGGTTC GAAGGCGATC AGATACCGCC CTAGTTCTAA
CCATAAACGA TGCCAACCAG CAATCCGCCT GAGTTCAAAT GACTCGCGGC TTCCGGGAAA
CCAAAGTTCG GTTCCGGGGG AAGTATGGTT GCAAAGCTGA AACTTAAAGG AATTGACGGA
AGGGCACCAC CAGGAGTGGA GCCTGCGGCT TAATTTGACT CAACACGGGA AAACTTACCC
GGCCCGGACA CTGGAAGGAT TGACAGATTG AGAGCTCTTT CTTGATTCAG TGGGTGGTGG
TGCATGGCCG TTCTTAGTTG GTGGAGCGAT TTGTCTGGTT AATTCCGATA ACGAACGAGA
CTCTAGCCTA CTAAATAACG TCGGCTTCTT AGAGGGACAG GCGGCGATTC AGCCGCACGA
AACAGAGCAA TAACAGGTCT GTGATGCCCT TAGATGTCCG GGGCCGCACG CGCGCTACAC
TGAAGTGATC AGCGTGCCGC CTGCTCTGCC AAGAGTGGGT AACCCAATGA ACCTTCGTGA
TTGGGATATT GTAATTATTC TCCTTGAACG AGGAATTCCC AGTAAGCGCG AGTCA
>Tectocepheus_velatus
ATCAGTTACG GTTAGATGTT GACATCTACA TGGATAACTG TGGTAATTCT AGAGCTAATA
CATGCACAAA AGCTTCGACC TGGAAGAAGC GCATTTATTA GACCAAGACC AATGGGGGTG
GTGACTCTGG ATAACTGCTA ATCGCATGGC CGAGCCGGCG ATGAATTCAA GTGTCTGCCT
TATCAACTGT CGATGGTAGG TTATGTGCCT ACCATGGTTG TAACGGGTAA CGGGGAATCA
TTCGATTCCA GCCTGAGAAA CTACCACATC CAAGGAAGGC AGCAGGCACG CAAATTACCC
AAGGTAGTGA CGAAAAATAA CAATATGGAA CTCTTATGAG GCTCCATAAT TGGAATGAGA
ACAATCTAAA TCCTTAACGA GGATCTATTG GAGGGCAAGT CTGGTGCCAG CAGCCGCGGT
AATCCAGCTC CAATAGCGTA TATTAAAGTT GTTGTGGTTA AAAAGCTCGT AGTTGGATCT
CAGTTCGAGT CGGCGGTCCA CTTGCCAGTG GTTACTGTTT TGAACATTAT CGCCTATGGT
GCTCTTCACC GAGTGTCATA GGCGATCGAT ACGTTTACTT TGAAAAAATT AGAGTGCTCA
AAGCAGGCGC CCGAATAATG TTGCATGGAA TAATGGAATA GGACCTCGGT TCTATTTTGT
TGGTCTTCGG AACTGAGGTA ATGATTAGAG GGACAGACGG GGGCATTCGT ATTGCGACGC
TAGAGGTGAA ATTCTTGGAC CGTTGCAAGA CGAACTAAAG CGAAGGCACC AAGAATGTTT
TCATTAATCA AGAACGAAAG TTAGCGGATC GAAGGCGATC AGATACCGCC CTAGTGCTAA
CCATAAACTA TGCCAACCAG TAATAAGCCT GAGTTCAAAT GACTCGGGAC TTCCGAGAAA
TCAAAGTTCG GTTCTAGGGG AAGTATGGTT GCAAAGCTGA AACTTAAAGA AATTGACGGA
AGGGCACCAC CAGGAGTGGA GCCTGCGGCT TAATTTGACT CAACACGGGA AAACTCACCC
GGCCCGGACA CTGGAAGGAT TGACAGATTG AGAGCTCTTT CTTGATTCAG TGGGTGGTGG
TGCATGGCCG TTCTTAGTTG GTGGAGTGAT TTGTCTGGTT AATTCCGATA ACGAACGAGA
CTCTAGCCTA CTAAATAACG CCGACTTCTT AGAGGGACAG GCGGTGATTC AACCGCACGA
AACAGAGCAA TAACAGGTCT GTGATGCCCT TAGATGTCCG GGGCCGCACG CGCGCTACAC
TGAAGTGATC AGCGTGCAGC CTACTCTGTC AAGAGTGGGA AACCCAATGA ACCTTCGTGA
TTGGGATATT GTAATTATTC CCCTTGAACG AGGAATTCCC AGTAAGCGCG AGTCA
>Tectocepheus_sarekensis
ATCAGTTACG GTTAGATGTT GACATCTACA TGGATAACTG TGGTAATTCT AGAGCTAATA
CATGCACAAA AGCTTCGACC TGGAAGAAGC GCATTTATTA GACCAAGACC AATGGGGGTG
GTGACTCTGG ATAACTGCTA ATCGCATGGC CGAGCCGGCG ATGAATTCAA GTGTCTGCCT

TATCAACTGT CGATGGTAGG TTATGTGCCT ACCATGGTTG TAACGGGTAA CGGGGAATCA
TTCGATTCCA GCCTGAGAAA CTACCACATC CAAGGAAGGC AGCAGGCACG CAAATTACCC
AAGGTAGTGA CGAAAAATAA CAATACTGGA CTCTTATGAG GCCCAGTAAT TGGAATGAGA
ACAATCTAAA TCCTTAACGA GGATCTATTG GAGGGCAAGT CTGGTGCCAG CAGCCGCGGT
AATCCAGCTC CAATAGCGTA TATTAAAGTT GTTGTGGTTA AAAAGCTCGT AGTTGGATCT
CAGTTCGAGT CGACGGTCCA CTTGCCAGTG GTTACTGTTT TGAACATTAT CGCCTATGGT
GCTCTTCACC GAGTGTCAGA GGCGATCGAT ACGTTTACTT TGAAAAAATT AGAGTGCTCA
AAGCAGGCGC CCGAATAATG TTGCATGGAA TAATGGAATA GGACCTCGGT TCTATTTTGT
TGGTCTTCGG AACTGAGGTA ATGATTAGAG GGACAGACGG GGGCATTCGT ATTGCGACGC
TAGAGGTGAA ATTCTTGGAC CGTTGCAAGA CGAACAAAAG CGAAGGCACC AAGAATGTTT
TCATTAATCA AGAACGAAAG TTAGCGGATC GAAGGCGATC AGATACCGCC CTAGTGCTAA
CCATAAACGA TGCCAACCAG TAATAAGCCT GAGTTCAAAT GACTCGGGAC TTCCGAGAAA
TCAAAGTTCG GTTCCAGGGG AAGTATGGTT GCAAAGCTGA AACTTAAAGA AATTGACGGA
AGGGCACCAC CAGGAGTGGA GCCTGCGGCT TAATTTGACT CAACACGGGA AAACTCACCC
GGCCCGGACA CTGGAAGGAT TGACAGATTG AGAGCTCTTT CTTGATTCAG TGGGTGGTGG
TGCATGGCCG TTCTTAGTTG GTGGAGTGAT TTGTCTGGTT AATTCCGATA ACGAACGAGA
CTCTAGCCTA CTAAATAACG CCGACTTCTT AGAGGGACAG GCGGTGATTC AACCGCACGA
AACAGAGCAA TAACAGGTCT GTGATGCCCT TAGATGTCCG GGGCCGCACG CGCGCTACAC
TGAAGTGATC AGCGTGCAGC CTACTCTGTC AAGAGTGGGA AACCCAATGA ACCTTCGTGA
TTGGGATATT GTAATTATTC CCCTTGAACG AGGAATTCCC AGTAAGCGCG AGTCA
>Tectocepheus_minor
ATCAGTTACG GTTAGATGTT GACATCTACA TGGATAACTG TGGTAATTCT AGAGCTAATA
CATGCACAAA AGCTTCGACC TGGAAGAAGC GCATTTATTA GACCAAGACC AATGGGGGTG
GTGACTCTGG ATAACTGCTA ATCGCATGGC CGAGCCGGCG ATGAATTCAA GTGTCTGCCT
TATCAACTGT CGATGGTAGG TTATGTGCCT ACCATGGTTG TAACGGGTAA CGGGGAATCA
TTCGATTCCA GCCTGAGAAA CTACCACATC CAAGGAAGGC AGCAGGCACG CAAATTACCC
AAGGTAGTGA CGAAAAATAA CAATATGGAA CTCTTATGAG GCTCCATAAT TGGAATGAGA
ACAATCTAAA TCCTTAACGA GGATCTATTG GAGGGCAAGT CTGGTGCCAG CAGCCGCGGT
AATCCAGCTC CAATAGCGTA TATTAAAGTT GTTGTGGTTA AAAAGCTCGT AGTTGGATCT
CAGTTCGAGT CGGCGGTCCA CTTGCCAGTG GTTACTGTTT TGAACATTAT CGCCTATGGT
GCTCTTCACC GAGTGTCATA CGCGATCGAT ACGTTTACTT TGAAAAAATT AGAGTGCTCA
AAGCAGGCGC CCGAATAATG TTGCATGGAA TAATGGAATA GGACCTCGGT TCTATTTTGT
TGGTCTTCGG AACTGAGGTA ATGATTAGAG GGACAGACGG GGGCATTCGT ATTGCGACGC
TAGAGGTGAA ATTCTTGGAC CGTTGCAAGA CGAACTAAAG CGAAGGCACC AAGAATGTTT
TCATTAATCA AGAACGAAAG TTAGCGGATC GAAGGCGATC AGATACCGCC CTAGTGCTAA
CCATAAACTA TGCCAACCAG TAATAAGCCT GAGTTCAAAT GACTCGGGAC TTCCGAGAAA
TCAAAGTTCG GTTCTAGGGG AAGTATGGTT GCAAAGCTGA AACTTAAAGA AATTGACGGA
AGGGCACCAC CAGGAGTGGA GCCTGCGGCT TAATTTGACT CAACACGGGA AAACTCACCC
GGCCCGGACA CTGGAAGGAT TGACAGATTG AGAGCTCTTT CTTGATTCAG TGGGTGGTGG
TGCATGGCCG TTCTTAGTTG GTGGAGTGAT TTGTCTGGTT AATTCCGATA ACGAACGAGA
CTCTAGCCTA CTAAATAACG CCGACTTCTT AGAGGGACAG GCGGTGATTC AACCGCACGA
AACAGAGCAA TAACAGGTCT GTGATGCCCT TAGATGTCCG GGGCCGCACG CGCGCTACAC
TGAAGTGATC AGCGTGCAGC CTACTCTGTC AAGAGTGGGA AACCCAATGA ACCTTCGTGA
TTGGGATATT GTAATTATTC CCCTTGAACG AGGAATTCCC AGTAAGCGCG AGTCA
>Thalassozetes_shimojanai
ATCAGTTACG GTTAGATGTT GACATCTACA TGGATAACTG TGGTAATTCT AGAGCTAATA
CATGCACAAA AGCTTCGACC TGGAAGAGGC GCATTTATTA GACCAAGACC AATGGGGGTG
GTGACTCTGG ATAACTGCTA ATCGCATGGC CGAGCCGGCG ATGAATTCAA GTGTCTGCCT
TATCAACTGT CGATGGTAGG TTATGCGCCT ACCATGGTTG TAACGGGTAA CGGGGAATCA
TTCGGTTCCA GCCTTAAAAA CTACCACATC CAAGGAAGGC AGCAGGCACG CAAATTACCC
AAGGTAGTGA CGAAAAATAA CAATACGAGA CTCTTATGAG GCCTCGTAAT TGGAATGAGA
ACAATTTAAA TCCTTAACGA GGATCTATTG GAGGGCAAGT CTGGTGCCAG CAGCCGCGGT
AATCCAGCTC CAATAGCGTA TATTAAAGTT GTTGTTGTTA AAAAGCTCGT AGTTGGATCT
CAGTTCTAGT TGATGGTCCA CTTTGAAGTG GCTACTGTCT TGAACAGTAT CGCCTATGGT
GCTCTTCACC GAGTGTCATA GGCGATCGAT AGGTTTACTT TGAAAAAATT AGAGTGCTCA
AAGCAGGCGC CCGAATAATG TTGCATGGAA TAATGGAATA GGACCTCGGT TCTATTTTGT
TGGTCTTCGG AACTGAGGTA ATGATTAGAG GGACAGACGG GGGCATTCGT ATTGCAGCGC
TAGAGGTGAA ATTCTTGGAC CGCTGCAAGA CGAACTAAAG CGAAAGCACC AAGAATGTTT
TCATTAATCA AGAACGAAAG TTAGAGGTTC GAAGGCGATC AGATACCGCC CTAGTTCTAA
CCATAAACGA TGCCAACCAG TAATAGGTCT GAGTTCAAAT GACTCGCGAC TTCCGGGAAA

CCAAAGTTCG GTTCAAGGGG AAGTATGGTT GCAAAGCTGA AACTTAAAGA AATTGACGGA
AGGGCACCAC CAGGAGTGGA GCCTGCGGCT TAATTTGACT CAACACGGGA AAACTCACCC
GGCCCGGACA CTGGAAGGAT TGACAGATTG AGAGCTCTTT CTTGATTCAG TGGGTGGTGG
TGCATGGCCG TTCTTAGTTG GTGGAGTGAT TTGTCTGGTT AATTCCGATA ACGAACGAGA
CTCTAGCCTA CTAAATAACG TCAACTTCTT AGAGGGACAG GCGGTGATTC AACCGCACGA
AACAGAGCAA TAACAGGTCT GTGATGCCCT TAGATGTCCG GGGCCGCACG CGCGCTACAC
TGAAGTGATC AGCGTGCGGC CTACTCTGTC AAGAGTGGGA AACCCAATGA ACCTTCGTGA
TTGGGATATT GTAATTATTG CCCTTGAACG AGGAATTCCC AGTAAGCGCG AGTCA
>Alismobates_reticulatus
ATCAGTTACG GTTAGATGTT GACATCTACA TGGATAACTG TGGTAATTCT AGAGCTAATA
CATGCACCAA AGCCTCGACC TGGAAGAGGC GCATTTATTA GACCAAGACC AACGGGGGTG
GTGACTCTGG ATAACTGCTA ATCGCATGGC CGAGCCGGCG ATGAATTCAA GTGTCTGCCT
TATCAACTGT CGATGGTAGG TTATGCGCCT ACCATGGTTG TAACGGGTAA CGGGGAATCA
TTCGATTCCA GCCTGAGAAA CTACCACATC CAAGGAAGGC AGCAGGCACG CAAATTACCC
AAGGTAGTGA CGAAAAATAA CAATACGAGA CTCTTATGAG GCCTCGTAAT TGGAATGAGA
ACAATTTAAA TCCTTAACGA GGATCTATTG GAGGGCAAGT CTGGTGCCAG CAGCCGCGGT
AATCCAGCTC CAATAGCGTA TATTAAAGTT GTTGTTGTTA AAAAGCTCGT AGTTGGATCT
CAGTTCTAGT TGGCGGTCCA CTTGCCAGTG GCTACTGTCT TGAACATTAT CGCCTATGGT
GCTCTTCACC GAGTGTCATA GGCGATCGAT AGGTTTACTT TGAAAAAATT AGAGTGCTCA
AAGCAGGCGC CCGAATAATG TTGCATGGAA TAATGGAATA GGACCTCGGT TCTATTTTGT
TGGTCTTCGG AGCTGAGGTA ATGATTAGAG GGACAGACGG GGGCATTCGT ATTGCAGCGC
TAGAGGTGAA ATTCTTGGAC CGTTGCAAGA CGAACTAAAG CGAAAGCACC AAGAATGTTT
TCATTAATCA AGAACGAAAG TTAGAGGTTC GAAGGCGATC AGATACCGCC CTAGTTCTAA
CCATAAACGA TGCCAACCAG TAATAGGTCT GAGTTCAAAT GACTCGCGAC TTCCGGGAAA
CCAAAGTTCG GTTCAAGGGG AAGTATGGTT GCAAAGCTGA AACTTAAAGA AATTGACGGA
AGGGCACCAC CAGGAGTGGA GCCTGCGGCT TAATTTGACT CAACACGGGA AAACTCACCC
GGCCCGGACA CTGGAAGGAT TGACAGATTG AGAGCTCTTT CTTGATTCAG TGGGTGGTGG
TGCATGGCCG TTCTTAGTTG GTGGAGTGAT TTGTCTGGTT AATTCCGATA ACGAACGAGA
CTCTAGCCTA CTAAATAACG TCAGCTTCTT AGAGGGACAG GCGGTGATTC AACCGCACGA
AACAGAGCAA TAACAGGTCT GTGATGCCCT TAGATGTCCG GGGCCGCACG CGCGCTACAC
TGAAGTGATC AGCGTGCGGC CTACTCTGTC AAGAGTGGGA AACCCAATGA ACCTTCGTGA
TTGGGATATT GTAATTATTG CCCTTGAACG AGGAATTCCC AGTAAGCGCG AGTCA
>Fortuynia_rotunda
ATCAGTTACG GTTAGATATT GACAACTACA TGGATAACTG TGGTAATTCT AGAGCTAATA
CATGCACAAA AGCTTCGACC TGGAAGAGGC GCATTTATTA GACCAAGACC AATGGGGGTG
GTGACTCTGG ATAACTGCTA ATCGCATGGC CGAGCCGGCG ATGAATTCAA GTGTCTGCCT
TATCAACTGT CGATGGTAGG TTATGCGCCT ACCATGGTTG TAACGGGTAA CGGGGAATCA
TTCGATTCCA GCCTTAGACA CTACCACATC CAAGGAAGGC AGCAGGCGCG CAAATTACCC
AAGGTAGTGA CAAGAAATAA CAATACGAGA CTCTTATGAG GCCTCGTAAT TGGAATGAGA
ACAATTTAAA TCCTTAACGA GGATCTATTG GAGGGCAAGT CTGGTGCCAG CAGCCGCGGT
AATCCAGCTC CAATAGCGTA TATTAAAGTT GTTGTTGTTA AAAAGCTCGT AGTTGGATCT
CAGTTCTAGT TGATGGTCCA CTTGCCAGTG GTTACTGTCT TGAGCAGTAT CGCCTATGGT
GCTCTTCACC GAGTGTCATA GGCGATCGAT AGGTTTACTT TGAAAAAATT AGAGTGCTCA
AAGCAGGCGC CTGAATACTG TTGCATGGAA TAATGGAATA GGACCTCGGT TCTATTTTGT
TGGTCTTCGG AACTGAGGTA ATGATTAGAG GGACAGACGG GGGCATTCGT ATTGCAGCGC
TAGAGGTGAA ATTCTTGGAC CGTTGCAAGA CGAACTAAAG CGAAAGCACC AAGAATGTTT
TCATTAATCA AGAACGAAAG TTAGAGGTTC GAAGGCGATC AGATACCGCC CTAGTTCTAA
CCATAAACGA TGCCAACCAG TAATAGGTCT GAGTTCAAAT GACTCGCGAC TTCCGGGAAA
CCAAAGTTCG GTTCAAGGGG GAGTATGGTT GCAAAGCTGA AACTTAAAAA AATTGACGGA
AGGGCACCAC CAGGAGTGGA GCCTGCGGCT TAATTTGACT CAACACGGGA AAACTCACCC
GGCCCGGACA CTGGAAGGAT TGACAGATTG AGAGCTCTTT CTTGATTCAG TGGGTGGTGG
TGCATGGCCG TTCTTAGTTG GTGGAGTGAT TTGTCTGGTT AATTCCGATA ACGAACGAGA
CTCTAGCCTA CTAAATAACG TCAACTTCTT AGAGGGACAG GCGGTGATTC AACCGCACGA
AACAGAGCAA TAACAGGTCT GTGATGCCCT TAGATGTCCG GGGCCGCACG CGCGCTACAC
TGAAGTGATC AGCGTGCGGC CTACTCTGTC AAGAGTGGGA AACCCAATGA ACCTTCGTGA
TTGGGATATT GTAATTATTG CCCTTGAACG AGGAATTCCC AGTAAGCGCG AGTCA
>Hydrozetes_confervae
ATCAGTTACG GTTAGATGTT GACGTCTACA TGGATAACTG TGGTAATTCT AGAGCTAATA
CATGCACAAA AGCTTCGACC TGGAAGAAGC GCATTTATTA GACCAAGACC AATGGGGGTG
GTGACTCTGG ATAACTGCTA ATCGCATGGC CGAGCCGGCG ATGAATTCAA GTGTCTGCCT

TATCAACTGT CGATGGTAGG TTATGCGCCT ACCATGGTTG TAACGGGTAA CGGGGAATCA
TTCGATTCCA GCCTGAGAAA CTACCACATC CAAGGAAGGC AGCAGGCACG CAAATTACCC
AAGGTAGTGA CGAAAAATAA CAATACGGGA CTCTTATGAG GCCCCGTAAT TGGAATGAGA
ACAATCTAAA TCCTTAACGA GGATCTATTG GAGGGCAAGT CTGGTGCCAG CAGCCGCGGT
AATCCAGCTC CAATAGCGTA TATTAAAGTT GTTGTGGTTA AAAAGCTCGT AGTTGGATCT
CAGTTCGAGT CGACGGTCCA CTTGCCAGTG GTTACTGTCT TGAACATTAT CGCCTATGGT
GCTCTTCACC GAGTGTCATA GGCGATCGAT ACGTTTACTT TGAAAAAATT AGAGTGCTCA
AAGCAGGCGC CCGAATAATG TTGCATGGAA TAATGGAATA GGACCTCGGT TCTATTTTGT
TGGTCTTCGG AACTGAGGTA ATGATTAGAG GGACAGACGG GGGCATTCGT ATTGCGACGC
TAGAGGTGAA ATTCTTGGAC CGTTGCAAGA CGAACTAAAG CGAAGGCACC AAGAATGTTT
TCATTAATCA AGAACGAAAG TTAGCGGATC GAAGGCGATC AGATACCGCC CTAGTGCTAA
CCATAAACGA TGCCAACCAG TAATAAGCCT GAGTTCAAAT GACTCGGGAC TTCCGGGAAA
CCAAAGTTCG GTTCCAGGGG AAGTATGGTT GCAAAGCTGA AACTTAAAGA AATTGACGGA
AGGGCACCAC CAGGAGTGGA GCCTGCGGCT TAATTTGACT CAACACGGGA AAACTCACCC
GGCCCGGACA CTGGAAGGAT TGACAGATTG AGAGCTCTTT CTTGATTCAG TGGGTGGTGG
TGCATGGCCG TTCTTAGTTG GTGGAGTGAT TTGTCTGGTT AATTCCGATA ACGAACGAGA
CTCTAGCCTA CTAAATAACG CCGACTTCTT AGAGGGACAG GCGGTGATTC AACCGCACGA
AACAGAGCAA TAACAGGTCT GTGATGCCCT TAGATGTCCG GGGCCGCACG CGCGCTACAC
TGAAGTGATC AGCGTGCAGC CTACTCTGTC AAGAGTGGGA AACCCAATGA ACCTTCGTGA
TTGGGATATT GTAATTATTC CCCTTGAACG AGGAATTCCC AGTAAGCGCG AGTCA
>Hydrozetes_lacustris
ATCAGTTACG GTTAGATGTT GACGTCTACA TGGATAACTG TGGTAATTCT AGAGCTAATA
CATGCACAAA AGCTTCGACC TGGAAGAAGC GCATTTATTA GACCAAGACC AATGGGGGTG
GTGACTCTGG ATAACTGCTA ATCGCATGGC CGAGCCGGCG ATGAATTCAA GTGTCTGCCT
TATCAACTGT CGATGGTAGG TTATGCGCCT ACCATGGTTG TAACGGGTAA CGGGGAATCA
TTCGATTCCA GCCTGAGAAA CTACCACATC CAAGGAAGGC AGCAGGCACG CAAATTACCC
AAGGTAGTGA CGAAAAATAA CAATACGGGA CTCTTATGAG GCCCCGTAAT TGGAATGAGA
ACAATCTAAA TCCTTAACGA GGATCTATTG GAGGGCAAGT CTGGTGCCAG CAGCCGCGGT
AATCCAGCTC CAATAGCGTA TATTAAAGTT GTTGTGGTTA AAAAGCTCGT AGTTGGATCT
CAGTTCGAGT CGACGGTCCA CTTGCCAGTG GTTACTGTCT TGAACATTAT CGCCTATGGT
GCTCTTCACC GAGTGTCATA GGCGATCGAT ACGTTTACTT TGAAAAAATT AGAGTGCTCA
AAGCAGGCGC CCGAATAATG TTGCATGGAA TAATGGAATA GGACCTCGGT TCTATTTTGT
TGGTCTTCGG AACTGAGGTA ATGATTAGAG GGACAGACGG GGGCATTCGT ATTGCGACGC
TAGAGGTGAA ATTCTTGGAC CGTTGCAAGA CGAACTAAAG CGAAGGCACC AAGAATGTTT
TCATTAATCA AGAACGAAAG TTAGCGGATC GAAGGCGATC AGATACCGCC CTAGTGCTAA
CCATAAACGA TGCCAACCAG TAATAAGCCT GAGTTCAAAT GACTCGGGAC TTCCGGGAAA
CCAAAGTTCG GTTCCAGGGG AAGTATGGTT GCAAAGCTGA AACTTAAAGA AATTGACGGA
AGGGCACCAC CAGGAGTGGA GCCTGCGGCT TAATTTGACT CAACACGGGA AAACTCACCC
GGCCCGGACA CTGGAAGGAT TGACAGATTG AGAGCTCTTT CTTGATTCAG TGGGTGGTGG
TGCATGGCCG TTCTTAGTTG GTGGAGTGAT TTGTCTGGTT AATTCCGATA ACGAACGAGA
CTCTAGCCTA CTAAATAACG CCGACTTCTT AGAGGGACAG GCGGTGATTC AACCGCACGA
AACAGAGCAA TAACAGGTCT GTGATGCCCT TAGATGTCCG GGGCCGCACG CGCGCTACAC
TGAAGTGATC AGCGTGCAGC CTACTCTGTC AAGAGTGGGA AACCCAATGA ACCTTCGTGA
TTGGGATATT GTAATTATTC CCCTTGAACG AGGAATTCCC AGTAAGCGCG AGTCA
>Scheloribates_pallidulus
ATCAGTTACG GTTAGATGTT GACATCTACA TGGATAACTG TGGAAAATCT AGAGCTAATA
CATGCACAAA AGCTTTGACC TGGAAAAAGC GCATTTATTA GACCAAGACC AATGGGTGTG
GTGACTCTGG ATAACTGCTA ATCGCATGGC CGAGCCGGCG ATGAATTCAA GTGTCTGCCT
TATCAACTGT CGATGGTAGG TTATGCGCCT ACCATGGTTG TAACGGGTAA CGGGGAATCA
TTCGATTCCG GCACGAGAAT CCACCACATC CAAGGAAGGC AGCAGGCGCG CAAATTACCC
AAGGTAGTGA CGAAAAATAA CAATACGGGA CTCTTATGAG GCCCCGTAAT TGGAATGAGA
ACAATCTAAA TCCTTAACGA GGATCTATTA GAGGGCAAGT CTGGTGCCAG CAGCCGCGGT
AATCCAGCTC TAATAGCGTA TACTAAAGTT GTTGTGGTTA AAAAGCTCGT AGTTGGATCT
CAGTTCTAGT CGACGGTCCA CTTGCCAGTG GTTACTGTCT TGAACATTAT CGCTTACGGT
ATTCTTAATC GAGTGTCGTA AGCGATCGAT ACGTTTACTT TGAAAAAATT AGAGTGCTCA
AAGCAGGCGC CCGAATAATC TTGCATGGAA TAATGGAATA GGACCTCGGT TCTATTTTGT
TGGTCTTCGG AACCGAGGTA ATGATTAGAG GGACAGACGG GGGCATTCGT ATTGCGACGC
TAGAGGTGAA ATTCTTGGAC CGTTGCAAGA CGAACTAAAG CGAAGGCACC AAGAATGTTT
TCATTAATCA AGAACGAAAG TTAGCGGATC GAAGGCGATC AGATACCGCC CTAGTGCTAA
CCATAAACGA TGCCAACCAG TAATATGCCT GAGTTCAAAT GACTCGGGAC TTCCGGGAAA

CCAAAGTTCG GTTCCAGGGG AAGTATGGTT GCAAAGCTGA AACTTAAAGA AATTGACGGA
AGGGCACCAC CAGGAGTGGA GCCTGCGGCT TAATTTGACT CAACACGGGA AAACTCACCC
GGCCCGGACA CTGGAAGGAT TGACAGATTG AGAGCTCTTT CTTGATTCAG TGGGTGGTGG
TGCATGGCCG TTCTTAGTTG GTGGAGTGAT TTGTCTGGTT AATTCCGATA ACGAACGAGA
CTCTAGCCTA CTAAATAACG CTAACTTCTT AGAGGGACAG GCGGTGATTC AACCGCACGA
AACAGAGCAA TAACAGGTCT GTGATGCCCT TAGATGTCCG GGGCCGCACG CGCGCTACAC
TGAAGTGATC AACGCGCAAC CTACTCTGTC AAGAGTGGGA AACCCATTGA ACCTTCGTGA
TTGGGATTTT GTAATTATAC CACATGAACG AGGAATTCCC AGTAAGCGCG AGTCA
>Hemileius_singularis
ATCAGTTACG GTTAGATGTT GACATCTACA TGGATAACTG TGGAAAATCT AGAGCTAATA
CATGCACAAA AGCTTTGACC TGGAAGAAGC GCATTTATTA GACCAAGACC AATGGGTGTG
GTGACTCTGG ATAACTGCTA ATCGTATGGC CGTGCCGGCG ATGAATTCAA GTGTCTGCCT
TATCAACTGT CGATGGTAGG TTATGCGCCT ACCATGGTTG TAACGGGTAA CGGGGAATCA
TTCGATTCCG GCATGAGAAT CCACCACATC CAAGGAAGGC AGCAGGCGCG CAAATTACCC
AAGGTAGTGA CGAAAAATAA CAATACGGGA CTCTTATGAG GCCCCGTAAT TGGAATGAGA
ACAATCTAAA TCCTTAACGA GGATCTATTA GAGGGCAAGT CTGGTGCCAG CAGCCGCGGT
AATCCAGCTC TAATAGCGTA TATTAAAGTT GTTGTGGTTA AAAAGCTCGT AGTTGGATTT
CAGTTCTAGT CGATGGTCCA CTTGCCAGTG GTTACTGTCT TGAACATTAT CGCCTACGGT
ATTCTTAATC GAGTGTCGTA GGCGATCGGT ACGTTTACTT TGAAAAAATT AGAGTGCTCA
AAGCAGGCGC CCGAATAATG TTGCATGGAA TAATGGAATA GGACCTCGGT TCTATTTTGT
TGGTCTTCGG AACCGAGGTA ATGATTAGAG GGACAGACGG GGGCATTCGT ATTGCGACGC
TAGAGGTGAA ATTCTTGGAC CGTTGCAAGA CGAACTAAAG CGAAGGCACC AAGAATGTTT
TCATTAATCA AGAACGAAAG TTAGCGGATC GAAGGCGATC AGATACCGCC CTAGTGCTAA
CCATAAACGA TGCCAACCAG TAATATGCCT GAGTTCAAAT GACTCGGGAC TTCCGGGAAA
CCAAAGTTCG GTTCCAGGGG AAGTATGGTT GCAAAGCTGA AACTTAAAGA AATTGACGGA
AGGGCACCAC CAGGAGTGGA GCCTGCGGCT TAATTTGACT CAACACGGGA AAACTCACCC
GGCCCGGACA CTGGAAGGAT TGACAGATTG AGAGCTCTTT CTTGATTCAG TGGGTGGTGG
CGCATGGCCG TTCTTAGTTG GTGGAGTGAT TTGTCTGGTT AATTCCGATA ACGAACGAGA
CTCTAGCCTA CTAAATAACG CTAACTTCTT AGAGGGACAG GCGGTGATTC AACCGCACGA
AACAGAGCAA TAACAGGTCT GTGATGCCCT TAGATGTCCG GGGCCGCACG CGCGCTACAC
TGAAGTGACC AACGCGCAAC CTGCTCTGTC AAGAGTGGGA AACCCATTGA ACCTTCGTGA
TTGGGATTTT GTAATTATAC CACATGAACG AGGAATTCCC AGTAAGCGCG AGTCA
>Oripoda_sp
ATCAGTTACG GTTAGATGTT GACATCTACA TGGATAACTG TGGAAAATCT AGAGCTAATA
CATGCACAAA AGCTTCGACC TGGAAGAAGC GCATTTATTA GACCAAGACC AATGGGTGTG
GTGACTCTGG ATAACTGCTA ATCGCATGGC CGAGCCGGCG ATGAATTCAA GTGTCTGCCT
TATCAACTGT CGATGGTAGG TTATGCGCCT ACCATGGTTG TAACGGGTAA CGGGGAATCA
TTCGATTCCG GCTTGAGAAT CCACCACATC CAAGGAAGGC AGCAGGCGCG CAAATTACCC
AAGGTAGTGA CGAAAAATAA CAATACGGGA CTCTTATGAG GCCCCGTAAT TGGAATGAGA
ACAATCTAAA TCCTTAACGA GGATCTATTA GAGGGCAAGT CTGGTGCCAG CAGCCGCGGT
AATCCAGCTC TAATAGCGTA TACTAAAGTT GTTGTGGTTA AAAAGCTCGT AGTTGGATCT
CAGTTCGAGT CGACGGTCCA CTTGCCAGTG GCTACTGTCT TGAACATTAT CGCCTACGGT
ATTCTTAATC GAGTGTCGTA GGCGATCGAT ACGTTTACTT TGAAAAAATT AGAGTGCTCA
AAGCAGGCGC CCGAATAATG TTGCATGGAA TAATGGAATA GGACCTCGGT TCTATTTTGT
TGGTCTTCGG AACCGAGGTA ATGATTAGAG GGACAGACGG GGGCATTCGT ATTGCGACGC
TAGAGGTGAA ATTCTTGGAC CGTTGCAAGA CGAACTAAAG CGAAGGCACC AAGAATGTTT
TCATTAATCA AGAACGAAAG TTAGCGGATC GAAGGCGATC AGATACCGCC CTAGTGCTAA
CCATAAACGA TGCCAACCAG TAATATGCCT GAGTTCAAAT GACTCGGGAC TTCCGGGAAA
CCAAAGTTCG GTTCCAGGGG AAGTATGGTT GCAAAGCTGA AACTTAAAGA AATTGACGGA
AGGGCACCAC CAGGAGTGGA GCCTGCGGCT TAATTTGACT CAACACGGGA AAACTCACCC
GGCCCGGACA CTGGAAGGAT TGACAGATTG AGAGCTCTTT CTTGATTCAG TGGGTGGTGG
TGCATGGCCG TTCTTAGTTG GTGGAGTGAT TTGTCTGGTT AATTCCGATA ACGAACGAGA
CTCTAGCCTA CTAAATAACG CCAACTTCTT AGAGGGACAG GCGGTGATTC AACCGCACGA
AACAGAGCAA TAACAGGTCT GTGATGCCCT TAGATGTCCG GGGCCGCACG CGCGCTACAC
TGAAGTGATC AACGCGCAAC CTACTCTGTC AAGAGTGGGA AACCCATTGA ACCTTCGTGA
TTGGGATTTT GTAATTATAC CACATGAACG AGGAATTCCC AGTAAGCGCG AGTCA
>Protoribates_hakonensis
ATCAGTTACG GTTAGATGTT GACATTTACA TGGATAACTG TGGAAAATCT AGAGCTAATA
CATGCACAAA AGCTTTGACC TGGGAAGAGC GCATTTATCA GACCAAGACC AATGGGTGTG
GCGACTCTGG ATAACTGCTA ATCGCATGGC CGAGCCGGCG ATGAATTCAA GTGTCTGCCT

TATCAACTGT CGATGGTAGG TTATGCGCCT ACCATGGTTG TAACGGGTAA CGGGGAATCA
TTCGATTCCG GCTTGAGAAT CCACCACATC CAAGGAAGGC AGCAGGCGCG CAAATTACCC
AAGGTAGTGA CGAAAAATAA CAATACGGGA CTCTTATGAG GCCCCGTAAT TGGAATGAGA
ACAATCTAAA TCCTTAACGA GGATCTATTA GAGGGCAAGT CTGGTGCCAG CAGCCGCGGT
AATCCAGCTC TAATAGCGTA TACTAAAGTT GTTGTGGTTA AAAAGCTCGT AGTTGGATCT
CAGTTCGAGT CGTCGGTCCA CTTGCCAGTG GCTACTGACT TGAACATTAC CGCCTACAGT
GCTCTTAATC GAGTGTTGTA GGCGATCGGT ACGTTTACTT TGAAAAAATT AGAGTGCTCA
AAGCAGGCGC CCGAATAATG TTGCATGGAA TAATGGAATA GGACCTCGGT TCTATTTTGT
TGGTCTTCGG AACCGAGGTA ATGATTAGAG GGACAGACGG GGGCATTCGT ATTGCGACGC
TAGAGGTGAA ATTCTTGGAC CGTTGCAAGA CGAACTAGAG CGAAGGCACC AAGAATGTTT
TCATTAATCA AGAACGAAAG TTAGCGGATC GAAGGCGATC AGATACCGCC CTAGTGCTAA
CCATAAACGA TGCCAACCAG TAATATGCCT GAGTTCAAAT GACTCGGGAC TTCCGGGAAA
CCAAAGTTCG GTTCCAGGGG AAGTATGGTT GCAAAGCTGA AACTTAAAGA AATTGACGGA
AGGGCACCAC CAGGAGTGGA GCCTGCGGCT TAATTTGACT CAACACGGGA AAACTCACCC
GGCCCGGACA CTGGAAGGAT TGACAGATTG AGAGCTCTTT CTTGATTCAG TGGGTGGTGG
TGCATGGCCG TTCTTAGTTG GTGGAGTGAT TTGTCTGGTT AATTCCGATA ACGAACGAGA
CTCTAGCCTA CTAAATAACG CCCACTTCTT AGAGGGACAG GCGGTGATTC AACCGCACGA
AACAGAGCAA TAACAGGTCT GTGATGCCCT TAGATGTCCG GGGCCGCACG CGCGCTACAC
TGAAGTGATC AACGCGCAAC CTACTCTGTC AAGAGTGGGA AACCCATTGA ACCTTCGTGA
TTGGGATTTT GTAATTATAC CACATGAACG AGGAATTCCC AGTAAGCGCG AGTCA
>Rostrozetes_ovulum
ATCAGTTACG GTTAGATGTT GACATCTACA TGGATAACTG TGGAAAATCT AGAGCTAATA
CATGCACAAA AGCTTTGACC TGGGAAAAGC GCATTTATTA GACCAAGACC AATGGGTGTG
GTGACTCTGG ATAACTGCTG ATCGCATGGC CGAGCCGGCG ACGAATTCAA GTGTCTGCCT
TATCAACTGT CGATGGTAGG TTATGCGCCT ACCATGGTTG TAACGGGTAA CGGGGAATCA
TTCGATTCCG GCTTGAGAAT CCACCACATC CAAGGAAGGC AGCAGGCGCG CAAATTACCC
AAGGTAGTGA CGAAAAATAA CAATACGGGA CTCTTATGAG GCCCCGTAAT TGGAATGAGA
ACAATCTAAA TCCTTAACGA GGATCTATTA GAGGGCAAGT CTGGTGCCAG CAGCCGCGGT
AATCCAGCTC TAATAGCGTA TACTAAAGTT GTTGTGGTTA AAAAGCTCGT AGTTGGATCT
CAGTTCGAGT CGTCGGTCCA CTTGCCAGTG GTTACTGACT TGAACATTAC CGCCTACGGT
ATTCTTAATC GAGTGTCGTA GGCGATCGGT ACGTTTACTT TGAAAAAATT AGAGTGCTCA
AAGCAGGCGC CCGAATAATG TTGCATGGAA TAATGGAATA GGACCTCGGT TCTATTTTGT
TGGTCTTCGG AACCGAGGTA ATGATTAGAG GGACAGACGG GGGCATTCGT ATTGCGACGC
TAGAGGTGAA ATTCTTGGAC CGTTGCAAGA CGAACTAAAG CGAAGGCACC AAGAATGTTT
TCATTAATCA AGAACGAAAG TTAGCGGATC GAAGGCGATC AGATACCGCC CTAGTGCTAA
CCATAAACGA TGCCAACCAG TAATATGCCT GAGTTCAAAT GACTCGGGAC TTCCGGGAAA
CCAAAGTTCG GTTCCAGGGG AAGTATGGTT GCAAAGCTGA AACTTAAAGA AATTGACGGA
AGGGCACCAC CAGGAGTGGA GCCTGCGGCT TAATTTGACT CAACACGGGA AAACTCACCC
GGCCCGGACA CTGGAAGGAT TGACAGATTG AGAGCTCTTT CTTGATTCAG TGGGTGGTGG
TGCATGGCCG TTCTTAGTTG GTGGAGTGAT TTGTCTGGTT AATTCCGATA ACGAACGAGA
CTCTAGCCTA CTAAATAACG CCAACTTCTT AGAGGGACAG GCGGTGATTC AACCGCACGA
AACAGAGCAA TAACAGGTCT GTGATGCCCT TAGATGTCCG GGGCCGCACG CGCGCTACAC
TGAAGTGATC AACGCGCCAC CTACTCTGTC AAGAGTGGGA AACCCATTGA ACCTTCGTGA
TTGGGATTTT GTAATTATAC CACATGAACG AGGAATTCCC AGTAAGCGCG AGTCA
>Maculobates_bruneiensis
ATCAGTTACG GTTTGATGTT GACATCTACA TGGATAACTG TGGAAAATCT AGAGCTAATA
CATGCCATGA AGCCCAGACC TGGAACGGGC GCATTTATTA GACCAAGACC AATGGGTGTG
GTGACTCTGG ATAACTGCTG ATCGCATGGC CGAGCCGGCG ACGAATTCAA GTGTCTGCCT
TATCAACTGT CGATGGTAGG CTATGCGCCT ACCATGGTTG TAACGGGTAA CGGGGAATCA
TTCGGTTCCG GCTTGAGAAA CCACTACATC CAAGGAAGGC AGCAGGCGCG CAAATTACCC
AAGGTAGTGA CGAAAAATAA CAATACGGGA CTCTTATGAG GCCCCGTAAT TGGAATGAGA
ACAATCTAAA TCCTTAACGA GGATCTATTA GAGGGCAAGT CTGGTGCCAG CAGCCGCGGT
AATCCAGCTC TAATAGCGTA TACTAAAGTT GTTGTGGTTA AAAAGCTCGT AGTTGGATCT
CAGTTCGAGT CGTTGGTCCA CTTGCCAGTG GTTACTGATT TGAACATTAT CGCCTGTAGT
GCTCTTAATC GAGTGTTGCA GGCGATCGAT ACGTTTACTT TGAGAAAATT AGAGTGCTCA
AAGCAGGCGC CCGAATAATG TTGCATGGAA TAATGGAATA GGACCTCGGT TCTATTTTGT
TGGTCTTCGG AACCGAGGAA ATGATTAGAG GGACAGACGG GGGCATTCGT ATTGCGACGC
TAGAGGTGAA ATTCTTGGAC CGTTGCAAGA CGAACTAAAG CGAAGGCACC AAGAATGTTT
TCATTAATCA AGAACGAAAG TTAGCGGATC GAAGGCGATC AGATACCGCC CTAGTGCTAA
CCATAAACGA TGCCAACCAG TAATATGCCT GAGTTCAAAT GACTCGGGAC TTCCGGGAAA

CCAAAGTTCG GTTCCAGGGG AAGTATGGTT GCAAAGCTGA AACTTAAAGA AATTGACGGA
AGGGCACCAC CAGGAGTGGA GCCTGCGGCT TAATTTGACT CAACACGGGA AAACTCACCC
GGCCCGGACA CTAGAAGGAT TGACAGATTG AGAGCTCTTT CTTGATTCAG TGGGTGGTGG
TGCATGGCCG TTCTTAGTTG GTGGAGTGAT TTGTCTGGTT AATTCCGATA ACGAACGAGA
CTCTAGCCTA CTAAATAACG CCAACTTCTT AGAGGGACAG GCGGTGATTC AACCGCACGA
AACAGAGCAA TAACAGGTCT GTGATGCCCT TAGATGTCCG GGGCCGCACG CGCGCTACAC
TGAAGTGATC AACGCGCAAC CTACTCTGTC AAGAGTGGGA AACCCAATGA ACCTTCGTGA
TTGGGATTTT GTAATTATAC CACATGAACG AGGAATTCCC AGTAAGCGCG AGTCA
>Peloribates_acutus
ATCAGTTACG GTTAGATGTT GACATTTACA TGGATAACTG TGGAAAATCT AGAGCTAATA
CATGCACAAA AGCCTCGACC TGGGAAAGGC GCATTTATTA GACCAAGACC AATGGGTGTG
GTGACTCTGG ATAACTGCTG ATCGCATGGC CGTGCCGGCG ACGAATTCAA GTGTCTGCCT
TATCAACTGT CGATGGTAGG TTATGCGCCT ACCATGGTTG TAACGGGTAA CGGGGAATCA
TTCGATTCCG GCTTGAGAAT CCACCACATC CAAGGAAGGC AGCAGGCGCG CAAATTACCC
AAGGTAGTGA CGAAAAATAA CAATACGGGA CTCTTATGAG GCCCCGTAAT TGGAATGAGA
ACAATCTAAA TCCTTAACGA GGATCTATTA GAGGGCAAGT CTGGTGCCAG CAGCCGCGGT
AATCCAGCTC TAATAGCGTA TACTAAAGTT GTTGTGGTTA AAAAGCTCGT AGTTGGATCT
CAGTTCTAGT CTGCGGTCCA CTTGCCAGTG GTTACTGTAC TGAACATTAT CGTATTCGGT
GCTCTTAATC GAGTGTCGTA TACGATCGAT ACGTTTACTT TGAAAAAATT AGAGTGCTCA
AAGCAGGCGC CCGAATAATG TTGCATGGAA TAATGGAATA GGACCTCGGT TCTATTTTGT
TGGTCTTCGG AACCGAGGTA ATGATTAGAG GGACAGACGG GGGCATTCGT ATTGCGACGC
TAGAGGTGAA ATTCTTGGAC CGTTGCAAGA CGAACTAAAG CGAAGGCACC AAGAATGTTT
TCATTAATCA AGAACGAAAG TTAGCGGATC GAAGGCGATC AGATACCGCC CTAGTGCTAA
CCATAAACGA TGCCAACCAG TAATATGCCT GAGTTCAAAT GACTCGCGAC TTCCGGGAAA
CCAAAGTTCG GTTCCAGGGG AAGTATGGTT GCAAAGCTGA AACTTAAAGA AATTGACGGA
AGGGCACCAC CAGGAGTGGA GCCTGCGGCT TAATTTGACT CAACACGGGA AAACTCACCC
GGCCCGGACA CTGGAAGGAT TGACAGATTG AGAGCTCTTT CTTGATTCAG TGGGTGGTGG
TGCATGGCCG TTCTTAGTTG GTGGAGTGAT TTGTCTGGTT AATTCCGATA ACGAACGAGA
CTCTAGCCTA CTAAATAACG CCAGCTTCTT AGAGGGACAG GCGGTGATTC AACCGCACGA
AACAGAGCAA TAACAGGTCT GTGATGCCCT TAGATGTCCG GGGCCGCACG CGCGCTACAC
TGAAGTGATC AACGCGCGTC CTACTCTGTC AAGAGTGGGA AACCCATTGA ACCTTCGTGA
TTGGGATTTT GTAATTATAC CACATGAACG AGGAATTCCC AGTAAGCGCG AGTCA
>Achipteria_coleoptrata
ATCAGTTACG GTTAGATGTT GACATCTACA TGGATAACTG TGGTAATTCT AGAGCTAATA
CATGCACAAA AGCTTCGACC TGGAAGAAGC GCATTTATTA GACCAAGACC AATGGGGGTG
GTGACTCTGA ATAACTGCTA ATCGCATGGC CGAGCCGGCG ATGAATTCAA GTGTCTGCCT
TATCAACTGT CGATGGTAGG TTATGCGCCT ACCATGGTTG TAACGGGTAA CGGGGAATCA
TTCGATTCCA GCCTGAGAAA CTACCACATC CAAGGAAGGC AGCAGGCACG CAAATTACCC
AAGGTAGTGA CGAAAAATAA CAATACGAGA CTCTTATGAG GCCTCGTAAT TGGAATGAGA
ACAATCTAAA TCCTTAACGA GGATCTATTG GAGGGCAAGT CTGGTGCCAG CAGCCGCGGT
AATCCAGCTC CAATAGCGTA TATTAAAGTT GTTGTGGTTA AAAAGCTCGT AGTTGGATCT
CAGTTCGAGT CGACGGTCCA CTTGCCAGTG GTTACTGTCT TGAACATTAT CGCCTATGGT
GTTCTTAACC GAATGTCATA GGCGATCGAT ACGTTTACTT TGAAAAAATT AGAGTGCTCA
AAGCAGGCGC CCGAATAATG TTGCATGGAA TAATGGAATA GGACCTCGGT TCTATTTTGT
TGGTCTTCGG AACTGAGGTA ATGATTAGAG GGACAGACGG GGGCATTCGT ATTGCGACGC
TAGAGGTGAA ATTCTTGGAC CGTTGCAAGA CGAACTAAAG CGAAGGCACC AAGAATGTTT
TCATTAATCA AGAACGAAAG TTAGCGGATC GAAGGCGATC AGATACCGCC CTAGTGCTAA
CCATAAACGA TGCCAACCAG TAATAAGCCT GAGTTCAAAT GACTCGGGAC TTCTGGGAAA
CCAAAGTTCG GTTCCAGGGG AAGTATGGTT GCAAAGCTGA AACTTAAAGA AATTGACGGA
GCGGCACCAC CAGGAGTGGA GCCTGCGGCT TAATTTGACT CAACACGGGA AAACTCACCC
GGCCCGGACA CTGGAAGGAT TGACAGATTG AGAGCTCTTT CTTGATTCAG TGGGTGGTGG
TGCATGGCCG TTCTTAGTTG GTGGAGTGAT TTGTCTGGTT AATTCCGATA ACGAACGAGA
CTCTAGCCTA CTAAATAACG CCAACTTCTT AGAGGGACAG GCGGTGACTC AACCGCACGA
AACAGAGCAA TAACAGGTCT GTGATGCCCT TAGATGTCCG GGGCCGCACG CGCGCTACAC
TGAAGTGATC AGCGTGCAGC CTACTCTGTC AAGAGTGGGA AACCCAATGA ACCTTCGTGA
TTGGGATATT GTAATTATTC CCCTTGAACG AGGAATTCCC AGTAAGCGCG AGTCA
>Euzetes_globulus
ATCAGTTACG GTTAGATGTT GACATTTACA TGGATAACTG TGGTAATTCT AGAGCTAATA
CATGCACAAA AGCTTCGACC TGGAAGAAGC GCATTTATTA GACCAAGACC AATGGGGGTG
GTGACTCTGT ATAACTGCTA ATCGCATGGC CGTGCCGGCG ATGAATTCAA GTGTCTGCCT

```
TATCAACTGT CGATGGTAGG CTATGCGCCT ACCATGGTTG TAACGGGTAA CGGGGAATCA
TTCGATTCCA GCCTGAGAAA CTACCACATC CAAGGAAGGC AGCAGGCACG CAAATTACCC
AAGGTAGTGA CGAAAAATAA CAATACGGGA CTCTTATGAG GCCCCGTAAT TGGAATGAGA
ACAATCTAAA TCCTTAACGA GGATCTATTG GAGGGCAAGT CTGGTGCCAG CAGCCGCGGT
AATCCAGCTC CAATAGCGTA TATTAAAGTT GTTGTGGTTA AAAAGCTCGT AGTTGGATCT
CAGTTCGAGT CGGTGGTCCA CTTGCCAGTG GTTACTACTT TGAACATTAT CGCCTATGGT
GCTCTTCACC GAGTGTCATA GGCGATCGAT ACGTTTACTT TGAAAAAATT AGAGTGCTCA
AAGCAGGCGC CCGAATAATG TTGCATGGAA TAATGGAATA GGACCTCGGT TCTATTTTGT
TGGTCTTCGG AACTGAGGTA ATGATTAGAG GGACAGACGG GGGCATTCGT ATTGCGGCGC
TAGAGGTGAA ATTCTTGGAC CGTCGCAAGA CGAACTAAAG CGAAAGCACC AAGAATGTTT
TCATTAATCA AGAACGAAAG TTAGAGGTTC GAAGGCGATC AGATACCGCC CTAGTTCTAA
CCATAAACGA TGCCGACCAG TAATTAAGCT GAGTTCAAAT GACTCGCGAC TTCCGGGAAA
CCAAAGTTCG GTTCCAGGGG AAGTATGGTT GCAAAGCTGA AACTTAAAGA AATTGACGGA
AGGGCACCAC CAGGAGTGGA GCCTGCGGCT TAATTTGACT CAACACGGGA AAACTCACCC
GGCCCGGACA CTGGAAGGAT TGACAGATTG AGAGCTCTTT CTTGATTCAG TGGGTGGTGG
TGCATGGCCG TTCTTAGTTG GTGGAGTGAT TTGTCTGGTT AATTCCGATA ACGAACGAGA
CTCTAGCCTA CTAAATAACG CTAACTTCTT AGAGGGACAG GCGGTGATTC AACCGCACGA
AACAGAGCAA TAACAGGTCT GTGATGCCCT TAGATGTCCG GGGCCGCACG CGCGCTACAC
TGAAGTGATC AGCGTGCAGC CTACTCTGTC AAGAGTGGGA AACCCAATGA ACCTTCGTGA
TTGGGATATT GTAATTATTC CCCTTGAACG AGGAATTCCC AGTAAGCGCG AGTCA
>Platynothrus_peltifer
ATCAGTTACG GTTAGATGTT GACATCTACA TGGATAACTG TGGTAATTCT AGAGCTAATA
CATGCACAAA AGCTTCGACC TGGAAGAAGC GCATTTATTA GAACAAGACC AATGGGGGTG
GTGACTCTGG ATAACTGCTA ATCGCATGGC CGTGCCGGCG ATGAATTCAA GTGTCTGCCT
TATCAACTGT CGATGGTAGG TTATGCGCCT ACCATGGTTG TAACGGGTAA CGGGGAATCA
TTCGGTTCCA GCCTGAGAAA CTACCACATC CAAGGAAGGC AGCAGGCACG CAAATTACCC
AAGGTAGTGA CGAAAAATAA CAATACGGGA CTCTTATGAG GCCCCGTAAT TGGAATGAGA
ACAATCTAAA TCCTTAACGA GGATCTATTG GAGGGCAAGT CTGGTGCCAG CAGCCGCGGT
AATCCAGCTC CAATAGCGTA TATTAAAGTT GTTGTGGTTA AAAAGCTCGT AGTTGGATCT
CAGTTCGAGT CGGCGGTCCA CTTGCCAGTG GTTACTGCTT TGAACATTAT CGCCTATGGT
GCTCTTTACC GAGTGTCATA GGCGATCGGT ACGTTTACTT TGAAAAAATT AGAGTGCTCA
AAGCAGGCGC CCGAATAATG TTGCATGGAA TAATGGAATA GGACCTCGGT TCTATTTTGT
TGGTCTTCGG AACTGAGGTA ATGATTAGAG GGACAGACGG GGGCATTCGT ATTGCGGCGC
TAGAGGTGAA ATTCTTGGAC CGTCGCAAGA CGAACTAAAG CGAAAGCACC AAGAATGTTT
TCATTAATCA AGAACGAAAG TTAGAGGTTC GAAGGCGATC AGATACCGCC CTAGTTCTAA
CCATAAACGA TGCCAACCAG TAATCCGTCT GAGTTCAAAT GACTCGCGAC TTCCGGGAAA
CCAAAGTTCG GTTCCAGGGG AAGTATGGTT GCAAAGCTGA AACTTAAAGA AATTGACGGA
AGGGCACCAC CAGGAGTGGA GCCTGCGGCT TAATTTGACT CAACACGGGA AAACTCACCC
GGCCCGGACA CTGGAAGGAT TGACAGATTG AGAGCTCTTT CTTGATTCAG TGGGTGGTGG
TGCATGGCCG TTCTTAGTTG GTGGAGCGAT TTGTCTGGTT AATTCCGATA ACGAACGAGA
CTCTAGCCTA CTAAATAACG CCAACTTCTT AGAGGGACAG GCGGCGATTC AGCCGCACGA
AACAGAGCAA TAACAGGTCT GTGATGCCCT TAGATGTCCG GGGCCGCACG CGCGCTACAC
TGAAGTGATC AGCGTGCAGC CTACTCTGCC AAGAGTGGGA AACCCAATGA ACCTTCGTGA
TTGGGATATT GTAATTATTC TCCTTGAACG AGGAATTCCC AGTAAGCGCG AGTCA
>Trhypochthonius_cladonicola
ATCAGTTACG GTTAGATGTT GACATCTACA TGGATAACTG TGGTAATTCT AGAGCTAATA
CATGCACAAA AGCTTCGACC TGGAAGAAGC GCATTTATTA GAACAAGACC AATGGGTGTG
GTGACTCTGG ATAACTGCTA ATCGCATGGC CGTGCCGGCG ATGAATTCAA GTGTCTGCCT
TATCAACTGT CGATGGTAGG TTATGCGCCT ACCATGGTTG TAACGGGTAA CGGGGAATCA
TTCGATTCCA GCCTGAGAAA CTACCACATC CAAGGAAGGC AGCAGGCACG CAAATTACCC
AAGGTAGTGA CGAAAAATAA CAATACGGGA CTCTTATGAG GCCCCGTAAT TGGAATGAGA
ACAATCTAAA TCCTTAACGA GGATCTATTG GAGGGCAAGT CTGGTGCCAG CAGCCGCGGT
AATCCAGCTC CAATAGCGTA TATTAAAGTT GTTGTGGTTA AAAAGCTCGT AGTTGGATCT
CAGTTCGAGT CGGCGGTCCA CTTGCCAGTG GTTACTGCTT TGAACATTAT CGCCTATGGT
GCTCTTTACC GAGTGTCATA GGCGATCGGT ACGTTTACTT TGAAAAAATT AGAGTGCTCA
AAGCAGGCGC CCGAATAATG TTGCATGGAA TAATGGAATA GGATCTCGGT TCTATTTTGT
TGGTCTTCGG AACTGAGGTA ATGATTAGAG GGACAGACGG GGGCATTCGT ATTGCGGCGC
TAGAGGTGAA ATTCTTGGAC CGTCGCAAGA CGAACTAAAG CGAAAGCACC AAGAATGTTT
TCATTAATCA AGAACGAAAG TTAGAGGTTC GAAGGCGATC AGATACCGCC CTAGTTCTAA
CCATAAACGA TGCCAACCAG TAATCCGTCT GAGTTCAAAT GACTCGCGAC TTCCGGGAAA
```

```
CCAAAGTTCG GTTCCAGGGG AAGTATGGTT GCAAAGCTGA AACTTAAAGA AATTGACGGA
AGGGCACCAC CAGGAGTGGA GCCTGCGGCT TAATTTGACT CAACACGGGA AAACTCACCC
GGCCCGGACA CTGGAAGGAT TGACAGATTG AGAGCTCTTT CTTGATTCAG TGGGTGGTGG
TGCATGGCCG TTCTTAGTTG GTGGAGCGAT TTGTCTGGTT AATTCCGATA ACGAACGAGA
CTCTAGCCTA CTAAATAACG CCGACTTCTT AGAGGGACAG GCGGCGATTC AGCCGCACGA
AACAGAGCAA TAACAGGTCT GTGATGCCCT TAGATGTCCG GGGCCGCACG CGCGCTACAC
TGAAGTGATC AGCGTGCAGC CTACTCTGCC AAGAGTGGGA AACCCAATGA ACCTTCGTGA
TTGGGATATT GTAATTATTC CCCTTGAACG AGGAATTCCC AGTAAGCGCG AGTCA
>Eupelops_plicatus
ATCAGTTACG GTTAGATGTT GACATCTACA TGGATAACTG TGGTAATTCT AGAGCTAATA
CATGCACAAA AGCTTTGACC TGGAAAAAGC GCATTTATTA GAACAAGACC AATGGGGGTG
GTGACTCTGG ATAACTGCTA ATCGCATGGC CGTGCCGGCG ATGAATTCAA GTGTCTGCCT
TATCAACTGT CGATGGTAGG TTATGCGCCT ACCATGGTTG TAACGGGTAA CGGGGAATAA
TTCGATTCCA GCCTGAGAAA CTACCACATC CAAGGAAGGC AGCAGGCACG CAAATTACCC
AAGGTAGTGA CGAAAAATAA CAATACGGGA CTCTTATGAG GCCCCGTAAT TGGAATGAGA
ACAATCTAAA TCCTTAACGA GGATCTATTG GAGGGCAAGT CTGGTGCCAG CAGCCGCGGT
AATCCAGCTC CAATAGCGTA TATTAAAGTT GTTGTTGTTA AAAAGCTCGT AGTTGGATCT
CAGTTCGAGT CAGCGGTCCA CTTGCCAGTG GTTACTGTTT TGAACATTAT CGCCTATGGT
GCTCTTCACC GAGTGTCATA GGCGATCGAT ACGTTTACTT TGAAAAAATT AGAGTGCTCA
AAGCAGGCGC CCGAATAATG TTGCATGGAA TAATGGAATA GGACCTCGGT TCTATTTTGT
TGGTCTTCGG AACTGAGGTA ATGATTAGAG GGACAGACGG GGGCATTCGT ATTGCGACGC
TAGAGGTGAA ATTCTTGGAC CGTTGCAAGA CGAACTAAAG CGAAGGCACC AAGAATGTTT
TCATTAATCA AGAACGAAAG TTAGCGGATC GAAGGCGATC AGATACCGCC CTAGTGCTAA
CCATAAACGA TGCCAACCAG TAATAAGCCT GAGTTCAAAT GACTCGGGAC TTCCGGGAAA
CCAAAGTTCG GTTCAAGGGG AAGTATGGTT GCAAAGCTGA AACTTAAAGA AATTGACGGA
AGGGCACCAC CAGGAGTGGA GCCTGCGGCT TAATTTGACT CAACACGGGA AAACTCACCC
GGCCCGGACA CTGGAAGGAT TGACAGATTG AGAGCTCTTT CTTGATTCAG TGGGTGGTGG
TGCATGGCCG TTCTTAGTTG GTGGAGTGAT TTGTCTGGTT AATTCCGATA ACGAACGAGA
CTCTAGCCTA CTAAATAACG CCGACTTCTT AGAGGGACAG GCGGTGATTC AACCGCACGA
AACAGAGCAA TAACAGGTCT GTGATGCCCT TAGATGTCCG GGGCCGCACG CGCGCTACAC
TGAAGTGATC AGCGTGCTGC CTACTCTGTC AAGAGTGGGA AACCCAATGA ACCTTCGTGA
TTGGGATATT GTAATTATTC TCCTTGAACG AGGAATTCCC AGTAAGCGCG AGTCA
>Xenillus_discrepans
ATCAGTTACG GTTAGATGTT GACATTTACA TGGATAACTG TGGTAATTCT AGAGCTAATA
CATGCACAAA AGCTTCGACC TGGAAGAAGC GCATTTATTA GACCAAGACC AATGGGGGTG
GTGACTCTGT ATAACTGCTA ATCGCATGGC CGTGCCGGCG ATGAATTCAA GTGTCTGCCT
TATCAACTGT CGATGGTAGG CTATGCGCCT ACCATGGTTG TAACGGGTAA CGGGGAATCA
TTCGATTCCA GCCTGAGAAA CTACCACATC CAAGGAAGGC AGCAGGCACG CAAATTACCC
AAGGTAGTGA CGAAAAATAA CAATACGGGA CTCTTATGAG GCCCCGTAAT TGGAATGAGA
ACAATCTAAA TCCTTAACGA GGATCTATTG GAGGGCAAGT CTGGTGCCAG CAGCCGCGGT
AATCCAGCTC CAATAGCGTA TATTAAAGTT GTTGTGGTTA AAAAGCTCGT AGTTGGATCT
CAGTTCGAGT CGGTGGTCCA CTTGCCAGTG GTTACTACTT TGAACATTAT CGCCTATGGT
GCTCTTCACC GAGTGTCATA GGCGATCGAT ACGTTTACTT TGAAAAAATT AGAGTGCTCA
AAGCAGGCGC CCGAATAATG TTGCATGGAA TAATGGAATA GGACCTCGGT TCTATTTTGT
TGGTCTTCGG AACTGAGGTA ATGATTAGAG GGACAGACGG GGGCATTCGT ATTGCGGCGC
TAGAGGTGAA ATTCTTGGAC CGTCGCAAGA CGAACTAAAG CGAAAGCACC AAGAATGTTT
TCATTAATCA AGAACGAAAG TTAGAGGTTC GAAGGCGATC AGATACCGCC CTAGTTCTAA
CCATAAACGA TGCCGACCAG TAATTAAGCT GAGTTCAAAT GACTCGCGAC TTCCGGGAAA
CCAAAGTTCG GTTCCAGGGG AAGTATGGTT GCAAAGCTGA AACTTAAAGA AATTGACGGA
AGGGCACCAC CAGGAGTGGA GCCTGCGGCT TAATTTGACT CAACACGGGA AAACTCACCC
GGCCCGGACA CTGGAAGGAT TGACAGATTG AGAGCTCTTT CTTGATTCAG TGGGTGGTGG
TGCATGGCCG TTCTTAGTTG GTGGAGTGAT TTGTCTGGTT AATTCCGATA ACGAACGAGA
CTCTAGCCTA CTAAATAACG CTAACTTCTT AGAGGGACAG GCGGTGATTC AACCGCACGA
AACAGAGCAA TAACAGGTCT GTGATGCCCT TAGATGTCCG GGGCCGCACG CGCGCTACAC
TGAAGTGATC AGCGTGCAGC CTACTCTGTC AAGAGTGGGA AACCCAATGA ACCTTCGTGA
TTGGGATATT GTAATTATTC CCCTTGAACG AGGAATTCCC AGTAAGCGCG AGTCA
>Scutoverte_sculptus
ATCAGTTACG GTTAGATATT GACAACTACA TGGATAACTG TGGTAATTCT AGAGCTAATA
CATGCACAAA AGCTTCGACC TGGCAGAAGC GCATTTATTA GACCAAGACC AATGGGGGTG
GTGACTCTGG ATAACTGCTG ATCGCATGGC CGAGCCGGCG ACGAATTCAA GTGTCTGCCT
```

TATCAACTGT CGATGGTAGG TTATGCGCCT ACCATGGTTG TAACGGGTAA CGGGGAATCA
TTCGATTCCA GCCTGAGAAA CTACCACATC CAAGGAAGGC AGCAGGCACG CAAATTACCC
AAGGTAGTGA CGAAAAATAA CAATACGGGA CTCTTATGAG GCCCCGTAAT TGGAATGAGA
ACAATTTAAA TCCTTAACGA GGATCTATTG GAGGGCAAGT CTGGTGCCAG CAGCCGCGGT
AATCCAGCTC CAATAGCGTA TATTAAAGTT GTTGTGGTTA AAAAGCTCGT AGTTGGATCT
CAGTTCGAGT CGACGGTCCA CTTGCCAGTG GTTACTGTCT TGAACGTTAT CGTCTATGGT
GCTCTTCACC GAGTGTCATA GGCGATCGAT ACGTTTACTT TGAAAAAATT AGAGTGCTCA
AAGCAGGCGC CCGAATAATG TTGCATGGAA TAATGGAATA GGATCTCGGT TCTATTTTGT
TGGTCTTCGG AACTGAGATA ATGATTAGAG GGACAGACGG GGGCATTCGT ATTGCGACGC
TAGAGGTGAA ATTCTTGGAC CGTTGCAAGA CGAACTAAAG CGAAGGCACC AAGAATGTTT
TCATTAATCA AGAACGAAAG TTAGCGGATC GAAGGCGATC AGATACCGCC CTAGTGCTAA
CCATAAACGA TGCCAACCAG TAATAAGCCT GAGTTCAAAT GACTCGGGAC TTCCGGGAAA
CCAAAGTTCG GTTCCAGGGG AAGTATGGTT GCAAAGCTGA AACTTAAAGA AATTGACGGA
AGGGCACCAC CAGGAGTGGA GCCTGCGGCT TAATTTGACT CAACACGGGA AAACTCACCC
GGCCCGGACA CTGGAAGGAT TGACAGATTG AGAGCTCTTT CTTGATTCAG TGGGTGGTGG
TGCATGGCCG TTCTTAGTTG GTGGAGTGAT TTGTCTGGTT AATTCCGATA ACGAACGAGA
CTCTAGCCTA CTAAATAACT CATACTTCTT AGAGGGACAG GCGGTGATTC AACCGCACGA
AACAGAGCAA TAACAGGTCT GTGATGCCCT TAGATGTCCG GGGCCGCACG CGCGCTACAC
TGAAGTGATC AGCGTGCAGC CTACTCTGTC AAGAGTGGGA AACCCAATGA ACCTTCGTGA
TTGGGATATT GTAATTATTC CCCTTGAACG AGGAATTCCC AGTAAGCGCG AGTCA
>Gehypochthonius_urticinus
ATCAGTTACG GTTAGATGTT GACGTCTACA TGGATAACTG TGGTAATTCT AGAGCTAATA
CATGCACAAA AGCTTCGACC TGGAAGGAGC GCATTTATTA GACCAAGACC AATGGGGGTG
GTGACTCTGG ATAACTGCTA ATCGCATGGC CGCGCCGGCG ATGAATTCAA GTGTCTGCCT
TATCAACTGT CGATGGTAGG TTATGCGCCT ACCATGGTTG TAACGGGTAA CGGGGAATCA
TTCGATTCCA GCCTGAGAAA CTACCACATC CAAGGAAGGC AGCAGGCACG CAAATTACCC
ACGGTAGTGA CGAAAAATAA CAATACGGGA CTCTAATGAG GCCCCGTAAT TGGAATGAGA
ACAATCTAAA TCCTTAACGA GGATCTATTG GAGGGCAAGT CTGGTGCCAG CAGCCGCGGT
AATCCAGCTC CAATAGCGTA TATTAAAGTT GTTGCGGTTA AAAAGCTCGT AGTTGGATCT
CAGTTCAAGT TAGCGGTCCA CTTGCCAGTG GTTACTGTTT TGAACATTAC CGCTTATGGT
GCTCTTCACC GAGTGTCATA AGCGATCGGT ACGTTTACTT TGAAAAAATT AGAGTGCTCA
AAGCAGGCGC CCGAATAATG TTGCATGGAA TAATGGAATA GGACCTCGGT TCTATTTTGT
TGGTCTTCGG AACTGAGGTA ATGATTAGAG GGACAGACGG GGGCATTCGT ATTGCGGCGC
TAGAGGTGAA ATTCTTGGAC CGTCGCAAGA CGAACTAAAG CGAAAGCACC AAGAATGTTT
TCATTAATCA AGAACGAAAG TTAGAGGTTC GAAGGCGATC AGATACCGCC CTAGTTCTAA
CCATAAACGA TGCCAACCAG CAATCCGCCT GAGTTCAAAT GACTCGCGGC TTCCGGGAAA
CCAAAGTTCG GTTCCGGGGG AAGTATGGTT GCAAAGCTGA AACTTAAAGG AATTGACGGA
AGGGCACCAC CAGGAGTGGA GCCTGCGGCT TAATTTGACT CAACACGGGA AAACTTACCC
GGCCCGGACA CTGTAAGGAT TGACAGATTG AGAGCTCTTT CTTGATTCAG TGGGTGGTGG
TGCATGGCCG TTCTTAGTTG GTGGAGCGAT TTGGGTGGTT AATTCCGATA ACGAACGAGA
CTCTAGCCTA CTAAATAACG CCAGCTTCTT AGAGGGACAG GCGGCGATTC AGCCGCACGA
AACAGAGCAA TAACAGGTCT GTGATGCCCT TAGATGTCCG GGGCCGCACG CGCGCTACAC
TGAAGTGATC AGCGTGCAGC CTGCTCTGCC AAGAGTGGGT AACCCAATGA ACCTTCGTGA
TTGGGATATT GTAATTATTC CCCTTGAACG AGGAATTCCC AGTAAGCGCG AGTCA
>Lohmannia_banksi
ATCAGTTACG GTTAGATGTT GACGTCTACA TGGATAACTG TGGTAATTCT AGAGCTAATA
CATGCACAAA AGCTTCAACC GGGAAGAAGC GCATTTATTA GACCAAGACC AATGGGAGTG
ATGAATCTGA ATAACTGCTG ATCGCATGGC CGCGCCGGCG ACGAATTCAA GTGTCTGCCT
TATCAACTGT CGATGGTAGG TTATGCGCCT ACCATGGTTG TAACGGGTAA CGGGGAATCA
TTCGATTCCA GCCTGAGAAA CTACCACATC CAAGGAAGGC AGCAGGCACG CAAATTACCC
ACGGTAGTGA CGAAAAATAA CAATACGGGA CTCGAATGAG GCCCCGTAAT TGGAATGAGT
ACAATTTAAA TCCTTAACGA GGATCTATTG GAGGGCAAGT CTGGTGCCAG CAGCCGCGGT
AATCCAGCTC CAATAGCGTA TATTAAAGTT GTTGCGGTTA AAAAGCTCGT AGTTGGATCT
CAGTTTGCGT CGGCGGTCCA CTTGCCAGTG GTCACTGCCT TGAACATTAT CGCTTATGGT
GCTCTTCACC GAGTGTCATA AGCGATCGGT ACGTTTACTT TGAAAAAATT AGAGTGCTCA
AAGCAGGCGC CCGAATAATG TTGCATGGAA TAATGGAATA GGACCTCGGT TCTATTTTGT
TGGTCTTCGG GACTGAGGTA ATGATTAGAG GGACAGACGG GGGCATTCGT ATTGCGACGC
TAGAGGTGAA ATTCTTGGAC CGTCGCAAGA CGAACTAAAG CGAAAGCACC AAGAATGTTT
TCATTAATCA AGAACGAAAG TTAGAGGTTC GAAGGCGATC AGATACCGCC CTAGTTCTAA
CCATAAACGA TGCCAACCAG CAATCCGCCT GAGTTCAAAT GACTCGCGGC TTCCGGGAAA

CCAAAGTTCG GTTCCGGGGG AAGTATGGTT GCAAAGCTGA AACTTAAAGG AATTGACGGA
AGGGCACCAC CAGGAGTGGA GCCTGCGGCT TAATTTGACT CAACTCGGGA AAACTTACCC
GGCCCGGACA CTGGAAGGAT TGACAGATTG AGAGCTCTTT CTTGATTCAG TGGGTGGTGG
TGCATGGCCG TTCTTAGTTG GTGGAGCGAT TTGTCTGGTT AATTCCGATA ACGAACGAGA
CTCTAGCCTA CTAAATAACG TCTGCTTCTT AGAGGGACAG GCGGCGATTC AGCCGCACGA
AACAGAGCAA TAACAGGTCT GTGATGCCCT TAGATGTCCG GGGCCGCACG CGCGCTACAC
TGAAGTGATC AGCGTGCACC CTGCTCTGCC AAGAGTGGGT AACCCAATGA ACCTTCGTGA
TTGGGATATT GTAATTATTC CCCTTGAACG AGGAATTCCC AGTAAGCGCG AGTCA
>Trichoribates_trimaculatus
ATCAGTTACG GTTAGATGTT GACATCTACA TGGATAACTG TGGTAATTCT AGAGCTAATA
CATGCACAAA AGCTTCGACC TGGAAGAAGC GCATTTATTA GAACAAGACC AATGGGGGTG
GTGACTCTGA ATAACTGCTA ATCGCATGGC CGTGCCGGCG ATGAATTCAA GTGTCTGCCT
TATCAACTGT CGATGGTAGG TTATGCGCCT ACCATGGTTG TAACGGGTAA CGGGGAATTA
TTCGATTCCG GCCTGAGAAA CCAACACATC CAAGGAAGGC AGCAGGCACG CAAATTACCC
AAGGTAGTGA CGAAAAATAA CAATACGGGA CTCTTATGAG GCCCCGTAAT TGGAATGAGA
ACAATCTAAA TCCTTAACGA GGATCTATTG GAGGGCAAGT CTGGTGCCAG CAGCCGCGGT
AATCCAGCTC CAATAGCGTA TATTAAAGTT GTTGTTGTTA AAAAGCTCGT AGTTGGATCT
CAGTTCGAGT CAGCGGTCCA CTTGCCAGTG GTTACTGTTT TGAACATTAT CGCCTATGGT
GCTCTTCACC GAGTGTCATA GGCGATCGAT ACGTTTACTT TGAAAAAATT AGAGTGCTCA
AAGCAGGCGC CCGAATAATG TTGCATGGAA TAATGGAATA AGACCTCGGT TCTATTTTGT
TGGTCTTCGG AACTGAGGTA ATGATTAGAG GGACAGACGG GGGCATTCGT ATTGCGACGC
TAGAGGTGAA ATTCTTGGAC CGTTGCAAGA CGAACTAAAG CGAAGGCACC AAGAATGTTT
TCATTAATCA AGAACGAAAG TTAGCGGATC GAAGGCGATC AGATACCGCC CTAGTGCTAA
CCATAAACGA TGCCAACCAG TAATAAGCCT GAGTTCAAAT GACTCGGGAC TTCCGGGAAA
CCAAAGTTCG GTTCAAGGGG AAGTATGGTT GCAAAGCTGA AACTTAAAGA AATTGACGGA
AGGGCACCAC CAGGAGTGGA GCCTGCGGCT TAATTTGACT CAACACGGGA AAACTCACCC
GGCCCGGACA CTGGAAGGAT TGACAGATTG AGAGCTCTTT CTTGATTCAG TGGGTGGTGG
TGCATGGCCG TTCTTAGTTG GTGGAGTGAT TTGTCTGGTT AATTCCGATA ACGAACGAGA
CTCTAGCCTA CTAAATAACG CCAACTTCTT AGAGGGACAG GCGGTGATTC AACCGCACGA
AACAGAGCAA TAACAGGTCT GTGATGCCCT TAGATGTCCG GGGCCGCACG CGCGCTACAC
TGAAGTGATC AGCGTGCTGC CTACTCTGTC AAGAGTGGGA AACCCAATGA ACCTTCGTGA
TTGGGATATT GTAATTATTC TCCTTGAACG AGGAATTCCC AGTAAGCGCG AGTCA
>Eohypochthonius_sp
ATCAGTTACG GTTAGATGTT GACGTCTACA TGGATAACTG TGGTAATTCT AGAGCTAATA
CATGCACAAA AGCTTCGACC GGGAAGAAGC GCATTTATTA GACCAAGACC AATGGGGGTG
ATGAATCTGA ATAACTGCTG ATCGCATGGC CGCGCCGGCG ACGAATTCAA GTGTCTGCCT
TATCAACTGT CGATGGTAGG TTATGCGCCT ACCATGGTTG TAACGGGTAA CGGGGAATCA
TTCGATTCCA GCCTGAGAAA CTACCACATC CAAGGAAGGC AGCAGGCACG CAAATTACCC
ACGGTAGTGA CGAAAAATAA CAATACGGGA CTCTAATGAG GCCCCGTAAT TGGAATGAGA
ACAATCTAAA TCCTTAACGA GGATCTATTG GAGGGCAAGT CTGGTGCCAG CAGCCGCGGT
AATCCAGCTC CAATAGCGTA TATTAAAGTT GTTGCGGTTA AAAAGCTCGT AGTTGGATCT
CAGTTCGCGT CGACGGTCCA CTTGCCAGTG GTTACTGTCT TGAACATTAC CGCTTATGGT
GCTCTTCGCC GAGTGTCATA AGCGATCGGT ACGTTTACTT TGAAAAAATT AGAGTGCTCA
AAGCAGGCGC CCGAATAATG TTGCATGGAA TAATGGAATA GGACCTCGGT TCTATTTTGT
TGGTCTTCGG AACCGAGGTA ATGATTAGAG GGACAGACGG GGGCATTCGT ATTGCGGCGC
TAGAGGTGAA ATTCTTGGAC CGTCGCAAGA CGAACTAAAG CGAAAGCACC AAGAATGTTT
TCATTAATCA AGAACGAAAG TTAGAGGTTC GAAGGCGATC AGATACCGCC CTAGTTCTAA
CCATAAACGA TGCCAACCAG CAATCCGCCT GAGTTCAAAT GACTCGCGGC TTCCGGGAAA
CCAAAGTTCG GTTCCGGGGG AAGTATGGTT GCAAAGCTGA AACTTAAAGG AATTGACGGA
AGGGCACCAC CAGGAGTGGA GCCTGCGGCT TAATTTGACT CAACACGGGA AAACTTACCC
GGCCCGGACA CTGGAAGGAT TGACAGATTG AGAGCTCTTT CTTGATTCAG TGGGTGGTGG
TGCATGGCCG TTCTTAGTTG GTGGAGCGAT TTGTCTGGTT AATTCCGATA ACGAACGAGA
CTCTAGCCTA CTAAATAACG TCAGCTTCTT AGAGGGACAG GCGGCGGTTC AGCCGCACGA
AACAGAGCAA TAACAGGTCT GTGATGCCCT TAGATGTCCG GGGCCGCACG CGCGCTACAC
TGAAGTGATC AGCGTGCAAC CTGGTCGGCC ACGACTGGGT AACCCAATGA ACCTTCGTGA
TTGGGATATT GTAATTATTC CCCTTGAACG AGGAATTCCC AGTAAGCGCG AGTCA
>Gymnodamaeus_bicostatus
ATCAGTTACG GTTAGATGTT GACATCTACA TGGATAACTG TGGTAATTCT AGAGCTAATA
CATGCACAAA AGCTTCGACC TGGAAGAAGC GCATTTATTA GACCAAGACC AATGGGGGTG
GTGACTCTGG ATAACTGCTA ATCGCATGGC CGAGCCGGCG ATGAATTCAA GTGTCTGCCT

TATCAACTGT CGATGGTAGG TTATGCGCCT ACCATGGTTG TAACGGGTAA CGGGGAATCA
TTCGATTCCA GCCTGAGAAA CTACCACATC CAAGGAAGGC AGCAGGCACG CAAATTACCC
AAGGTAGTGA CGAAAAATAA CAATACGGGA CTCTTATGAG GCCCCGTAAT TGGAATGAGA
ACAATCTAAA TCCTTAACGA GGATCTATTG GAGGGCAAGT CTGGTGCCAG CAGCCGCGGT
AATCCAGCTC CAATAGCGTA TATTAAAGTT GTTGTTGTTA AAAAGCTCGT AGTTGGATCT
CAGTTCGAGT CGGCGGTCCA CTTGCCAGTG GCTACTGCTT TGAACATTAT CGGGTATGGT
GCTCTTCACC GAGTGTCATA TCTGACCGAT ACGTTTACTT TGAAAAAATT AGAGTGCTCA
AAGCAGGCGC CCGAATAATG TTGCATGGAA TAATGGAATA GGACCTCGGT TCTATTTTGT
TGGTCTTCGG AACTGAGGTA ATGATTAGAG GGACAGACGG GGGCATTCGT ATTGCGGCGC
TAGAGGTGAA ATTCTTGGAC CGTCGCAAGA CGAACTAAAG CGAAAGCACC AAGAATGTTT
TCATTAATCA AGAACGAAAG TTAGAGGTTC GAAGGCGATC AGATACCGCC CTAGTTCTAA
CCATAAACGA TGCCAACCAG TACTTCGTCT GAGTTCAAAT GACTCGTGAC TTCCGGGAAA
CCAAAGTTCG GTTCAAGGGG AAGTATGGTT GCAAAGCTGA AACTTAAAGA AATTGACGGA
AGGGCACCAC CAGGAGTGGA GCCTGCGGCT TAATTTGACT CAACACGGGA AAACTCACCC
GGCCCGGACA CTGGAAGGAT TGACAGATTG AGAGCTCTTT CTTGATTCAG TGGGTGGTGG
TGCATGGCCG TTCTTAGTTG GTGGAGCGAT TTGTCTGGTT AATTCCGATA ACGAACGAGA
CTCTAGCCTA CTAAATAACG CCGACTTCTT AGAGGGACAG GCGGTGATTC AACCGCACGA
AACAGAGCAA TAACAGGTCT GTGATGCCCT TAGATGTCCG GGGCCGCACG CGCGCTACAC
TGAAGTGATC AGCGTGCAGC CTACTCTGTC AAGAGTGGGA AACCCAATGA ACCTTCGTGA
TTGGGATATT GTAATTATTC CCCTTGAACG AGGAATTCCC AGTAAGCGCG AGTCA
>Camisia_spinifer
ATCAGTTACG GTTAGATGTT GACATCTACA TGGATAACTG TGGTAATTCT AGAGCTAATA
CATGCACAAA AGCTTCGACC TGGAAGAAGC GCATTTATTA GACCAAGACC AATGGGGGTG
GTGACTCTGG ATAACTGCTA ATCGCATGGC CGTGCCGGCG ATGAATTCAA GTGTCTGCCT
TATCAACTGT CGATGGTAGG TTATGCGCCT ACCATGGTTG TAACGGGTAA CGGGGAATCA
TTCGATTCCA GCCTGAGAAA CTACCACATC CAAGGAAGGC AGCAGGCACG CAAATTACCC
AAGGTAGTGA CGAAAAATAA CAATACGGGA CTCTTATGAG GCCCCGTAAT TGGAATGAGA
ACAATTTAAA TCCTTAACGA GGATCTATTG GAGGGCAAGT CTGGTGCCAG CAGCCGCGGT
AATCCAGCTC CAATAGCGTA TATTAAAGTT GTTGTGGTTA AAAAGCTCGT AGTTGGATCT
CAGTTCGAGT CGGCGGTCCA CTTGCCAGTG GTTACTGCTT TGAACATTAT CGCCTATGGT
GCTCTTCACC GAGTGTCATA GGCGATCGGT ACGTTTACTT TGAAAAAATT AGAGTGCTCA
AAGCAGGCGC CCGAATAATG TTGCATGGAA TAATGGAATA GGACCTTGGT TCTATTTTGT
TGGTCTTCGG AACTAAGGTA ATGATTAGAG GGACAGACGG GGGCATTCGT ATTGCGGCGC
TAGAGGTGAA ATTCTTGGAC CGTCGCAAGA CGAACTAAAG CGAAAGCACC AAGAATGTTT
TCATTAATCA AGAACGAAAG TTAGAGGTTC GAAGGCGATC AGATACCGCC CTAGTTCTAA
CCATAAACGA TGCCAACCAG TAATCCGTCT GAGTTCAAAT GACTCGCGAC TTCCGGGAAA
CCAAAGTTCG GTTCCAGGGG TAGTATGGTT GCAAAGCTGA AACTTAAAGA AATTGACGGA
AGGGCACCAC CAGGAGTGGA GCCTGCGGCT TAATTTGACT CAACACGGGA AAACTCACCC
GGCCCGGACA CTGGAAGGAT TGACAGATTG AGAGCTCTTT CTTGATTCAG TGGGTGGTGG
TGCATGGCCG TTCTTAGTTG GTGGAGCGAT TTGTCTGGTT AATTCCGATA ACGAACGAGA
CTCTAGCCTA CTAAATAACG CCGACTTCTT AGAGGGACAG GCGGCGATTC AGCCGCACGA
AACAGAGCAA TAACAGGTCT GTGATGCCCT TAGATGTCCG GGGCCGCACG CGCGCTACAC
TGAAGTGATC AGCGTGCAGC CTACTCTGCC AAGAGTGGGA AACCCAATGA ACCTTCGTGA
TTGGGATATT GTAATTATTC TCCTTGAACG AGGAATTCCC AGTAAGCGCG AGTCA
>Heminothrus_paolianus
ATCAGTTACG GTTAGATGTT GACATCTACA TGGATAACTG TGGTAATTCT AGAGCTAATA
CATGCACAAA AGCTTCGACC TGGAAGAAGC GCATTTATTA GACCAAGACC AATGGGGGTG
GTGACTCTGG ATAACTGCTA ATCGCATGGC CGTGCCGGCG ATGAATTCAA GTGTCTGCCT
TATCAACTGT CGATGGTAGG TTATGCGCCT ACCATGGTTG TAACGGGTAA CGGGGAATCA
TTCGGTTCCA GCCTGAGAAA CTACCACATC CAAGGAAGGC AGCAGGCACG CAAATTACCC
AAGGTAGTGA CGAAAAATAA CAATACGGGA CTCTTATGAG GCCCCGTAAT TGGAATGAGA
ACAATCTAAA TCCTTAACGA GGATCTATTG GAGGGCAAGT CTGGTGCCAG CAGCCGCGGT
AATCCAGCTC CAATAGCGTA TATTAAAGTT GTTGTGGTTA AAAAGCTCGT AGTTGGATCT
CAGTTCGAGT CGGCGGTCCA CTTGCCAGTG GTTACTGCTT TGAACATTAT CGCCTATGGT
GCTCTTTACC GAGTGTCATA GGCGATCGGT ACGTTTACTT TGAAAAAATT AGAGTGCTCA
AAGCAGGCGC CCGAATAATG TTGCATGGAA TAATGGAATA GGACCTCGGT TCTATTTTGT
TGGTCTTCGG AACTGAGGTA ATGATTAGAG GGACAGACGG GGGCATTCGT ATTGCGGCGC
TAGAGGTGAA ATTCTTGGAC CGTCGCAAGA CGAACTAAAG CGAAAGCACC AAGAATGTTT
TCATTAATCA AGAACGAAAG TTAGAGGTTC GAAGGCGATC AGATACCGCC CTAGTTCTAA
CCATAAACGA TGCCAACCAG TAATCCGTCT GAGTTCAAAT GACTCGCGAC TTCCGGGAAA

CCAAAGTTCG GTTCCAGGGG AAGTATGGTT GCAAAGCTGA AACTTAAAGA AATTGACGGA
AGGGCACCAC CAGGAGTGGA GCCTGCGGCT TAATTTGACT CAACACGGGA AAACTCACCC
GGCCCGGACA CTGGAAGGAT TGACAGATTG AGAGCTCTTT CTTGATTCAG TGGGTGGTGG
TGCATGGCCG TTCTTAGTTG GTGGAGCGAT TTGTCTGGTT AATTCCGATA ACGAACGAGA
CTCTAGCCTA CTAAATAACG CCAACTTCTT AGAGGGACAG GCGGCGATTC AGCCGCACGA
AACAGAGCAA TAACAGGTCT GTGATGCCCT TAGATGTCCG GGGCCGCACG CGCGCTACAC
TGAAGTGATC AGCGTGCAGC CTACTCTGCC AAGAGTGGGA AACCCAATGA ACCTTCGTGA
TTGGGATATT GTAATTATTC TCCTTGAACG AGGAATTCCC AGTAAGCGCG AGTCA
>Liodes_sp
ATCAGTTACG GTTAGATGTT GACATCTACA TGGATAACTG TGGTAATTCT AGAGCTAATA
CATGCACAAA AGCTTCGACC TGGAAGAAGC GCATTTATTA GACCAAGACC AATGGGGGTG
GTGACTCTGG ATAACTGCTA ATCGCATGGC CGAGCCGGCG ATGAATTCAA GTGTCTGCCT
TATCAACTGT CGATGGTAGG TTATGTGCCT ACCATGGTTG TAACGGGTAA CGGGGAATCA
TTCGATTCCA GCCTGAGAAA CTACCACATC CAAGGAAGGC AGCAGGCACG CAAATTACCC
AAGGTAGTGA CGAAAAATAA CAATACGGGA CTCTTTTGAG GCCCCGTAAT TGGAATGAGA
ACAATCTAAA TCCTTAACGA GGATCTATTG GAGGGCAAGT CTGGTGCCAG CAGCCGCGGT
AATCCAGCTC CAATAGCGTA TATTAAAGTT GTTGTGGTTA AAAAGCTCGT AGTTGGATCT
CAGTTCGAGT CGGCGGTCCA CTTGCCAGTG GTTACTGCTT TGAACATTAT CGCCTATGGT
GCTCTTCACC GAGTGTCATA GGTGATCGGT ACGTTTACTT TGAAAAAATT AGAGTGCTCA
AAGCAGGCGC CCGAATAATG TTGCATGGAA TAATGGAATA GGACCTCGGT TCTATTTTGT
TGGTCTTCGG AACTGAGGTA ATGATTAGAG GGACAGACGG GGGCATTCGT ATTGCGGCGC
TAGAGGTGAA ATTCTTGGAC CGTCGCAAGA CGAACTAAAG CGAAAGCACC AAGAATGTTT
TCATTAATCA AGAACGAAAG TTAGAGGTTC GAAGGCGATC AGATACCGCC CTAGTTCTAA
CCATAAACGA TGCCAACCAG TAATATGCCT GAGTTCAAAT GACTCGTGAC TTCCGGGAAA
CCAAAGTTCG GTTCCAGGGG AAGTATGGTT GCAAAGCTGA AACTTAAAGA AATTGACGGA
AGGGCACCAC CAGGAGTGGA GCCTGCGGCT TAATTTGACT CAACACGGGA AAACTCACCC
GGCCCGGACA CCAGGAGGAT TGACAGATTG AGAGCTCTTT CTTGATTTGG TGGGTGGTGG
TGCATGGCCG TTCTTAGTTG GTGGAGCGAT TTGTCTGGTT AATTCCGATA ACGAACGAGA
CTCTAGCCTA CTAAATAACG CCGACTTCTT AGAGGGACAG GCGGTGATTC AACCGCACGA
AACAGAGCAA TAACAGGTCT GTGATGCCCT TAGATGTCCG GGGCCGCACG CGCGCTACAC
TGAAGTGATC AGCGTGCAGC CTACTCTGCC AAGAGTGGGA AACCCAATGA ACCTTCGTGA
TTGGGATATT GTAATTATTC CCCTTGAACG AGGAATTCCC AGTAAGCGCG AGTCA
>Schusteria_littorea
ATCAGTTACG GTTAGATGTT GACATCTACA TGGATAACTG TGGTAATTCT AGAGCTAATA
CATGCACAAA AGCTTCGACC TGGAAGAGGC GCATTTATTA GACCAAGACC AATGGGGATG
GTGACTCTGG ATAACTGCTA ATCGCATGGC CGAGCCGGCG ATGAATTCAA GTGTCTGCCT
TATCAACTGT CGATGGTAAG TTATGCGCTT ACCATGGTTG TAACGGGTAA CGGGGAATCA
TTCGATTCCA GCCTTAAAAA CTACCACATC CAAGGAAGGC AGCAGGCACG CAAATTACCC
AAGGTAGTGA CGAAAAATAA CAATACGAGA CTCTTATGAG GCCTCGTAAT TGGAATGAGA
ACAATTTAAA TCCTTAACGA GGATCTATTG GAGGGCAAGT CTGGTGCCAG CAGCCGCGGT
AATCCAGCTC CAATAGCGTA TACTAAAGTT GTTGTTGTTA AAAAGCTCGT AGTTGGATCT
CAGTTCTAGT TGATGGTCCA CTTTGAAGTG GCTACTGTCT TGAACAGTAT CGCCTATGGT
GCTCTTCACC GAGTGTCATA GGCGATCGAT AAGTTTACTT TGAAAAAATT AGAGTGCTCA
AAGCAGGCGC CCGAATAATG TTGCATGGAA TAATGGAATA GGACCTCGGT TCTATTTTGT
TGGTCTTCGG AACTGAGGTA ATGATTAGAG GGACAGACGG GGGCATTTGT ATTGCAGCGC
TAGAGGTGAA ATTCTTGGAC CGCTGCAAGA CGAACTAAAG CGAAAGCACC AAGAATGTTT
TCATTAATCA AGAACGAAAG TTAGAGGCTC GAAGGCGATC AGATACCGCC CTAGTTCTAA
CCATAAACGA TGCCAACCAG TAATAGGTCT GAGTTCAAAT GACTCGCGAC TTCCGGGAAA
CCAAAGTTCG GTTCAAGGGG AAGTATGGTT GCAAAGCTGA AACTTAAAAA AATTGACGGA
AGGGCACCAC CAGGAGTGGA GCCTGCGGCT TAATTTGACT CAACACGGGA AAACTCACCC
GGCCCGGACA CTGGAAGGAT TGACAGATTG AGAGCTCTTT CTTGATTCAG TGGGTGGTGG
TGCATGGCCG TTCTTAGTTG GTGGAGTGAT TTGTCTGGTT AATTCCGATA ACGAACGAGA
CTCTAGCCTA CTAAATAACG TCAACTTCTT AGAGGGACAG GCGGTGATTC AACCGCACGA
AACAGAGCAA TAACAGGTCT GTGATGCCCT TAGATGTCCG GGGCCGCACG CGCGCTACAC
TGAAGTGATC AGCGTGCGGC CTACTCTGTC AAGAGTGGGA AACCCAATGA ACCTTCGTGA
TTGGGATATT GTAATTATTG CCCTTGAACG AGGAATTCCC AGTAAGCGCG AGTCA
>Zachvatkinella_sp
ATCAGTTACG GTTAGATATT GACATCTACA TGGATAACTG TGGTAATTCT AGAGCTAATA
CATGCACTAA AGCATCGACC CGGAAGATGT GCATTTATTA GACCAAGACC AATGGAGGTG
GTGACTCTGG ATAACTGCTA ATCGCATGAC CGTGTCGGCG ATGAATTCAA GTGTCTGCCT

TATCAACTGT CGATGGTAGA TTATGCGTCT ACCATGGTTG TAACGGGTAA CGGGGAATCA
TTCGATTCCA GCCTGAGAAA CTACCACATC CAAGGAAGGC AGCAGGCACG CAAATTACCC
AAGGTAGTGA CGAAAAATAA CAATACAGCA CTCTAATGAG GCGCTGTAAT TGGAATGAGA
ACAATCTAAA TCCTTAACGA GGATCTATTG GAGGGCAAGT CTGGTGCCAG CAGCCGCGGT
AATCCAGCTC CAATAGCGTA TATTAAAGTT GTTGCGGTTA AAAAGCTCGT AGTTGGATCT
CAGTTCAAGC CGCAGGTCCA CTTGCCAGTG GTTACTTGTT TGAACATTAC CGCCTATGGT
GCTTTTAAAC GAGTGTCATA GGCGATCGGT ACGTTTACTT TGAAAAAATT AGAGTGCTTA
AAGCAGGCGC CTGAATATTG TTGCATGGAA TAATGGAATA TGTAGGTAAT TCTGTTCTGT
TGGTCTTCGG AAATTCCTAA ATGGTTACAG GGACAGACGG GGGCGTTCGT ATTGCGGCGC
TAGAGGTGAA ATTCTTGGAC CGTCGCAAGA CGAACTAAAG CGATAGCACC AAGAATGTTT
TCATTAATCA AGAACGAAAG TTAGAGGTTC GAAGGCGATC AGATACCGCC CTAGTTCTAA
CCATAAACGA TGCCAACCAG CAATTCGCTT GAGTTCAAAT GACTCGCGGC TTCCGTGAAA
ACAAAGTTCG GTTCCGGGGG AAGTATGGTT GCAAAGCTGA AACTTAAAGG AATTGACGGA
AGGGCACCAC CAGGAGTGGA GCCTGCGGCT TAATTTGACT CAACACGGGA AAACTTACCC
GGCCCGGACA CTGGGAGGAT TGACAGATTG AGAGCTCTTT CTTGATTCAG TGGGTGGTGG
TGCATGGCCG TTCTTAGTTG GTGGAGCGAT TTGTCTGGTT AATTCCGATA ACGAACGAGA
CTCTAGCCTA CTAAATAACG TCGACTTCTT AGAGGGACAG GCGGTGATTC AACCGCACGA
AACAGAGCAA TAACAGGTCT GTGATGCCCT TAGATGTCCG GGGCCGCACG CGCGCTACAC
TGAAGTGATC AGCGTGCTTC CTGCTCCGTC AGGAGTGGGT AACCCAATGA ACCTTCGTGA
TTGGGATTTT GTAATTATTC TCCATGAACG AGGAATTCCC AGTAAGCGCA AGTCA
>Eueremaeus_oblongus
ATCAGTTACG GTTAGATGTT GACATCTACA TGGATAACTG TGGTAATTCT AGAGCTAATA
CATGCACAAA AGCTTCGACC TGGAAGAAGC GCATTTATTA GAACAAGACC AACGGGGGTG
GTGACTCTGG ATAACTGCTA ATCGCATGGC CGAGCCGGCG ATGAATTCAA GTGTCTGCCT
TATCAACTGT CGATGGTAGG TTATGCGCCT ACCATGGTTG TAACGGGTAA CGGGGAATTA
TTCGATTCCA GCCTGAGAAA CTACCACATC CAAGGAAGGC AGCAGGCACG CAAATTACCC
AAGGTAGTGA CGAAAAATAA CAATACGGGA CTCTTATGAG GCCCCGTAAT TGGAATGAGA
ACAATCTAAA TCCTTAACGA GGATCTATTG GAGGGCAAGT CTGGTGCCAG CAGCCGCGGT
AATCCAGCTC CAATAGCGTA TATTAAAGTT GTTGTGGTTA AAAAGCTCGT AGTTGGATCT
CAGTTCGAGT CGGCGGTCCA CTTGCCAGTG GTTACTGCTT TGAACATTAT CGCCTATGGT
GCTCTTCACC GAGTGTCATA GGCGATCGGT ACGTTTACTT TGAAAAAATT AGAGTGCTCA
AAGCAGGCGC CCGAATAATG TTGCATGGAA TAATGGAATA GGACCTCGGT TCTATTTTGT
TGGTCTTCGG AACTGAGGTA ATGATTAGAG GGACAGACGG GGGCATTCGT ATTGCGGCGC
TAGAGGTGAA ATTCTTGGAC CGCTGCAAGA CGAACTAAAG CGAAAGCACC AAGAATGTTT
TCATTAATCA AGAACGAAAG TTAGAGGTTC GAAGGCGATC AGATACCGCC CTAGTTCTAA
CCATAAACGA TGCCAACCAG TAATAAGCCT GAGTTCAAAT GACTCGCGAC TTCCGGGAAA
CCAAAGTTCG GTTCCAGGGG AAGTATGGTT GCAAAGCTGA AACTTAAAGA AATTGACGGA
AGGGCACCAC CAGGAGTGGA GCCTGCGGCT TAATTTGACT CAACACGGGA AAACTCACCC
GGCCCGGACA CTGGAAGGAT TGACAGATTG AGAGCTCTTT CTTGATTCAG TGGGTGGTGG
TGCATGGCCG TTCTTAGTTG GTGGAGCGAT TTGTCTGGTT AATTCCGATA ACGAACGAGA
CTCTAGCCTA CTAAATAACG CCGACTTCTT AGAGGGACAG GCGGCGATTC AGCCGCACGA
AACAGAGCAA TAACAGGTCT GTGATGCCCT TAGATGTCCG GGGCCGCACG CGCGCTACAC
TGAAGTGATC AGCGTGCAGC CTACTCTGTC AAGAGTGGGA AACCCAATGA ACCTTCGTGA
TTGGGATATT GTAATTATTC CCCTTGAACG AGGAATTCCC AGTAAGCGCG AGTCA
>Pseudotocepheus_amonstruosus
ATCAGTTACG GTTAGATATT GACTTTTACA TGGATAACTG TGGTAATTCT AGAGCTAATA
CATGCACAAA AGCTTCGACC TGGAAGAAGC GCATTTATTA GACCAAGACC AATGGGGGTG
GTGACTCTGA ATAACTGCTA ATCGCATGGC CGCGCCGGCG ATGAATTCAA GTGTCTGCCT
TATCAACTTT CGATGGTAGG TTATGCGCCT ACCATGGTTG TAACGGGTAA CGGGGAATTA
TTCGGTTCCA GCCTGAGAAA CTACCACATC CAAGGAAGGC AGCAGGCACG CAAATTACCC
AAGGTAGTGA CGAAAAATAA CAATACAGGA CTCTTATGAG GCCCTGTAAT TGGAATGAGA
ACAATTTAAA TCCTTATCGA GGATCTATTG GAGGGCAAGT CTGGTGCCAG CAGCCGCGGT
AATCCAGCTC CAATAGCGTA TATTAAAGTT GTTGTGGTTA AAAAGCTCGT AGTTGGATCT
CAGTTCGAGT CGATGGTCCA CTTGCCAGTG GTTACTATCT TGAACATTAC CGCCTATGGT
GCTCTTCACC GAGTGTCATA GGCGATCGGT ACGTTTACTT TGAAAAAATT AGAGTGCTCA
AAGCAGGCGC CCGAATAATG TTGCATGGAA TAATGGAATA GGACCTCGGT TCTATTTTGT
TGGTCTTCGG AACTGAGGTA ATGATTAGAG GGACAGACGG GGGCATTCGT ATTGCGGCGC
TAGAGGTGAA ATTCTTGGAC CGTCGCAAGA CGAACTAAAG CGAAAGCACC AAGAATGTTT
TCATTAATCA AGAACGAAAG TTAGAGGTTC GAAGGCGATC AGATACCGCC CTAGTTCTAA
CCATAAACGA TGCCAACCAG TAATTCGCCT GAGTTCTAAT GACTCGGGAC TTCCGGGAAA

```
CCAAAGTTCG GTTCCAGGGG AAGTATGGTT GCAAAGCTGA AACTTAAAGA AATTGACGGA
AGGGCACCAC CAGGAGTGGA GCCTGCGGCT TAATTTGACT CAACACGGGA AAACTCACCC
GGCCCGGACA CTGGAAGGAT TGACAGATTG AGAGCTCTTT CTTGATTCAG TGGGTGGTGG
TGCATGGCCG TTCTTAGTTG GTGGAGCGAT TTGTCTGGTT AATTCCGATA ACGAACGAGA
CTCTAGCCTA CTAAATAACG CTAGCTTCTT AGAGGGACAG GCGGTGATTC AACCGCACGA
AACAGAGCAA TAACAGGTCT GTGATGCCCT TAGATGTCCG GGGCCGCACG CGCGCTACAC
TGAAGTAATC AGCGTGCGTC CTACTCTGTC AAGAGTGGGT AACCCAATGA ACCTTCGTGA
TTGGGATTTT GTAATTTTTC CACATGAACG AGGAATTCCC AGTAAGCGCG AGTCA
>Odontocepheus_oblongus
ATCAGTTACG GTTAGATATT GACTTTTACA TGGATAACTG TGGTAATTCT AGAGCTAATA
CATGCAGAAA AGCTTCGACC AGGGAGAAGC GCATTTATTA GACCAAGACC AATGGGGGTG
GTGACTCTGG ATAACTGCTA ATCGCATGGC CGAGCCGGCG ATGAATTCAA GTGTCTGCCT
TATCAACTGT CGATGGTAGG TTATGCGCCT ACCATGGTTG TAACGGGTAA CGGGGAATTA
TTCGATTCCA GCCTGAGAAA CTACCACATC CAAGGAAGGC AGCAGGCACG CAAATTACCC
AAGGTAGTGA CGAAAAATAA CAATACAGGA CTCTTATGAG GCCCTGTAAT TGGAATGAGA
ACAATTTAAA TCCTTATCGA GGATCTATTG GAGGGCAAGT CTGGTGCCAG CAGCCGCGGT
AATCCAGCTC CAATAGCGTA TATTAAAGTT GTTGTGGTTA AAAAGCTCGT AGTTGGATCT
CAGTTCGAGT CAGCGGTCCA CTTGCCAGTG GTTACTGCTT GGAACATTAC CGCCTATGGT
GCTCTTCACC GAGTGTCATA GGCGATCGGT ACGTTTACTT TGAAAAAATT AGAGTGCTCA
AAGCAGGCGC CCGAATAATG TTGCATGGAA TAATGGAATA GGACCTCGGT TCTATTTTGT
TGGTCTTCGG AACTGAGGTA ATGATTAGAG GGACAGACGG GGGCATTCGT ATTGCGGCGC
TAGAGGTGAA ATTCTTGGAC CGCTGCAAGA CGAACTAAAG CGAAAGCACC AAGAATGTTT
TCATTAATCA AGAACGAAAG TTAGAGGTTC GAAGGCGATC AGATACCGCC CTAGTTCTAA
CCATAAACGA TGCCAACCAG TAATTCGCCT GAGTTCTAAT GACTCGGGAC TTCCGGGAAA
CCAAAGTTCG GTTCCAGGGG AAGTATGGTT GCAAAGCTGA AACTTAAAGA AATTGACGGA
AGGGCACCAC CAGGAGTGGA GCCTGCGGCT TAATTTGACT CAACACGGGA AAACTCACCC
GGCCCGGACA CTGGAAGGAT TGACAGATTG AGAGCTCTTT CTTGATTCAG TGGGTGGTGG
TGCATGGCCG TTCTTAGTTG GTGGAGCGAT TTGTCTGGTT AATTCCGATA ACGAACGAGA
CTCTAGCCTA CTAAATAACG CTGGCTTCTT AGAGGGACAG GCGGTGATTT AACCGCACGA
AACAGAGCAA TAACAGGTCT GTGATGCCCT TAGATGTCCG GGGCCGCACG CGCGCTACAC
TGAAGTGATC AGCGTGCTGC CTACTCTGTC AAGAGTGGGT AACCCAATGA ACCTTCGTGA
TTGGGATTTT GTAATTTTTC CACATGAACG AGGAATTCCC AGTAAGCGCG AGTCA
>DdR2_3
ATCAGTTACG GTTAGATATT GACTTTTACA TGGATAACTG TGGTAATTCT AGAGCTAATA
CATGCCTACA AGCTTCAACC TGGGAGAAGT GCATTTATTA GAACAAGACC AATGGGGGTG
GTGACTCTGG ATAACTGCTA ATCGCATGGC CGTGCCGGCG ATGAATTCAA GTGTCTGCCT
TATCAACTGT CGATGGTAGG TTATGCGCCT ACCATGGTTG TAACGGGTAA CGGGGAATTA
TTCGATTCCA GCCTGAGAAA CTACCACATC CAAGGAAGGC AGCAGGCACG CAAATTACCC
AAGGTAGTGA CGAAAAATAA CAATACAGGA CTCTTATGAG GCCCTGTAAT TGGAATGAGA
ACAATTTAAA TCCTTAACGA GGATCTATTG GAGGGCAAGT CTGGTGCCAG CAGCCGCGGT
AATCCAGCTC CAATAGCGTA TATTAAAGTT GTTGTGGTTA AAAAGCTCGT AGTTGGATCT
CAGTTCGAGT CGACGGTCCA CTTGCCAGTG GTTACTGTTT TGAACATTAC CGCCTATGGT
GCTCTTCACC GAGTGTCATA GGCGATCGGT ACGTTTACTT TGAAAAAATT AGAGTGCTCA
AAGCAGGCGC CCGAATAATG TTGCATGGAA TAATAGAATA GGACCTCGGT TCTATTTTGT
TGGTCTTCGG AACTGAGGTA ATGATTAGAG GGACAGACGG GGGCATTCGT ATTGCGGCGC
TAGAGGTGAA ATTCTTGGAC CGTCGCAAGA CGAACTAAAG CGAAAGCACC AAGAATGTTT
TCATTAATCA AGAACGAAAG TTAGAGGTTC GAAGGCGATC AGATACCGCC CTAGTTCTAA
CCATAAACGA TGCCAACCAG TAATTCGCCT GAGTTCTAAT GACTCGGGAC TTCCGGGAAA
CCAAAGTTCG GTTCCAGGGG AAGTATGGTT GCAAAGCTGA AACTTAAAGA AATTGACGGA
AGGGCACCAC CAGGAGTGGA GCCTGCGGCT TAATTTGACT CAACACGGGA AAACTCACCC
GGCCCGGACA CTGGAAGGAT TGACAGATTG AGAGCTCTTT CTTGATTCAG TGGGTGGTGG
TGCATGGCCG TTCTTAGTTG GTGGAGTGAT TTGTCTGGTT AATTCCGATA ACGAACGAGA
CTCTAGCCTA CTAAATAACG CCGGCTTCTT AGAGGGACAA GCGGTGATTC AACCGCACGA
AACAGAGCAA TAACAGGTCT GTGATGCCCT TAGATGTCCG GGGCCGCACG CGCGCTACAC
TGAAAAGATC AGCGTGCTGC CTACTCTGTC AAGAGTGGGT AACCCAATGA ACCTTTGTGA
TTGGGATTTT GTAATTTTTC CACATGAACG AGGAATTCCC AGTAAGCGCG AGTCA
>Spin_sp
ATCAGTTACG GTTAGATATT GACTTTTACA TGGATAACTG TGGTAATTCT AGAGCTAATA
CATGCACAAA AGCTTCGACC TGGAAGAAGC GCATTTATTA GACCAAGACC AATGGGGGTG
GTGACTCTGA ATAACTGCTA ATCGCATGGC CGAGCCGGCG ATGAATTCAA GTGTCTGCCT
```

TATCAACTGT CGATGGTAGG TTATGCGCCT ACCATGGTTG TAACGGGTAA CGGGGAATTA
TTCGATTCCA GCCTTAAAGA CTACTACATC CAAGGAAGGC AGCAGGCACG CAAATTACCC
AAGGTAGTGA CGAAAAATAA CAATACAGGA CTCTTATGAG GCCCTGTAAT TGGAATGAGA
ACAATTTAAA TCCTTAACGA GGATCTATTG GAGGGCAAGT CTGGTGCCAG CAGCCGCGGT
AATCCAGCTC CAATAGCGTA TATTAAAGTT GTTGTGGTTA AAAAGCTCGT AGTTGGATCT
CAGTTCGAGT CAGCGGTCCA CTTGCCAGTG GTTACTGCTT TGAACATTAC CGCCTATGGT
GCTCTTCACC GAGTGTCATA GGCGATCGGT ACGTTTACTT TGAAAAAATT AGAGTGCTCA
AAGCAGGCGC CCGAATAATG TTGCATGGAA TAATAGAATA GGACCTCGGT TCTATTTTGT
TGGTCTTCGG AACTGAGGTA ATGATTAGAG GGACAGACGG GGGCATTCGT ATTGCGGCGC
TAGAGGTGAA ATTCTTGGAC CGTCGCAAGA CGAACTAAAG CGAAAGCACC AAGAATGTTT
TCATTAATCA AGAACGAAAG TTAGAGGTTC GAAGGCGATC AGATACCGCC CTAGTTCTAA
CCATAAACGA TGCCAACCAG TAATTCGCCT GAGTTCTAAT GACTCGGGAC TTCCGGGAAA
CCAAAGTTCG GTTCCAGGGG AAGTATGGTT GCAAAGCTGA AACTTAAAGA AATTGACGGA
AGGGCACCAC CAGGAGTGGA GCCTGCGGCT TAATTTGACT CAACACGGGA AAACTCACCC
GGCCCGGACA CTGGAAGGAT TGACAGATTG AGAGCTCTTT CTTGATTCAG TGGGTGGTGG
TGCATGGCCG TTCTTAGTTG GTGGAGTGAT TTGTCTGGTT AATTCCGATA ACGAACGAGA
CTCTAGCCTA CTAAATAACG CTGGCTTCTT AGAGGGACAG GCGGTGATTC AACCGCACGA
AACAGAGCAA TAACAGGTCT GTGATGCCCT TAGATGTCCG GGGCCGCACG CGCGCTACAC
TGAAGTGATC AGCGTGCTAC CTACTCTGTC AAGAGTGGGT AACCCAATGA ACCTTCGTGA
TTGGGATTTT GTAATTTTTC CACATGAACG AGGAATTCCC AGTAAGCGCG AGTCA
>Carabodes_coriaceus
ATCAGTTACG GTTAGATATT GACTTTTACA TGGATAACTG TGGTAATTCT AGAGCTAATA
CATGCGCAAA AGCTTCGACC TGGAAGAAGC GCATTTATTA GAACAAGACC AATGGGGGTG
GTGACTCTGA ATAACTGCTA ATCGCATGGC CGAGCCGGCG ATGAATTCAA GTGTCTGCCT
TATCAACTGT CGATGGTAGG TTATGCGCCT ACCATGGTTG TAACGGGTAA CGGGGAATCA
TTCGATTCCA GCCTGAGAAA CTACCACATC CAAGGAAGGC AGCAGGCGCG CAAATTATCC
GAGATAGTGA CGAAAAATAA CAATACAGGA CTCTTATGAG GCCCTGTAAT TGGAATGAGA
ACAATTTAAA TCCTTATCGA GGATCTATTG GAGGGCAAGT CTGGTGCCAG CAGCCGCGGT
AATCCAGCTC CAATAGCGTA TATTAAAGTT GTTGTGGTTA AAAAGCTCGT AGTTGGATCT
CAGTTCGAGT CGGCGGTCCA CCTGCCAGTG GTTACTGCTT GGAACATTAC CGCCTATGGT
GCTCTTCACC GAGTGTCATA GGCGATCGGT ACGTTTACTT TGAAAAAATT AGAGTGCTCA
AAGCAGGCGC CCGAATAATG TTGCATGGAA TAATGGAATA GGACCTCGGT TCTATTTTGT
TGGTCTTCGG AACTGAGGTA ATGATTAGAG GGACAGACGG GGGCATTCGT ATTGCGGCGC
TAGAGGTGAA ATTCTTGGAC CGTCGCACGA CGAACTAAAG CGAAAGCACC AAGAATGTTT
TCATTAATCA AGAACGAAAG TTAGAGGTTC GAAGGCGATC AGATACCGCC CTAGTTCTAA
CCATAAACGA TGCCAACCAG TAATTCGCCT GAGTTCTAAT GACTCGGGAC TTCCGGGAAA
CCAAAGTTCG GTTCCAGGGG AAGTATGGTT GCAAAGCTGA AACTTAAAGA AATTGACGGA
AGGGCACCAC CAGGAGTGGA GCCTGCGGCT TAATTTGACT CAACACGGGA AAACTCACCC
GGCCCGGACA CTGGAAGGAT TGACAGATTG AGAGCTCTTT CTTGATTCAG TGGGTGGTGG
TGCATGGCCG TTCTTAGTTG GTGGAGCGAT TTGTCTGGTT AATTCCGATA ACGAACGAGA
CTCTAGCCTA CTAAATAACG GTGGCTTCTT AGAGGGACAG GCGGTGATTT AACCGCACGA
AACAGAGCAA TAACAGGTCT GTGATGCCCT TAGATGTCCG GGGCCGCACG CGCGCTACAC
TGAAGTGATC AGCGTGCAGC CTACTCTGTC AAGAGTGGGT AACCCAATGA ACCTTCGTGA
TTGGGATTTT GTAATTTTTC CACATGAACG AGGAATTCCC AGTAAGCGCG AGTCA
>Carabodes_subarcticus
ATCAGTTACG GTTAGATATT GACTTTTACA TGGATAACTG TGGTAATTCT AGAGCTAATA
CATGCACAAA AGCTTCAACC TGGAAGAAGC GCATTTATTA GAACAAGACC AATGGGGGTG
GTGACTCTGG ATAACTGCTA ATCGCATGGC CGAGCCGGCG ATGAATTCAA GTGTCTGCCT
TATCAACTGT CGATGGTAGG TTATGCGCCT ACCATGGTTG TAACGGGTAA CGGGGAATAA
TTCGATTCCA GCCTGAGAAA CTACCACATC CAAGGAAGGC AGCAGGCGCG CAAATTATCC
GAGATAGTGA CGAAAAATAA CAATACAGGA CTCTTATGAG GCCCTGTAAT TGGAATGAGA
ACAATTTAAA TCCTTATCGA GGATCTATTG GAGGGCAAGT CTGGTGCCAG CAGCCGCGGT
AATCCAGCTC CAATAGCGTA TATTAAAGTT GTTGTGGTTA AAAAGCTCGT AGTTGGATCT
CAGTTCGAGT CAGCGGTCCG CTTGCCAGCG GTTACTGCTT GGAACATTAC CGCCTATGGT
GCTCTTCACC GAGTGTCATA GGCGATCGGT ACGTTTACTT TGAAAAAATT AGAGTGCTCA
AAGCAGGCGC CCGAATAATG TTGCATGGAA TAATGGAATA GGACCTCGGT TCTATTTTGT
TGGTCTTCGG AACTGAGGTA ATGATTAGAG GGACAGACGG GGGCATTCGT ATTGCGGCGC
TAGAGGTGAA ATTCTTGGAC CGTCGCACGA CGAACTAAAG CGAAAGCACC AAGAATGTTT
TCATTAATCA AGAACGAAAG TTAGAGGTTC GAAGGCGATC AGATACCGCC CTAGTTCTAA
CCATAAACGA TGCCAACCAG TAATTCGCCT GAGTTCAAAT GACTCGGGAC TTCCGGGAAA

CCAAAGTTCG GTTCCAGGGG AAGTATGGTT GCAAAGCTGA AACTTAAAGA AATTGACGGA
AGGGCACCAC CAGGAGTGGA GCCTGCGGCT TAATTTGACT CAACACGGGA AAACTCACCC
GGCCCGGACA CTGGAAGGAT TGACAGATTG AGAGCTCTTT CTTGATTCAG TGGGTGGTGG
TGCATGGCCG TTCTTAGTTG GTGGAGCGAT TTGTCTGGTT AATTCCGATA ACGAACGAGA
CTCTAGCCTA CTAAATAACG GTGGCTTCTT AGAGGGACAG GCGGTGATTT AACCGCACGA
AACAGAGCAA TAACAGGTCT GTGATGCCCT TAGATGTCCG GGGCCGCACG CGCGCTACAC
TGAAGTGATC AGCGTGCAGC CTACTCTGTC AAGAGTGGGT AACCCAATGA ACCTTCGTGA
TTGGGATTTT GTAATTTTTC CACATGAACG AGGAATTCCC AGTAAGCGCG AGTCA
>Carabodes_labyrinthicus
ATCAGTTACG GTTAGATATT GACTTTTACA TGGATAACTG TGGTAATTCT AGAGCTAATA
CATGCACAAA AGCTTCGACC TGGAAGAAGC GCATTTATTA GAACAAGACC AATGGGGGTG
GTGACTCTGG ATAACTGCTA ATCGCATGGC CGAGCCGGCG ATGAATTCAA GTGTCTGCCT
TATCAACTGT CGATGGTAGG TTATGCGCCT ACCATGGTTG TAACGGGTAA CGGGGAATCA
TTCGATTCCA GCCTGAGAAA CTACCACATC CAAGGAAGGC AGCAGGCGCG CAAATTATCC
GAGATAGTGA CGAAAAATAA CAATACAGGA CTCTTATGAG GCCCTGTAAT TGGAATGAGA
ACAATTTAAA TCCTTATCGA GGATCTATTG GAGGGCAAGT CTGGTGCCAG CAGCCGCGGT
AATCCAGCTC CAATAGCGTA TATTAAAGTT GTTGTGGTTA AAAAGCTCGT AGTTGGATCT
CAGTTCGAGT CGGCGGTCCA CTTGCCAGTG GTTACTGCTT GGAACATTAC CGCCTATGGT
GCTCTTCACC GAGTGTCATA GGCGATCGGT ACGTTTACTT TGAAAAAATT AGAGTGCTCA
AAGCAGGCGC CCGAATAATG TTGCATGGAA TAATGGAATA GGACCTCGGT TCTATTTTGT
TGGTCTTCGG AACTGAGGTA ATGATTAGAG GGACAGACGG GGGCATTCGT ATTGCGGCGC
TAGAGGTGAA ATTCTTGGAC CGTCGCACGA CGAACTAAAG CGAAAGCACC AAGAATGTTT
TCATTAATCA AGAACGAAAG TTAGAGGTTC GAAGGCGATC AGATACCGCC CTAGTTCTAA
CCATAAACGA TGCCAACCAG TAATTCGCCT GAGTTCTAAT GACTCGGGAC TTCCGGGAAA
CCAAAGTTCG GTTCCAGGGG AAGTATGGTT GCAAAGCTGA AACTTAAAGA AATTGACGGA
AGGGCACCAC CAGGAGTGGA GCCTGCGGCT TAATTTGACT CAACACGGGA AAACTCACCC
GGCCCGGACA CTGGAAGGAT TGACAGATTG AGAGCTCTTT CTTGATTCAG TGGGTGGTGG
TGCATGGCCG TTCTTAGTTG GTGGAGCGAT TTGTCTGGTT AATTCCGATA ACGAACGAGA
CTCTAGCCTA CTAAATAACG GTGGCTTCTT AGAGGGACAG GCGGTGATTT AACCGCACGA
AACAGAGCAA TAACAGGTCT GTGATGCCCT TAGATGTCCG GGGCCGCACG CGCGCTACAC
TGAAGTGATC AGCGTGCAGC CTACTCTGTC AAGAGTGGGT AACCCAATGA ACCTTCGTGA
TTGGGATTTT GTAATTTTTC CACATGAACG AGGAATTCCC AGTAAGCGCG AGTCA
>Carabodes_sp
ATCAGTTACG GTTAGATATT GACTTTTACA TGGATAACTG TGGTAATTCT AGAGCTAATA
CATGCGCAAA AGCTTCGACC TGGAAGAAGC GCATTTATTA GAACAAGACC AATGGGGGTG
GTGACTCTGA ATAACTGCTA ATCGCATGGC CGAGCCGGCG ATGAATTCAA GTGTCTGCCT
TATCAACTGT CGATGGTAGG TTATGCGCCT ACCATGGTTG TAACGGGTAA CGGGGAATCA
TTCGATTCCA GCCTGAGAAA CTACCACATC CAAGGAAGGC AGCAGGCGCG CAAATTATCC
GAGATAGTGA CGAAAAATAA CAATACAGGA CTCTTATGAG GCCCTGTAAT TGGAATGAGA
ACAATTTAAA TCCTTATCGA GGATCTATTG GAGGGCAAGT CTGGTGCCAG CAGCCGCGGT
AATCCAGCTC CAATAGCGTA TATTAAAGTT GTTGTGGTTA AAAAGCTCGT AGTTGGATCT
CAGTTCGAGT CGGCGGTCCA CCTGCCAGTG GTTACTGCTT GGAACATTAC CGCCTATGGT
GCTCTTCACC GAGTGTCATA GGCGATCGGT ACGTTTACTT TGAAAAAATT AGAGTGCTCA
AAGCAGGCGC CCGAATAATG TTGCATGGAA TAATGGAATA GGACCTCGGT TCTATTTTGT
TGGTCTTCGG AACTGAGGTA ATGATTAGAG GGACAGACGG GGGCATTCGT ATTGCGGCGC
TAGAGGTGAA ATTCTTGGAC CGTCGCACGA CGAACTAAAG CGAAAGCACC AAGAATGTTT
TCATTAATCA AGAACGAAAG TTAGAGGTTC GAAGGCGATC AGATACCGCC CTAGTTCTAA
CCATAAACGA TGCCAACCAG TAATTCGCCT GAGTTCTAAT GACTCGGGAC TTCCGGGAAA
CCAAAGTTCG GTTCCAGGGG AAGTATGGTT GCAAAGCTGA AACTTAAAGA AATTGACGGA
AGGGCACCAC CAGGAGTGGA GCCTGCGGCT TAATTTGACT CAACACGGGA AAACTCACCC
GGCCCGGACA CTGGAAGGAT TGACAGATTG AGAGCTCTTT CTTGATTCAG TGGGTGGTGG
TGCATGGCCG TTCTTAGTTG GTGGAGCGAT TTGTCTGGTT AATTCCGATA ACGAACGAGA
CTCTAGCCTA CTAAATAACG GTGGCTTCTT AGAGGGACAG GCGGTGATTT AACCGCACGA
AACAGAGCAA TAACAGGTCT GTGATGCCCT TAGATGTCCG GGGCCGCACG CGCGCTACAC
TGAAGTGATC AGCGTGCAGC CTACTCTGTC AAGAGTGGGT AACCCAATGA ACCTTCGTGA
TTGGGATTTT GTAATTTTTC CACATGAACG AGGAATTCCC AGTAAGCGCG AGTCA
>Plenotocepheus_neotropicus
ATCAGTTACG GTTAGATATT GACTTTTACA TGGATAACTG TGGTAATTCT AGAGCTAATA
CATGCACAAA AGCTTCGACC TGGAAGAAGC GCATTTATTA GAACAAGACC AATGGGGGTG
GTGACTCTGG ATAACTGCTG ATCGCATGGC CGAGCCGGCG ATAAATTCAA GTGTCTGCCT

TATCAACTGT CGATGGTAGG TTATGTGCCT ACTATGGTTA TAACGGGTAA CGGGGAATTA
TTCGATTCCA GCCTTAAAAA CTACCACATC CAAGGAAGGC AGCAGGCACG CAAATTACCC
AAGGTAGTGA CGAAAAATAA CAATACAGGA CTCTTATGAG GCCCTGTAAT TGGAATGAGA
ACAATTTAAA TCCTTATCGA GGATCTATTG GAGGGCAAGT CTGGTGCCAG CAGCCGCGGT
AATCCAGCTC CAATAGCGTA TATTAAAGTT GTTGTGGTTA AAAAGCTCGT AGTTGGATCT
CAGTTCGAGT CAGCGGTCCA CTTGCCAGTG GCTACTGCTT TGAACATTAC CGCCTATGGT
GCTCTTTACC GAGTGTCATA GGCGATCGGT ACGTTTACTT TGAAAAAATT AGAGTGCTCA
AAGCAGGCGC CCGAATAATG TTGCATGGAA TAATGGAATA GGACCTCGGT TCTATTTTGT
TGGTCTTCGG AACTGAGGTA ATGATTAGAG GGACAGACGG GGGCATTCGT ATTGCGGCGC
TAGAGGTGAA ATTCTTGGAC CGTCGCAAGA CGAACTAAAG CGAAAGCACC AAGAATGTTT
TCATTAATCA AGAACGAAAG TTAGAGGTTC GAAGGCGATC AGATACCGCC CTAGTTCTAA
CCATAAACGA TGCCAACCAG TAATTCGCCT GAGTTCTAAT GACTCGGGAC TTCCGGGAAA
CCAAAGTTCG GTTCCAGGGG AAGTATGGTT GCAAAGCTGA AACTTAAAGA AATTGACGGA
AGGGCACCAC CAGGAGTGGA GCCTGCGGCT TAATTTGACT CAACACGGGA AAACTCACCC
GGCCCGGACA CTGGAAGGAT TGACAGATTG AGAGCTCTTT CTTGATTCAG TGGGTGGTGG
TGCATGGCCG TTCTTAGTTG GTGGAGTGAT TTGTCTGGTT AATTCCGATA ACGAACGAGA
CTCTAGCCTA TTAAATAACG CTGGCTTCTT AGAGGGACAG GCGGTGATTC AACCGCACGA
AACAGAGCAA TAACAGGTCT GTGATGCCCT TAGATGTTCG GGGCCGCACG CGCGCTACAC
TGAAGTGATC AGCATGCATC CTACTCTGTC AAGAGTGGGT AACCCAATGA ACCTTCGTGA
TTGGGATTTT GTAATTTTTC CACATGAACG AGGAATTCCC AGTAAGCGCG AGTCA
>Beckiella_capitulum
ATCAGTTACG GTTAGATGTT GACATTTACA TGGATAACTG TGGTAATTCT AGAGCTAATA
CATGCATAAA AGCTTCGACT TGGGAGAAGC GCATTTATTA GACCAAGACC AATGGGGGTG
GTGACTCTGG ATAACTGCTG ATCGCATGGC CGAGCCGGCG ACGAATTCAA GTGTCTGCCT
TATCAACTGT CGATGGTAGG TTATGCGCCT ACCATGGTTG TAACGGGTAA CGGGGAATCA
TTCGATTCCA GCCCTAGAAA CTACCACATC CAAGGAAGGC AGCAGGCACG CAAATTACCC
AAGGTAGTGA CGAAAAATAA CAATACAGGA CTCTTATGAG GCCCTGTAAT TGGAATGAGA
ACAATTTAAA TCCTTAACGA GGATCTATTG GAGGGCAAGT CTGGTGCCAG CAGCCGCGGT
AATCCAGCTC CAATAGCGTA TATTAAAGTT GTTGTGGTTA AAAAGCTCGT AGTTGGATCT
CAGTTCGAGT CGACGGTCCA CTTGCCAGTG GTTACTGTCT TGAACATTAC CGCCTATGGT
GCTCTTCGCC GGGTGTCATA GGCGATCGGT ACGTTTACTT TGAAAAAATT AGAGTGCTCA
AAGCAGGCGC CCGAATAATG TTGCATGGAA TAATGGAATA GGACCTCGGT TCTATTTTGT
TGGTCTTCGG AACTGAGGTA ATGATTAGAG GGACAGACGG GGGCATTCGT ATTGCGGCGC
TAGAGGTGAA ATTCTTGGAC CGTCGCACGA CGAACTAAAG CGAAAGCACC AAGAATGTTT
TCATTAATCA AGAACGAAAG TTAGAGGTTC GAAGGCGATC AGATACCGCC CTAGTTCTAA
CCATAAACGA TGCCAACCAG TAATTCGCCT GAGTTTTAAT GACTCGGGAC TTCAGGGAAA
CCAAAGTTCG GTTCCAGGGG AAGTATGGTT GCAAAGCTGA AACTTAAAGA AATTGACGGA
AAGGCACCAC AAGGAGTGGA GCCTGCGGCT TAATTTGACT CAACACGGGA AAACTCACCC
GGCCCGGACA CTGGAAGGAT TGACAGATTG AGAGCTCTTT CTTGATTCAG TGGGTAGTGG
TGCATGGCCG TTCTTAGTTG GTGGAGTGAT TTGTCTGGTT AATTCCGATA ACGAACGAGA
CTCTAGCCTA CTAAATAACG CCGGCTTCTT AGAGGGACAG GCGGTGATTC AACCGCATGA
AACAGAGCAA TAACAGGTCT GTGATGCCCT TAGATGTCCG GGGCCGCACG CGCGCTACAC
TGAAGTGATC AGCGTGCTGC CTACTCTGTC AAGAGTGGGT AACCCAATGA ACCTTCGTGA
TTGGGATTTT GTAATTATTC CACATGAACG AGGAATTCCT AGTAAGCGCG AGTCA
>Beckiella_arcta
ATCAGTTACG GTTAGATGTT GACATTTACA TGGATAACTG TGGTAATTCT AGAGCTAATA
CATGCACAAA AGCTTCAACT TGGGAGAAGC GCATTTATTA GACCAAGACC AATGGGGGTG
GTGACTCTGG ATAACTGCTG ATCGCATGGC CGTGCCGGCG ACGAATTCAA GTGTCTGCCT
TATCAACTGT CGATGGTAGG TTATGCGCCT ACCATGGTTG TAACGGGTAA CGGGGAATCA
TTCGATTCCA GCCCGAGAAA CTACCACATC CAAGGAAGGC AGCAGGCACG CAAATTACCC
AAGGTAGTGA CGAAAAATAA CAATACAGGA CTCTTATGAG GCCCTGTAAT TGGAATGAGA
ACAATTTAAA TCCTTAACGA GGATCTATTG GAGGGCAAGA CTGGTGCCAG CAGCCGCGGT
AATCCAGCTC CAATAGCGTA TATTAAAGTT GTTGTGGTTA AAAAGCTCGT AGTTGGATCT
CAGTTCGAGT CGACTTTCCA CTTGCCAGTG GTTACTGTCT TGAACATTAC CGCCTATGGT
GCTCTTCGCC GGGTGTCATA GGCGATCGGT ACGTTTACTT TGAAAAAATT AGAGTGCTCA
AAGCAGGCGC CCGAATAATG TTGCATGGAA TAATGGAATA GGACCTCGGT TCTATTTTGT
TGGTCTTCGG AACTGAGGTA ATGATTAGAG GGACAGACGG GGGCATTCGT ATTGCGGCGC
TAGAGGTGAA ATTCTTGGAC CGTCGCACGA CGAACTAAAG CGAAAGCACC AAGAATGTTT
TCATTAATCA AGAACGAAAG TTAGAGGTTC GAAGGCGATC AGATACCGCC CTAGTTCTAA
CCATAAACGA TGCCAACCAG TAATTCGCCT GAGTTTTAAT GACTCGGGAC TTCAGGGAAA

CCAAAGTTCG GTTCCAGGGG AAGTATGGTT GCAAAGCTGA AACTTAAAGA AATTGACGGA
AAGGCACCAC AAGGAGTGGA GCCTGCGGCT TAATTTGACT CAACACGGGA AAACTCACCC
GGCCCGGACA CTGGAAGGAT TGACAGATTG AGAGCTCTTT CTTGATTCAG TGGGTAGTGG
TGCATGGCCG TTCTTAGTTG GTGGAGTGAT TTGTCTGGTT AATTCCGATA ACGAACGAGA
CTCTAGCCTA CTAAATAACG CCGGCTTCTT AGAGGGACAG GCGGTGATTC AACCGCATGA
AACAGAGCAA TAACAGGTCT GTGATGCCCT TAGATGTCCG GGGCCGCACG CGCGCTACAC
TGAAGTGATC AGCGTGCTGC CTACTCTGTC AAGAGTGGGT AACCCAATGA ACCTTCGTGA
TTGGGATTTT GTAATTATTC CACATGAACG AGGAATTCCT AGTAAGCGCG AGTCA
>Ceratoppia_bipilis
ATCAGTTACG GTTAGATGTT GACATCTACA TGGATAACTG TGGTAATTCT AGAGCTAATA
CATGCACAAA AGCTTCGACC TGGAAGAAGC GCATTTATTA GACCAAGACC AATGGGGGTG
GTGACTCTGG ATAACTGCTA ATCGCATGGC CGTGCCGGCG ATGAATTCAA GTGTCTGCCT
TATCAACTGT CGATGGTAGG TTATGCGCCT ACCATGGTTG TAACGGGTAA CGGGGAATCA
TTCGATTCCA GCCTGAGAAA CTACCACATC CAAGGAAGGC AGCAGGCACG CAAATTACCC
AAGGTAGTGA CGAAAAATAA CAATACGGGA CTCTTATGAG GCCCCGTAAT TGGAATGAGA
ACAATTTAAA TCCTTAACGA GGATCTATTG GAGGGCAAGT CTGGTGCCAG CAGCCGCGGT
AATCCAGCTC CAATAGCGTA TATTAAAGTT GTTGTGGTTA AAAAGCTCGT AGTTGGATCT
CAGTTCGAGT CGGCGGTCCA CTTGCCAGTG GTTACTGCTT TGAACATTAT CGCCTATGGT
GCTCTTCACC GAGTGTCATA GGCGATCGAT ACGTTTACTT TGAAAAAATT AGAGTGCTCA
AAGCAGGCGC CCGAATAATG TTGCATGGAA TAATGGAATA GGACCTCGGT TCTATTTTGT
TGGTCTTCGG AACTGAGGTA ATGATTAGAG GGACAGACGG GGGCATTCGT ATTGCGGCGC
TAGAGGTGAA ATTCTTGGAC CGTCGCAAGA CGAACTAAAG CGAAAGCACC AAGAATGTTT
TCATTAATCA AGAACGAAAG TTAGAGGTTC GAAGGCGATC AGATACCGCC CTAGTTCTAA
CCATAAACGA TGCCAACCAG TGATAAGCCT GAGTTCAAAT GACTCGTAAC TTCCGGGAAA
CCAAAGTTCG GTTCCAGGGG AAGTATGGTT GCAAAGCTGA AACTTAAAGA AATTGACGGA
AGGGCACCAC CAGGAGTGGA GCCTGCGGCT TAATTTGACT CAACACGGGA AAACTCACCC
GGCCCGGACA CTGGAAGGAT TGACAGATTG AGAGCTCTTT CTTGATTCAG TGGGTGGTGG
TGCATGGCCG TTCTTAGTTG GTGGAGCGAT TTGTCTGGTT AATTCCGATA ACGAACGAGA
CTCTAGCCTA CTAAATAACG CTGACTTCTT AGAGGGACAG GCGGTGATTC AACCGCACGA
AACAGAGCAA TAACAGGTCT GTGATGCCCT TAGATGTCCG GGGCCGCACG CGCGCTACAC
TGAAGTGATC AGCGTGCAGC CTACTCTGTC AAGAGTGGGA AACCCAATGA ACCTTCGTGA
TTGGGATATT GTAATTATTC CCCTTGAACG AGGAATTCCC AGTAAGCGCG AGTCA
>Liacarus_coracinus
ATCAGTTACG GTTAGATGTT GACATCTACA TGGATAACTG TGGTAATTCT AGAGCTAATA
CATGCACAAA AGCTTCGACC TGGAAGAAGC GCATTTATTA GACCAAGACC AATGGGGGTG
GTGACTCTGA ATAACTGCTA ATCGCATGGC CGTGCCGGCG ATGAATTCAA GTGTCTGCCT
TATCAACTGT CGATGGTAGG CTATGCGCCT ACCATGGTTG TAACGGGTAA CGGGGAATCA
TTCGATTCCA GCCTGAGAAA CTACCACATC CAAGGAAGGC AGCAGGCACG CAAATTACCC
AAGGTAGTGA CGAAAAATAA CAATACGGGA CTCTTATGAG GCCCCGTAAT TGGAATGACA
ACAATCTAAA TCCTTAACGA GGATCTATTG GAGGGCAAGT CTGGTGCCAG CAGCCGCGGT
AATCCAGCTC CAATAGCGTA TATTAAAGTT GTTGTGGTTA AAAAGCTCGT AGTTGGATCT
CAGTTCGAGT TGGTGGTCCA CTTGCCAGTG GTTACTACCT TGAACATTAT CGCTAATGGT
GCTCTTCACC GAGTGTCATT AGCGATCGAT ACGTTTACTT TGAAAAAATT AGAGTGCTCA
AAGCAGGCGC CCGAATAATG TTGCATGGAA TAATGGAATA GGACCTCGGT TCTATTTTGT
TGGTCTTCGG AACTGAGGTA ATGATTAGAG GGACAGACGG GGGCATTCGT ATTGCGGCGC
TAGAGGTGAA ATTCTTGGAC CGTCGCAAGA CGAACTAAAG CGAAAGCACC AAGAATGTTT
TCTTTAATCA AGAACGAAAG TTAGAGGTTC GAAGGCGATC AGATACCGCC CTAGTTCTAA
CCATAAACGA TGCCGACCAG TGATTAGACT GAGTTCAAAT GACTCGCAAC TTCCGGGAAA
CCAAAGTTCG GTTCCAGGGG AAGTATGGTT GCAAAGCTGA AACTTAAAGA AATTGACGGA
AGGGCACCAC CAGGAGTGGA GCCTGCGGCT TAATTTGACT CAACACGGGA AAACTCACCC
GGCCCGGACA CTGGAAGGAT TGACAGATTG AGAGCTCTTT CTTGATTCAG TGGGTGGTGG
TGCATGGCCG TTCTTAGTTG GTGGAGTGAT TTGTCTGGTT AATTCCGATA ACGAACGAGA
CTCTAGCCTA CTAAATAACG CTAACTTCTT AGAGGGACAG GCGGTGATTC AACCGCACGA
AACAGAGCAA TAACAGGTCT GTGATGCCCT TAGATGTCCG GGGCCGCACG CGCGCTACAC
TGAAGTGATC AGCGTGCAGC CTACTCTGTC AAGAGTGGGA AACCCAATGA ACCTTCGTGA
TTGGGATATT GTAATTGTTC CCCTTGAACG AGGAATTCCC AGTAAGCGCG AGTCA
>Globoppia_maior
ATCAGTTACG GTTTGATGGT GACGTTTACA TGGATAACTG TGGTAATTCT AGAGCTAATA
CATGCCCTAA AGCTTCGACC GGGGAGAAGT GCATTTATTA GAACAAGACC AATGGGGGTG
GTGACTCTGG ATAACTGCTA ATCGTATGGC CGTGCCGACG ATAAATTCAA GTGTCTGCCT

TATCAACTGT CGATGGTAGG TCATGCGCCT ACCATGGTTG TAACGGGTAA CGGGGAATTA
TTCGATTCCA GCCTGAGAAA CTAGCACATC CAAGGAAGGC AGCAGGCGTG CAAATTACCC
AAGGTAGTGA CGAAAAATAA CAATACGAGA CTCTTATGAG GCCTCGTAAT TGGAATGAGA
ACAATATAAA TCACTAACGA GGATCTATTG GAGGGCAAGT CTGGTGCCAG CAGCCGCGGT
AATCCAGCTC CAATAGCGTA TATTAAAGTT GTTGTGGTTA AAAAGCTCGT AGTTGGATCT
CAGTTCTAGT CGACGGTCCA CTTGCCAGTG GTTACTGTTT TGAACATTAC CGCCTATGGT
GCTCTTCACC GAGTGTCATA GGCGATCGGT ACGTTTACTT TGAAAAAATT AGAGTGCTCA
AAGCAGGCGC CCGAATAATG TTGCATGGAA TAATGGAATA GGACCTCGGT TCTATTTTGT
TGGTCTTCGG AGATGAGGTA ATGATCATAG GGACAGACGG GGCCATTCGT ATTGCGGAGC
TAGAGGTGAA ATTCTTGGAC CTTCGCAAGA CGAACTAAAG CGAAAGCAGC AAGAATGTTT
TCATTAATCA AGAACGAAAG TTAGAGGTTC GAAGGCGATC AGATACCGCC CTAGTTCTAA
CCATAAACGA TGCCGACCAG TAATTCGCCT GAGTTCAAAT GACTCGGGGC TTCCGGGAAA
CCAAAGTTCG GTTCCAGGGG AAGTATGGTT GCAAAGCTGA AACTTAAAGA AATTGACGGA
AGGGCACCAC CAGGAGTGGA GCCTGCGGCT TAATTTGACT CAACACGGGA AAACTCACCC
GGCCCGGACA CTAGAAGGAT TGACAGATTG AGAGCTCTTT CTTGATTCAG TGGGTGGTGG
TGCATGGCCG TTCTTAGTTG GTGGAGTGAT TTGTCTGGTT AATTCCGATA ACGAACGAGA
CTCTAGCCTA TTAAATAACG TTGACTTCTT AGAGGGACAG GCGGTGTTTC AACCGCACGA
AACAGAGCAA TAACAGGTCT GTGATGCCCT TAGATGTCCG GGGCCGCACG CGCGCTACAC
TGAAGTGATC AGCGTGCAGC CTACTCTGTC AAGAGTGGGG AACCCAATGA ACCTTCGTGA
TTGGGATATT GTAATTATTC CCCTTGAACG AGGAATTCCC AGTAAGCGCG AGTCA
>Gittella_variabilis
ATCAGTTACG GTTAGATATT GACGTTTACA TGGATAACTG TGGTAATTCT AGAGCTAATA
CATGCACAAA AGCTTCGACC TGGAAGAAGT GCATTTATTA GAACAAGACC AATGGGGGTG
GTGACTCTGG ATAACTGCTA ATCGTATGGC CGCGCCGGCG ATGAATTCAA GTGTCTGCCT
TATCAACTGT CGATGGTAGG TTATGCGCCT ACTATGGTTG TAACGGGTAA CGGGGAATCA
TTCGATTCCA GCATGAGAAT CTACCACATC CAAGGAAGGC AGCAGGCACG CAAATTACCC
AAGGTAGTGA CGAAAAATAA CAATACAAGT CTCTTATGAG GCCTTGTAAT TGGAATGAGA
ACAATATAAA TGACTAACGA GGATCTATTG GAGGGCAAGT CTGGTGCCAG CAGCCGCGGT
AATCCAGCTC CAATAGCGTA TATTAAAGTT GTTGTGGTTA AAAAGCTCGT AGTTGGATCT
CAGTTCAAGT CGACGGTCCA CTTGATAGTG GTTACTGTTT TGAACATTAT CGCCTATGGT
GCTCTTCACC GAGTGTCATA GGCGATCGAT ACGTTTACTT TGAAAAAATT AGAGTGCTTA
AAGCAGGCGC CCGAATAATG TTGCATGGAA TAATGGAATA GGACCTCGGT TCTATTTTGT
TGGTCTTCGG AGTTGAGGTA ATGATTAGAG GGACAGTTGG GGCCATTTGT ATTGCGTAGA
CAGAGGTGAA ATTCTTGGAC CTATGCAAGA CAAACTAAAG CGAAAGCAGC AAGAATGTTT
TCATTAATCA AGAACGAAAG TTAGAGGTTC GAAGGCGATC AGATACCGCC CTAGTTCTAA
CCATAAACGA TGCCAACCAG TAATTCGCCT GAGTTCAAAT GACTCGGGAC TTCCGGGAAA
CCAAAGTTCG GTTCCAGGGG AAGTATGGTT GCAAAGCTGA AACTTAAAGA AATTGACGGA
AGGGCACCAC CAGGAGTGGA GCCTGCGGCT TAATTTGACT CAACACGGGA AAACTCACCC
GGCCCGGACA CTGTAAGGAT TGACAGATTG AGAGCTCTTT CTTGATTCAG TGGGTGGTGG
TGCATGGCCG TTCTTAGTTG GTGGAGTGAT TTGTCTGGTT AATTCCGATA ACGAACGAGA
CTCTAGCCTA CCAAATAACG TTGACTTCTT AGAGGGACAG GCGGTGATTC AACCGCACGA
AACAGAGCAA TAACAGGTCT GTGATGCCCT TAGATGTCCG GGGCCGCACG CGCGCTACAC
TGAAGTGATC AGCGTGCTGC CTACTCCGTC AGGAGTGGGG AACCCAATGA ACCTTCGTGA
TTGGGATATT GTAATTATTC TCCTTGAACG AGGAATTCCC AGTAAGTGCG TTTCA
>Epieremulus_granulatus
ATCAGTTACG GTTAGATGTT GACATCTACA TGGATAACTG TGGTAATTCT AGAGCTAATA
CATGCACAAA AGCTTCGACC TGGAAGAAGC GCATTTATTA GACCAAGACC AATGGGGGTG
GTGACTCTGA ATAACTGCTA ATCGCATGGC CGAGCCGGCG ATGAATTCAA GTGTCTGCCT
TATCAACTGT CGATGGTAGG TTATGTGCCT ACCATGGTTG TAACGGGTAA CGGGGAATCA
TTCGATTCCA GCATTAAAAT CTACCACATC CAAGGAGGGC AGCAGGCGCG CAAATTACCC
AAGGTAGTGA CGAAAAATAA CGATACAGGA CTCTTATGAG GCCCTGTAAT TGGAATGAGA
ACAATCTAAA TCCTTAACGA GGATCTATTG GAGGGCAAGT CTGGTGCCAG CAGCCGCGGT
AATCCAGCTC CAATAGCGTA TATTAAAGTT GTTGTGGTTA AAAAGCTCGT AGTTGGATCT
CAGTTCGAGT CGACGGTCCA CTTGCCAGTG GTTACTGTTT GGAACATTAC CGCCTATGGT
GCTTTTCACC GAGTGTCATA GGCGATCGGT ACGTTTACTT TGAAAAAATT AGAGTGCTCA
AAGCAGGCGC CCGAATAATG TTGCATGGAA TAATGGAATA GGACCTCGGT TCTATTTTGT
TGGTCTTCGG AACTGAGGTA ATGATTAGAG GGACAGACGG GGGCATTCGT ATTGCACCGC
TAGAGGTGAA ATTCTTGGAC CGGTGCAAGA CGAACTAAAG CGAAAGCACC AAGAATGTTT
TCATTAATCA AGAACGAAAG TTAGAGGTTC GAAGGCGATC AGATACCGCC CTAGTTCTAA
CCATAAACGA TGCCAACCAG CAATTCGCCT GAGTTCTAAT GACTCGGGGC TTCCGGGAAA

CCAAAGTTCG GTTCCAGGGG AAGTATGGTT GCAAAGCTGA AACTTAAAGA AATTGACGGA
AGGGCACCAC CAGGAGTGGA GCCTGCGGCT TAATTTGACT CAACACGGGA AAACTCACCC
GGCCCGGACA CTGGAAGGAT TGACAGATTG AGAGCTCTTT CTTGATTCAG TGGGTGGTGG
TGCATGGCCG TTCTTAGTTG GTGGAGTGAT TTGTCTGGTT AATTCCGATA ACGAACGAGA
CTCTAGCCTA CTAAATAACG CTGACTTCTT AGAGGGACAA GCGGTGATTC AACCGCACGA
AACAGAGCAA TAACAGGTCT GTGATGCCCT TAGATGTCCG GGGCCGCACG CGCGCTACAC
TGAAGCAATC AGCGTGCTGC CTACTCTGTC AAGAGTGGGA AACCCAATGA ACCTACGTGA
TTGGGATTTT GTAATTTTTC CACATGAACG AGGAATTCCC AGTAAGCGCG AGTCA
>Oppiella_nova
ATCAGTTACG GTTAGATGTT GACGTCTACA TGGATAACTG TGGTAATTCT AGAGCTAATA
CATGCACAAA AGCTTTGACC TGGAAAGAGC GCATTTATTA GAACAAGACC AATGGGGGTG
GTGACTCTGG ATAACTGCTA ATCACATGGC CGTGCCGGTG ATGAATTCAA GTGTCTGCCT
TATCAACTGT CGATGGTAGG TTATGTGCCT ACCATGGTTG TAACGGGTAA CGGGGAATCA
TTCGATTCCA GCCTGAGAAA CTACCACATC CAAGGAAGGC AGCAGGCACG CAAATTACCC
AAGGTAGTGA CGAAAAATAA CAATACGGGA CTCTTATGAG GCCCCGTAAT TGGAATGAGA
ACAATCTAAA TCCTTAACGA GGATCTATTG GAGGGCAAGT CTGGTGCCAG CAGCCGCGGT
AATCCAGCTC CAATAGCGTA TATTAAAGTT GTTGTGGTTA AAAAGCTCGT AGTTGGATCT
CAGTTCGAGT CGACGGTCCA CTTGCCAGTG GCTACTGTTT TGAACATTAC CGCCTATGGT
GTTCTTCACC GAGCGTCATA GGCGATCGGT ACGTTTACTT TGAAAAAATT AGAGTGCTCA
AAGCAGGCGC CCGAATAATG TTGCATGGAA TAATGGAATA GGACCTCGGT TCTATTTTGT
TGGTCTTCGG AACTGAGGTA ATGATCATAG GGACAGACGG GGCGATTCGT ATTGCGGCGC
TAGAGGTGAA ATTCTTGGAC CGTCGCAAGA CGAACTAAAG CGAAAGCAGC AAGAATGTTT
TCATTAATCA AGAACGAAAG TTAGAGGTTC GAAGGCGATC AGATACCGCC CTAGTTCTAA
CCATAAACGA TGCCAACCAG TAATTAAGCT GTGTTCAAAT GACACGGGAC TTCCGGGAAA
CCAAAGTTCG GTTCCAGGGG AAGTATGGTT GCAAAGCTGA AACTTAAAGA AATTGACGGA
AGGGCACCAC CAGGAGTGGA GCCTGCGGCT TAATTTGACT CAACACGGGA AAACTCACCC
GGCCCGGACA CTGGAAGGAT TGACAGATTG AGAGCTCTTT CTTGATTCAG TGGGTGGTGG
TGCATGGCCG TTCTTAGTTG GTGGAGTGAT TTGTCTGGTT AATTCCGATA ACGAACGAGA
CTCTAGCCTA CTAAATAACG CCGACTTCTT AGAGGGACAG GCGGTGATTC AACCGCACGA
AACAGAGCAA TAACAGGTCT GTGATGCCCT TAGATGTCCG GGGCCGCACG CGCGCTACAC
TGAAGTGATC AGCGTGCAAC CTACTCTGTC AAGAGTGGGG AACCCAATGA ACCTTCGTGA
TTGGGATATT GTAATTATTC CCCTTGAACG AGGAATTCCC AGTAAGCGCG AGTCA
